# Global burden of head and neck cancers from 1990 to 2019

**DOI:** 10.1016/j.isci.2024.109282

**Published:** 2024-02-20

**Authors:** Tianjiao Zhou, Weijun Huang, Xiaoting Wang, Jingyu Zhang, Enhui Zhou, Yixing Tu, Jianyin Zou, Kaiming Su, Hongliang Yi, Shankai Yin

**Affiliations:** 1 Department of Otorhinolaryngology Head and Neck Surgery, Shanghai Sixth People’s Hospital Affiliated to Shanghai Jiao Tong University School of Medicine, Shanghai, China; 2 Shanghai Key Laboratory of Sleep Disordered Breathing, Shanghai, China; 3 Otolaryngology Institute of Shanghai Jiao Tong University, Shanghai, China; 4 Department of Pharmacy, Shanghai Sixth People’s Hospital Affiliated to Shanghai Jiao Tong University School of Medicine, Shanghai, China

**Keywords:** Biological sciences, Cancer

## Abstract

Head and neck cancer (HNC) exerts a significant healthcare burden worldwide. Insufficient data impedes a comprehensive understanding of its global impact. Through analysis of the 2019 Global Burden of Disease (GBD) database, our secondary investigation unveiled a surging global incidence of HNC, yet a decline in associated mortality and disability-adjusted life years (DALYs) owing to enhanced prognosis. Particularly noteworthy is the higher incidence of escalation among females compared to males. Effective resource allocation, meticulous control of risk factors, and tailored interventions are imperative to curtail mortality rates among young individuals afflicted with HNC in underprivileged regions, as well as in elderly individuals grappling with thyroid cancer.

## Highlights

• HNC incidence is increasing globally, while death and DALYs are decreasing
• There has been a higher rise in incidence among females when compared to males
• In low SDI regions, under-20s show increased HNC related deaths and DALYs
• The mortality due to thyroid cancer has significantly increased above 60 years

## Graphical abstract

**Figure:**
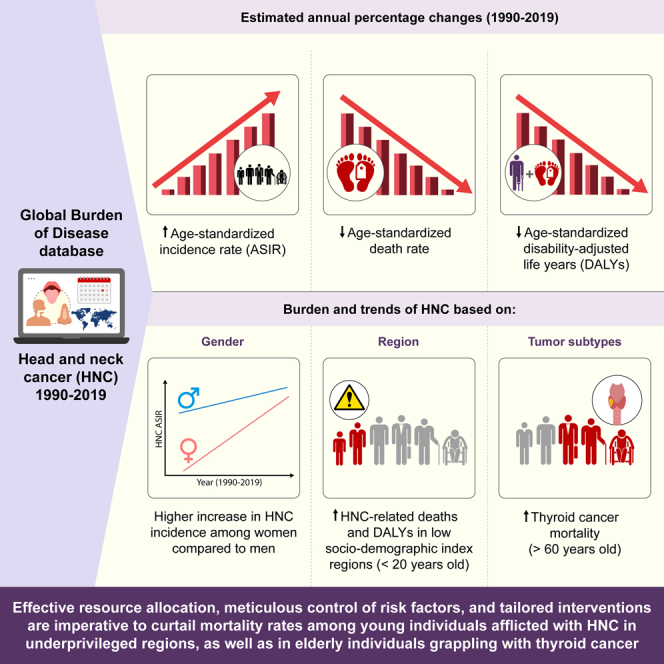

## Introduction

Head and neck cancer (HNC), anatomically defined, refers to a series of malignant tumors that occur above the clavicles, below the skull base, and in the anterior aspect of the neck vertebral column, including thyroid cancer (TC), lip-oral cavity cancer (LOC), larynx cancer (LC), nasopharynx cancer (NPC), and other pharynx cancer (OPC). These cancers pose significant challenges in otolaryngology-head and neck surgery. According to the Global Cancer Statistics of 2020, HNC ranked as the third most prevalent cancer worldwide, with 1,464,550 new cases and 487,993 deaths. This accounted for 7.6% of all cancers and 4.8% of all cancer-related deaths.^1^ HNC poses a profound impact on health worldwide. Patients with HNC often experience complex issues such as difficulties in swallowing, breathing, and communication, as well as psychosocial changes, leading to an increased burden on healthcare systems. Advances in surgical techniques, radiotherapy,^2^ and molecular targeted therapy^2^ have improved the early diagnosis and treatment strategies for HNC. However, previous studies on the burden of HNC with small sample sizes have been limited by sociological factors^3^ such as population structures,^3^ gender disparities, and regional economic variations.^4^ These limitations hinder a comprehensive understanding of the global disease burden.

To address this gap, the Global Burden of Diseases (GBD) database provides a valuable resource for assessing the global burden of diseases and injuries. The database utilizes standardized methodologies, ensuring consistency and comparability in measuring disease burden across populations and over time. It may be necessary to obtain more comprehensive global statistics to further verify the global burden of HNC. In an era where the global situation of HNC remains severe,^5^ it is crucial for governments and policymakers to increase awareness about prevention and allocate medical resources judiciously in order to reduce disease incidence and improve prognosis. This study utilized the latest GBD data from 1990 to 2019 to assess the trends in disease burden of HNC. The findings provide insights for tailored approaches to alleviate the burden of HNC globally.

## Results

### Global burden trend

**Figure 1:**
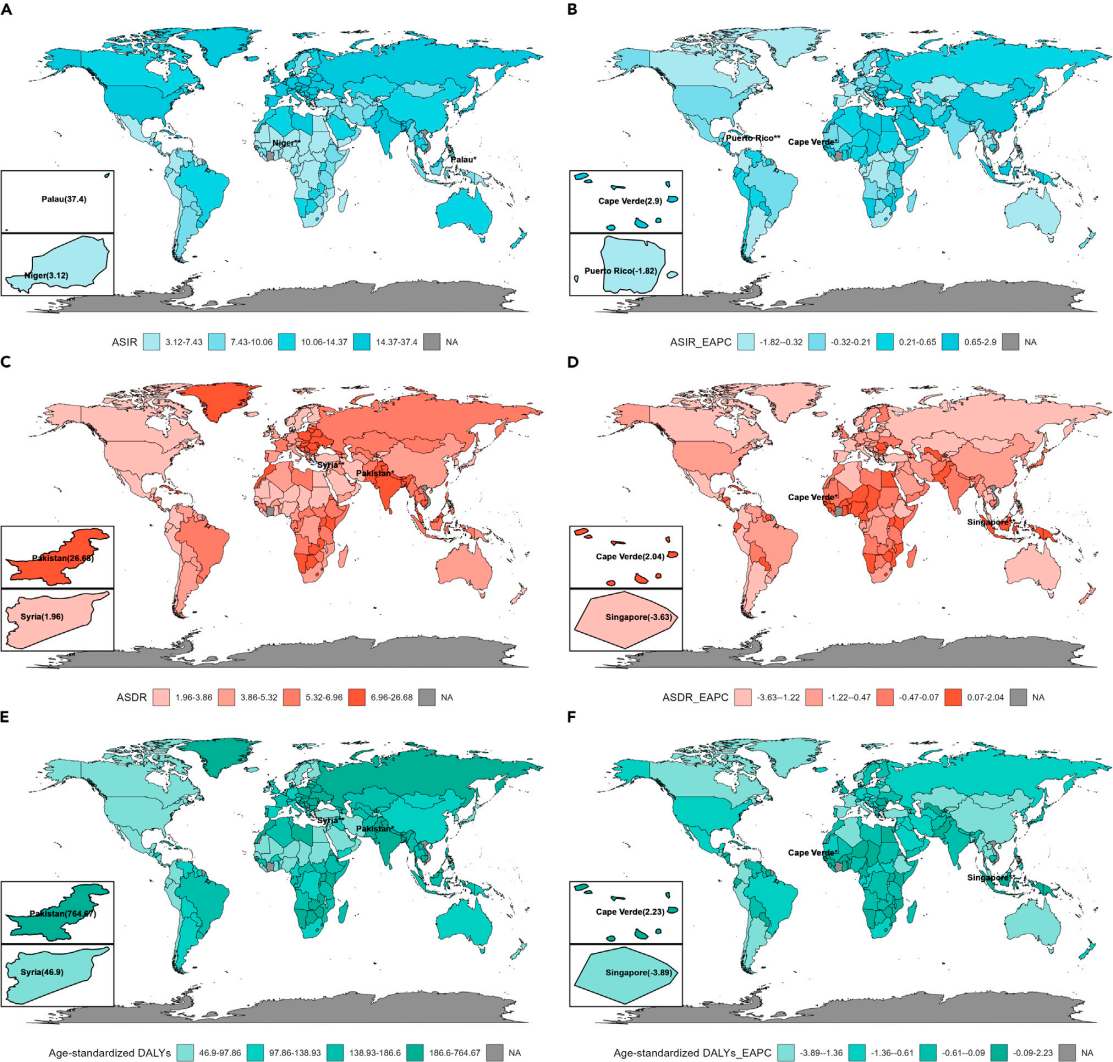
Age-standardized disease burden and estimated annual percentage change of head and neck cancer across 204 countries and territories from 1990 to 2019 (A) ASIR of head and neck cancer across 204 countries and territories from 1990 to 2019. (B) EAPC in ASIR of head and neck cancer across 204 countries and territories from 1990 to 2019. (C) ASDR of head and neck cancer across 204 countries and territories from 1990 to 2019. (D) EAPC in ASDR of head and neck cancer across 204 countries and territories from 1990 to 2019. (E) Age-standardized DALYs of head and neck cancer across 204 countries and territories from 1990 to 2019. (F) EAPC in Age-standardized DALYs of head and neck cancer across 204 countries and territories from 1990 to 2019. ASIR, age-standardized incidence rate; ASDR, age-standardized death rate; DALYs, disability-adjusted life years; EAPC, estimated annual percentage change.

Table 1 presents the incidence, death, disability-adjusted life years (DALYs), age-standardized incidence rate (ASIR), age-standardized death rate (ASDR), age-standardized DALYs, and estimated annual percentage change (EAPC) of HNC worldwide from 1990 to 2019. In 2019, the global incidence of HNC was 1,159,496 (95% uncertainty interval (UI): 1055403–1261568), with an ASIR 13.97 (95% UI: 12.71–15.19). The number of deaths was 554,146 (95% UI: 506,478–603,296), resulting in an ASDR of 6.74 (95% UI: 6.16–7.33). The number of DALYs was 15,570,403 (95% UI: 14,197,280-16,980,334), and age-standardized DALYs was 186.27 (95% UI: 169.9–203.07). Upon analyzing the age-standardized trends, it is evident that the ASIR has exhibited a progressive rise, with EAPC of 0.35 (95% confidence interval (CI): 0.31–0.39). Conversely, the ASDR and age-standardized DALYs have demonstrated a declining trend, with EAPC of −0.58 (95% CI: [-0.62]-[-0.53]) and −0.69 (95% CI: [-0.74]-[-0.64]), respectively. Among the 21 GBD regions, East Asia had the most rapid annual increase in ASIR, with an EAPC value of 2 (95% CI: 1.78–2.21). The region displaying the highest annual increase in ASDR was Western Sub-Saharan Africa, with an EAPC of 0.26 (95% CI: 0.19–0.33). Oceania exhibited the greatest annual increase in age-standardized DALYs, with an EAPC of 0.13 (95% CI: 0.09–0.18). The regions with the greatest decrease in EAPC for ASIR and age-standardized DALYs were both located in Southern Latin America, with rates of −0.74 (95% CI: [-0.83]-[-0.65]) and −1.97(95% CI: [-2.06]-[-1.87]), respectively. Table S1 and Figure 1 depicts the global distribution of ASIR, ASDR, age-standardized DALYs and their corresponding EAPC for the 204 countries. It is notable that Singapore had the lowest EAPC for ASDR of −3.63 (95% CI: [-3.78]-[-3.47]), and for age-standardized DALYs of −3.89 (95% CI: [-4.06]-[-3.72]).

**Table 1:** Global Burden of Head and Neck Cancer and Trends from 1990 to 2019 by 21 GBD regions,5 SDI regions, 4 World bank regions and Gender

Characteristics	1990		2019			1990		2019			1990		2019		
	Incidence cases	ASIR	Incidence cases	ASIR	EAPC	Death cases	ASDR	Death cases	ASDR	EAPC	DALYs cases	Age_standardised DALYs	DALYs cases	Age_standardised DALYs	EAPC
	(95%UI)	pre 100,000(95%UI)	(95%UI)	pre 100,000(95%UI)	(95%CI)	(95%UI)	pre 100,000(95%UI)	(95%UI)	pre 100,000(95%UI)	(95%CI)	(95%UI)	pre 100,000(95%UI)	(95%UI)	pre 100,000(95%UI)	(95%CI)
Global	521901(493850–551591)	12.49(11.83–13.19)	1159496(1055403–1261568)	13.97(12.71–15.19)	0.35(0.31–0.39)	311971(292176–334191)	7.73(7.24–8.27)	554146(506478–603296)	6.74(6.16–7.33)	−0.58(-0.62 to-0.53)	9432150(8807613–10134533)	219.64(205.38–235.61)	15570403(14197280–16980334)	186.27(169.9–203.07)	−0.69(-0.74 to-0.64)

**Gender**															

*Female*	173517(158742–187111)	7.85(7.2–8.45)	403042(359283–446574)	9.42(8.4–10.45)	0.58(0.54–0.62)	87159(78156–96235)	4.08(3.67–4.5)	159155(141359–177939)	3.67(3.26–4.1)	−0.5(-0.56 to-0.44)	2595725(2295175–2882912)	115.93(102.88–128.5)	4315090(3820765–4839253)	100.85(89.24–113.11)	−0.65(-0.74 to-0.57)
*Male*	348384(326867–372907)	17.81(16.72–19.03)	756454(677568–837379)	19(17.05–21)	0.2(0.13–0.27)	224812(207543–243444)	11.97(11.07–12.93)	394992(356193–435829)	10.18(9.19–11.23)	−0.64(-0.69 to-0.6)	6836424(6295419–7433863)	331.51(305.73–359.66)	11255313(10108979–12451677)	277.41(249.42–306.6)	−0.72(-0.76 to-0.67)

**SDI**															

*High SDI*	154296(149961–158041)	15.57(15.14–15.95)	257625(232330–284896)	15.83(14.27–17.52)	0.04(-0.04-0.13)	56319(54500–57583)	5.56(5.38–5.68)	75336(69347–79670)	4.12(3.83–4.35)	−1.12(-1.15 to-1.08)	1516824(1476783–1556272)	154.78(150.74–158.81)	1788444(1675408–1897379)	109.47(102.76–116.39)	−1.3(-1.33 to-1.27)
*High-middle SDI*	139384(132264–146095)	12.61(11.97–13.2)	287612(255650–322094)	14.57(12.95–16.31)	0.43(0.37–0.49)	83007(78772–87165)	7.69(7.3–8.07)	112608(102930–122147)	5.55(5.07–6.02)	−1.38(-1.47 to-1.28)	2470410(2333384–2610477)	220.91(208.73–233.36)	3085350(2814946–3358191)	154.36(140.76–168.12)	−1.54(-1.65 to-1.44)
*Middle SDI*	104299(95425–113658)	9.16(8.42–10.01)	315036(276831–354533)	12.14(10.7–13.62)	1.04(0.92–1.16)	74297(68227–81065)	7.06(6.49–7.71)	155350(138039–173382)	6.28(5.59–7)	−0.4(-0.45 to-0.36)	2339274(2145546–2550024)	194.49(178.63–212.29)	4369968(3876244–4891440)	165.3(146.87–184.8)	−0.57(-0.63 to-0.52)
*Low-middle SDI*	93525(82063–107551)	14.18(12.46–16.3)	228013(199114–257986)	15.7(13.75–17.72)	0.28(0.19–0.36)	74178(65215–85469)	11.98(10.51–13.79)	160476(141276–182947)	11.6(10.22–13.2)	−0.18(-0.25 to-0.11)	2331314(2049130–2682772)	331.29(291.3–382.04)	4749799(4176967–5425327)	316.55(278.47–361.43)	−0.22(-0.29 to-0.14)
*Low SDI*	30144(24973–35934)	11.33(9.44–13.44)	70672(61177–80681)	12.01(10.45–13.63)	0.13(0.09–0.17)	24021(20122–28404)	9.69(8.12–11.41)	50103(43698–57019)	9.35(8.18–10.6)	−0.2(-0.25 to-0.15)	770210(642822–914292)	270.79(226.82–320.47)	1569576(1365016–1800220)	253.15(220.62–288.41)	−0.31(-0.37 to-0.26)

**World Bank regions**															

*High Income*	196905(191660–201357)	16.19(15.77–16.56)	312211(281650–345111)	16.09(14.53–17.81)	−0.07(-0.15-0.01)	75522(73183–77060)	6.07(5.88–6.19)	95679(88331–100981)	4.39(4.1–4.63)	−1.22(-1.25to-1.19)	2035418(1984880–2083284)	169.22(165.06–173.22)	2277070(2136602–2410397)	117.13(110.24–124.14)	−1.4(-1.44to-1.36)
*Upper Middle Income*	146622(135206–158269)	8.97(8.29–9.65)	402637(353386–458987)	11.89(10.46–13.54)	1.03(0.91–1.16)	100588(92473–108843)	6.53(6.02–7.06)	161077(143258–178454)	4.8(4.28–5.31)	−1.16(-1.21to-1.1)	3108521(2844779–3371517)	183.31(168.27–198.57)	4391650(3908341–4885553)	127.62(113.7–141.75)	−1.41(-1.47to-1.34)
*Lower Middle Income*	164528(147551–184033)	14.34(12.86–16.05)	415814(359790–470352)	16.31(14.16–18.42)	0.35(0.29–0.41)	125572(112510–141232)	11.65(10.38–13.11)	278044(243944–316199)	11.59(10.18–13.15)	−0.13(-0.19to-0.07)	3954340(3547974–4447031)	325.05(291.44–365.26)	8292354(7267223–9431521)	313.8(275.29–356.6)	−0.21(-0.27to-0.15)
*Low Income*	13591(11076–16788)	7.93(6.58–9.65)	28295(23205–33930)	7.78(6.42–9.23)	−0.11(-0.19to-0.02)	10139(8444–12354)	6.43(5.41–7.73)	19073(15785–22669)	5.87(4.88–6.92)	−0.33(-0.39to-0.28)	329735(269413–406790)	183.26(151.91–224.04)	602041(493467–722718)	158.74(130.94–189.56)	−0.55(-0.61to-0.48)

**GBD regions**															

*Andean Latin America*	1125(987–1300)	5.07(4.46–5.83)	3759(2961–4661)	6.49(5.11–8.04)	1(0.91–1.08)	777(687–885)	3.84(3.38–4.39)	1777(1413–2160)	3.22(2.56–3.91)	−0.5(-0.61 to-0.39)	21521(18988–24624)	94.73(83.51–108.06)	43061(34100–53040)	74.99(59.47–92.11)	−0.75(-0.85 to-0.64)
*Australasia*	3351(3162–3554)	14.65(13.82–15.55)	5464(4337–6856)	12.66(10–15.92)	−0.43(-0.54 to-0.32)	1610(1535–1682)	6.99(6.65–7.3)	2045(1828–2233)	4.23(3.79–4.6)	−1.78(-1.87 to-1.7)	41577(39752–43556)	183.17(175.08–191.96)	47079(42545–51451)	107.61(97.66–117.58)	−1.85(-1.93 to-1.76)
*Caribbean*	3093(2896–3299)	11.66(10.91–12.43)	6732(5695– 7921)	12.99(10.99–15.29)	0.53(0.39–0.67)	2070(1926–2223)	8.06(7.51–8.65)	3954(3374–4590)	7.62(6.5–8.84)	−0.05(-0.2-0.1)	53235(49221–57676)	198.6(183.92–215.03)	98891(83635–115860)	190.47(161.13–222.98)	0.01(-0.14-0.16)
*Central Asia*	4544(4234–4989)	9.03(8.41–9.95)	6885(6183–7705)	8.47(7.62–9.45)	−0.37(-0.49 to-0.25)	3114(2912–3400)	6.4(5.98–7.02)	4023(3617–4494)	5.31(4.79–5.91)	−0.82(-0.93 to-0.71)	95633(89399–103799)	185.63(173.62–202.04)	122164(109372–137208)	144.25(129.58–161.28)	−1.12(-1.24 to-1)
*Central Europe*	21900(21047–22535)	14.97(14.38–15.41)	32096(27858–36571)	17.06(14.79–19.49)	0.38(0.27–0.48)	13419(12981–13753)	9.18(8.87–9.41)	16454(14318–18695)	8.29(7.2–9.43)	−0.42(-0.53 to-0.31)	395376(381890–405836)	270.63(261.41–277.85)	440734(381087–502305)	238.08(205.26–271.74)	−0.57(-0.7 to-0.45)
*Central Latin America*	5860(5606–6060)	6.63(6.31–6.87)	16513(14013–19346)	6.85(5.82–8.02)	−0.12(-0.21 to-0.03)	3876(3687–4012)	4.83(4.56–5.02)	8239(7025–9607)	3.55(3.03–4.13)	−1.32(-1.42 to-1.23)	102975(98908–106346)	113.36(108.55–117.22)	201005(170658–236805)	83.4(70.89–98.06)	−1.31(-1.41 to-1.21)
*Central Sub-Saharan Africa*	1529(1178–1931)	6.16(4.74–7.72)	3407(2554–4413)	5.78(4.33–7.47)	−0.28(-0.39 to-0.18)	1254(981–1561)	5.46(4.27–6.74)	2592(1955–3350)	4.82(3.62–6.22)	−0.48(-0.56 to-0.4)	38987(30422–48899)	144.21(112.75–179.77)	80510(60628–104142)	125.04(94.27–161.75)	−0.55(-0.64 to-0.47)
*East Asia*	77105(66595–87835)	7.97(6.93–9.05)	266166(220629–317916)	12.97(10.82–15.44)	2(1.78–2.21)	54393(46994–62145)	6.07(5.28–6.9)	90453(76077–106520)	4.41(3.73–5.17)	−1.01(-1.09 to-0.93)	1755791(1509259–2009027)	172.75(148.96–196.98)	2502094(2109594–2950612)	119.16(100.85–139.75)	−1.27(-1.35 to-1.19)
*Eastern Europe*	34762(32954–37082)	12.41(11.77–13.25)	48667(42532–55558)	15.38(13.43–17.6)	0.45(0.24–0.65)	22381(21311–23615)	7.94(7.56–8.38)	23800(20807–26943)	7.13(6.24–8.07)	−0.97(-1.24 to-0.7)	671965(638330–712049)	239.72(227.54–254.24)	681973(595946–774048)	213.9(187.1–242.78)	−1.02(-1.31 to-0.74)
*Eastern Sub-Saharan Africa*	7840(6239–9800)	8.69(7.06–10.68)	17589(14131–21421)	8.69(7.07–10.42)	−0.09(-0.14 to-0.03)	5911(4811–7251)	7.18(5.91–8.69)	11649(9478–14027)	6.59(5.39–7.87)	−0.35(-0.38 to-0.32)	199447(159410–248572)	210.49(170.97–259.28)	387997(312704–471587)	184.59(149.97–222.71)	−0.54(-0.57 to-0.5)
*High-income Asia Pacific*	19054(18077–20434)	9.37(8.89–10.06)	44057(37327–51063)	12.2(10.4–14.16)	1.16(0.74–1.59)	6683(6371–7025)	3.4(3.22–3.57)	14610(12509–15918)	3.11(2.76–3.36)	−0.43(-0.64 to-0.23)	172921(165496–182418)	84.55(80.81–89.23)	279175(251731–302271)	73.82(67.63–79.8)	−0.59(-0.84 to-0.35)
*High-income North America*	58901(57053–60496)	17.91(17.37–18.39)	97978(83971–114456)	17.23(14.73–20.15)	−0.25(-0.31 to-0.19)	16206(15591–16638)	4.74(4.58–4.87)	21870(20565–22830)	3.51(3.32–3.66)	−1.15(-1.26 to-1.05)	419681(405627–433195)	129.46(125.24–133.67)	529804(502099–556465)	91.96(87.24–96.55)	−1.31(-1.41 to-1.2)
*North Africa and Middle East*	14065(11583–15911)	7.17(5.9–8.14)	45654(39031–52472)	9.11(7.9–10.39)	0.84(0.76–0.93)	9020(7350–10305)	5.15(4.19–5.9)	17825(15758–20354)	4.14(3.68–4.74)	−0.78(-0.84 to-0.72)	279475(230011–317546)	138.83(113.35–158.53)	527048(458326–606549)	107.21(94.18–122.7)	−0.96(-1.02 to-0.9)
*Oceania*	235(185–302)	6.92(5.48–8.81)	608(454–813)	7.5(5.72–9.79)	0.32(0.29–0.35)	154(121–198)	5.14(4.06–6.55)	372(281–496)	5.24(4.06–6.81)	0.16(0.11–0.21)	4972(3874–6435)	137.05(107.29–176.11)	11942(8865–16229)	138.54(104.61–185.01)	0.13(0.09–0.18)
*South Asia*	120316(105824–138691)	19.25(16.85–22.24)	307993(261592–357262)	20.58(17.51–23.8)	0.11(0.03–0.19)	95852(83858–110481)	16.46(14.35–19)	219362(188945–254824)	15.41(13.3–17.86)	−0.36(-0.44 to-0.28)	3035861(2675542–3500758)	448.03(392.85–516.09)	6556721(5648782–7620555)	422.69(364.17–490.95)	−0.3(-0.37 to-0.22)
*Southeast Asia*	31350(27260–34887)	11(9.63–12.18)	84994(70837–100093)	13.1(10.98–15.41)	0.49(0.43–0.56)	20787(18363–22955)	8.02(7.1–8.84)	45014(38510–52435)	7.5(6.43–8.73)	−0.33(-0.37 to-0.28)	644302(565321–713595)	215.2(190.04–238.06)	1280774(1082640–1500122)	192.53(163.89–224.97)	−0.49(-0.54 to-0.43)
*Southern Latin America*	4743(4491–4989)	10.16(9.63–10.68)	7026(5491–8902)	8.73(6.81–11.08)	−0.74(-0.83 to-0.65)	3043(2900–3180)	6.61(6.29–6.91)	3546(3290–3811)	4.28(3.97–4.59)	−1.67(-1.75 to-1.59)	83136(78968–87057)	177.5(168.6–185.83)	86235(80040–93292)	107.15(99.49–116)	−1.97(-2.06 to-1.87)
*Southern Sub-Saharan Africa*	2516(2243–2880)	8.39(7.43–9.66)	4727(4254–5286)	7.83(7.07–8.71)	−0.46(-0.76 to-0.16)	1782(1586–2050)	6.33(5.59–7.31)	3229(2926–3579)	5.67(5.16–6.26)	−0.58(-0.95 to-0.21)	54795(49294–62501)	175.73(157.19–202.01)	94547(85000–105682)	151.15(136.34–168.32)	−0.72(-1.1 to-0.33)
*Tropical Latin America*	11040(10637–11479)	11.27(10.84–11.72)	27510(25860–29258)	11.08(10.41–11.79)	−0.07(-0.23-0.08)	7763(7468–8064)	8.42(8.04–8.76)	16887(15798–17898)	6.9(6.44–7.32)	−0.68(-0.8 to-0.57)	229498(221349–238259)	225.41(217.17–233.93)	461331(435081–490123)	183.68(173.06–195.1)	−0.75(-0.89 to-0.6)
*Western Europe*	94943(91616–97819)	18.28(17.62–18.84)	122991(106043–142409)	16.5(14.18–19.14)	−0.48(-0.55 to-0.42)	38833(37574–39797)	7.13(6.91–7.31)	39719(36705–41914)	4.66(4.36–4.91)	−1.59(-1.65 to-1.54)	1039258(1008882–1068291)	202.71(196.65–208.48)	932961(875311–988952)	124.77(117.52–132.45)	−1.85(-1.91 to-1.8)
*Western Sub-Saharan Africa*	3629(2967–4320)	3.85(3.17–4.55)	8680(7110–10393)	4.19(3.49–4.94)	0.41(0.35–0.47)	3043(2507–3648)	3.42(2.83–4.06)	6723(5499–8027)	3.54(2.95–4.18)	0.26(0.19–0.33)	91746(75132–110661)	91.24(74.95–109.91)	204356(163753–247274)	90.41(73.55–108.24)	0.06(0–0.12)

### Burden trends by socio-demographic index (SDI)

**Figure 2:**
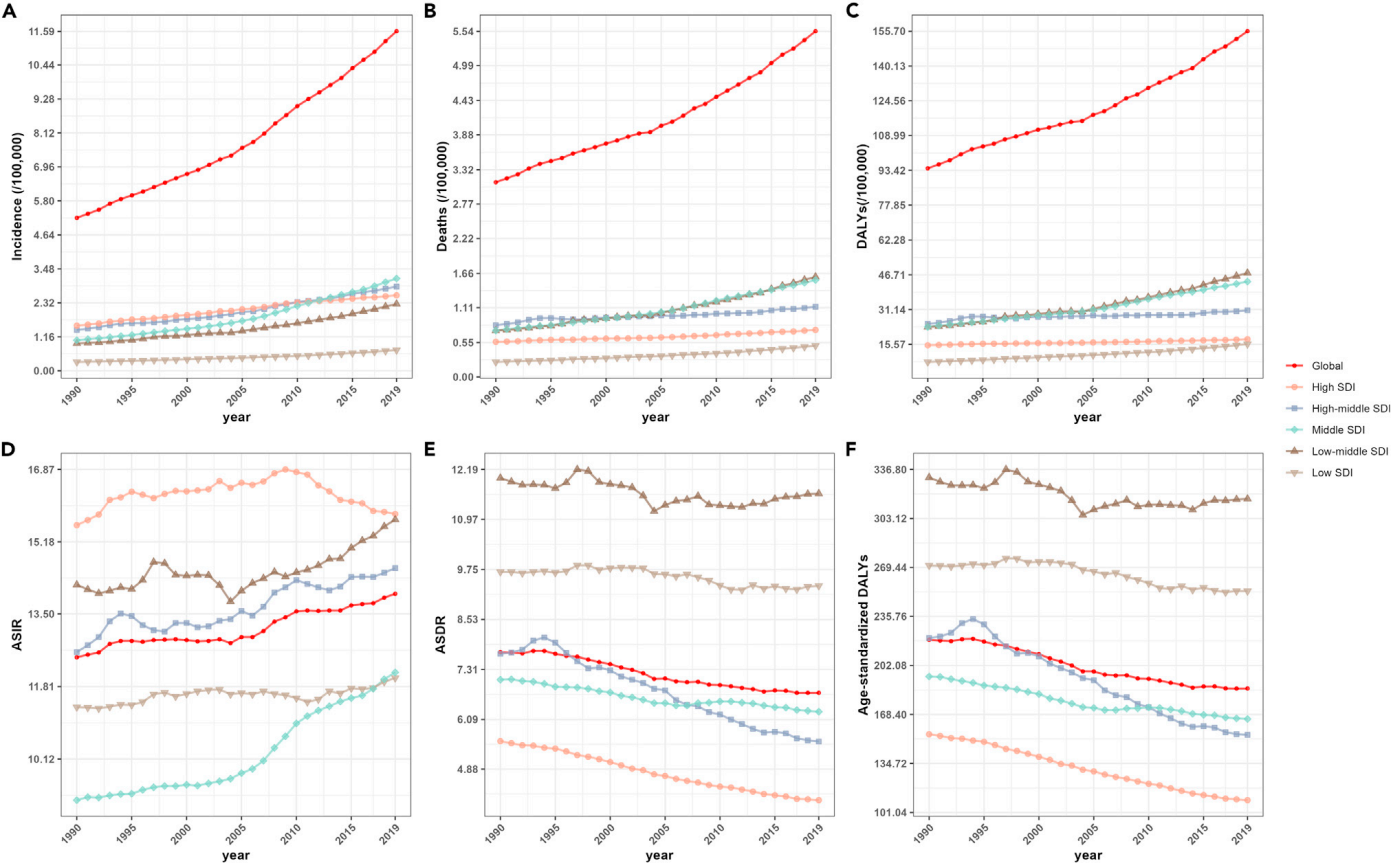
Trends in the disease burden of head and neck cancer from 1990 to 2019 by different SDI level regions (A) Trends in incidence of head and neck cancer from 1990 to 2019 by different SDI level regions. (B) Trends in deaths of head and neck cancer from 1990 to 2019 by different SDI level regions. (C) Trends in DALYs of head and neck cancer from 1990 to 2019 by different SDI level regions. (D) Trends in ASIR of head and neck cancer from 1990 to 2019 by different SDI level regions. (E) Trends in ASDR of head and neck cancer from 1990 to 2019 by different SDI level regions. (F) Trends in age-standardized DALYs of head and neck cancer from 1990 to 2019 by different SDI level regions. ASIR, age-standardized incidence rate; ASDR, age-standardized death rate; DALYs, disability-adjusted life years; SDI, socio-demographic index.

**Figure 3:**
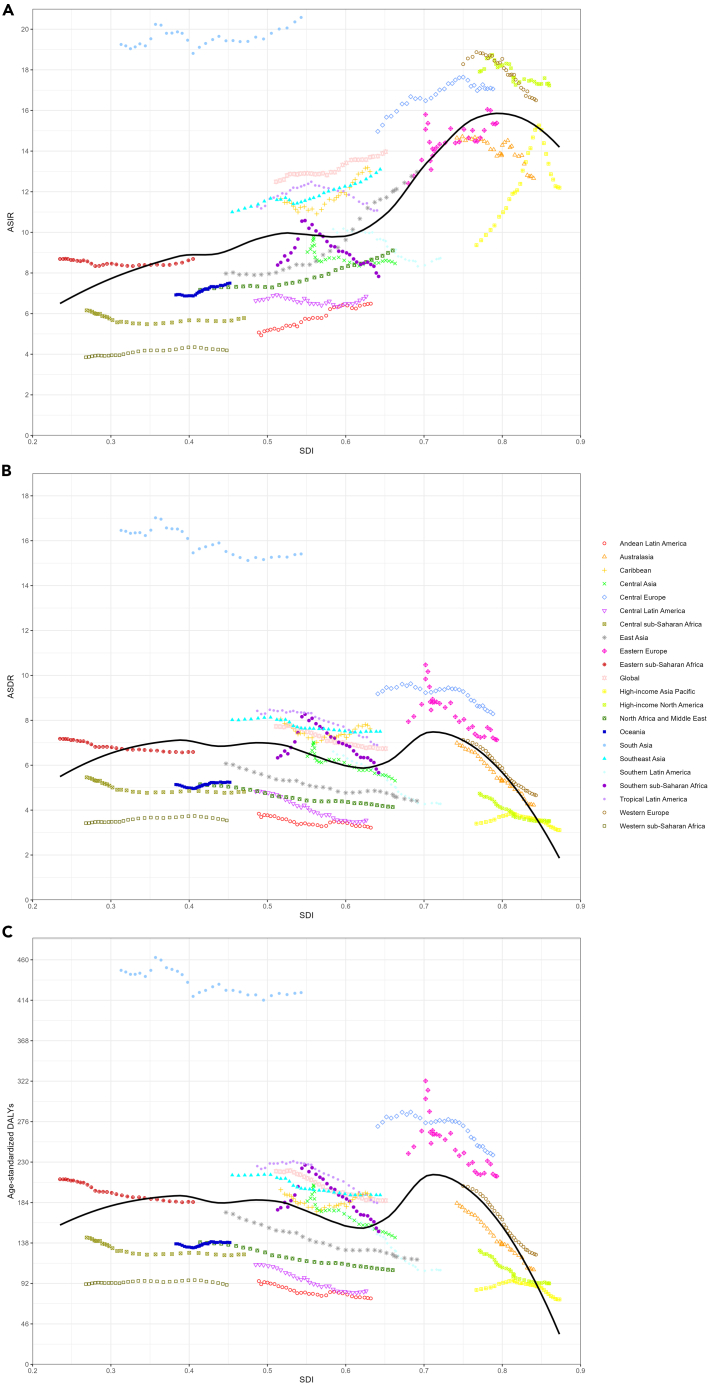
Trends in disease burden of head and neck cancer across Global and 21 GBD regions by SDI from 1990 to 2019 (A) Trends in ASIR of head and neck cancer across Global and 21 GBD regions by SDI from 1990 to 2019. (B) Trends in ASDR of head and neck cancer across Global and 21 GBD regions by SDI from 1990 to 2019. (C) Trends in age-standardized DALYs of head and neck cancer across Global and 21 GBD regions by SDI from 1990 to 2019. ASIR, age-standardized incidence rate; ASDR, age-standardized death rate; DALYs, disability-adjusted life years; SDI, socio-demographic index.

In 2019, compared to 1990, there was a general decrease in ASDR and age-standardized DALYs across all SDI regions, while incidence, death, DALYs, and ASIR exhibited an increase trend. There was no evident correlation between SDI levels and incidence, death, DALYs, ASIR, and ASDR, as well as age-standardized DALYs (Figures S1A‒S1C). Interestingly, in 2019, the high SDI level region exhibited the highest ASIR for HNC, with a value of 15.83 (95% UI: 14.27–17.52). However, it also displayed the lowest ASDR 4.12 (95% UI: 3.83–4.35) as well as the lowest age-standardized DALYs 109.47 (95% UI: 102.76–116.39) (Figures S1D‒S1F). Based on the data from 1990 to 2019, a comprehensive analysis of different SDI regional data reveals a slowly increasing trend in the incidence, mortality, and DALYs (Figures 2A–2C). Conversely, there is a decreasing trend in ASDR and age-standardized DALYs, except in the low-medium or low SDI regions with stable trend (Figures 2D–2F). Furthermore, significant variations in ASIR are observed across different SDI-level regions. The trends in ASIR, ASDR, and age-standardized DALYs in relation to SDI from 1990 to 2019 for both the global and the 21 GBD regions are shown in Figure 3. Based on the fitted curves, the relationship between age-standardized disease burden indicators and SDI varies across regions. The findings reveal a noteworthy observation regarding ASIR, ASDR, and age-standardized DALYs in South Asia, which exhibits a substantial high level compared to other regions.

### Burden trends by gender

Table 1 showed the EAPC of ASIR in females was 0.58 (95% CI: 0.54–0.62) more than the content in males of 0.2 (95% CI: 0.13–0.27). Simultaneously, the EAPC of ASDR was −0.5 (95% CI: [-0.56]-[-0.44]) in females and −0.64 (95% CI: [-0.69]-[-0.6]) in males. Figure S2 showed different disease burden indicators for HNC from 1990 to 2019 by gender and male to female ratio. However, male patients exhibited higher values in incidence, death, DALY, ASIR, ASDR, and age-standardized DALYs compared to female patients. Importantly, there was a notable decrease in the ratio of male to female of HNC incidence cases from 2.01 in 1990 to 1.88 in 2019. Similarly, the gender ratio of ASIR decreased from 2.27 to 2.02 during the same period.

### Burden trends by gender and SDI

**Figure 4:**
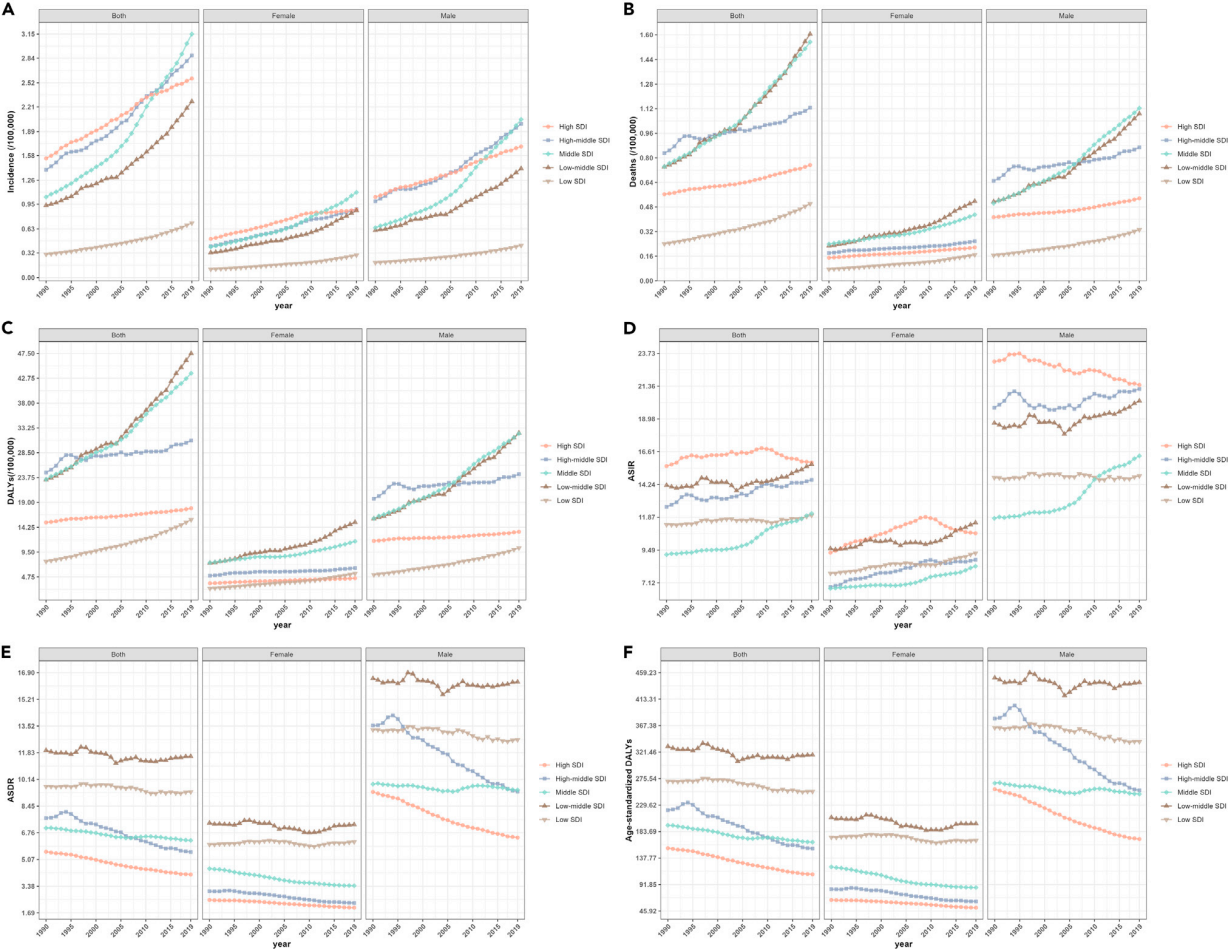
Trends in disease burden of head and neck cancer across SDI level regions by gender from 1990 to 2019 (A) The trends in incidence of head and neck cancer across SDI level regions by gender from 1990 to 2019. (B) The trends in deaths of head and neck cancer across SDI level regions by gender from 1990 to 2019. (C) The trends in DALYs of head and neck cancer across SDI level regions by gender from 1990 to 2019. (D) The trends in ASIR of head and neck cancer across SDI level regions by gender from 1990 to 2019. (E) The trends in ASDR of head and neck cancer across SDI level regions by gender from 1990 to 2019. (F) The trends in age-standardized DALYs of head and neck cancer across SDI level regions by gender from 1990 to 2019. ASIR, age-standardized incidence rate; ASDR, age-standardized death rate; DALYs, disability-adjusted life years; SDI, socio-demographic index.

From 1990 to 2019, the trends of incidence, death, and DALYs for HNCs were generally consistent across genders in SDI regions, with all showing an increasing trend over the years (Figures 4A–4C). However, there were gender differences observed in the values of ASIR, ASDR, and age-standardized DALYs. In most SDI regions, both males and females showed an increasing trend in ASIR. Specifically, there was a notable increase in ASIR among female in low SDI region, while the ASIR among male remained stable (Figure 4D). Additionally, in both low and low-middle SDI regions, there were no significant changes observed in ASDR and age-standardized DALYs by gender (Figures 4E and 4F). However, in high-middle SDI regions, males experienced the most significant decrease in ASDR and age-standardized DALYs compared to other SDI regions (Figures 4E and 4F).

### Burden trends by age and SDI

The trends of incidence, deaths, DALYs for HNC across different age groups (5-year intervals) in different SDI regions from 1990 to 2019 were showed in Figure S3. Based on age groups, significant differences in disease burden indicators between different SDI levels are observed. In 2019, compared to 1990, there was an overall increasing trend in the incidence, deaths, and DALYs for HNC with increasing age, except for a decrease in deaths and DALYs among individuals aged under 20 (Figure S4). In the under-20 age group, there was a notable decline in the incidence of HNC in high-middle and high SDI regions compared to other regions, with values of −0.08 and −0.01 respectively. While in low SDI regions, the number of deaths and DALYs due to HNC in individuals aged under 20 showed an increase. In the 20–49 age group, the incidence of HNC in high-middle SDI regions (1.32) was lower than in low (1.59) or middle-low (1.46) SDI regions. However, there was a notable decrease in the rate of increase for deaths and DALYs in high-middle regions (0.04 and 0.06 for the rate of deaths and DALYs, respectively) compared to low (1.11 and 1.12 for the rate of deaths and DALYs, respectively) and middle-low (0.96 and 0.96 for the rate of deaths and DALYs, respectively) SDI regions. Similar trends were observed in the 50–69 age group as well. In the over-70 age group, there is a gradual decrease in the rate of increase for incidence, deaths, and DALYs as the SDI level increases.

### Burden trends by age and gender

**Figure 5:**
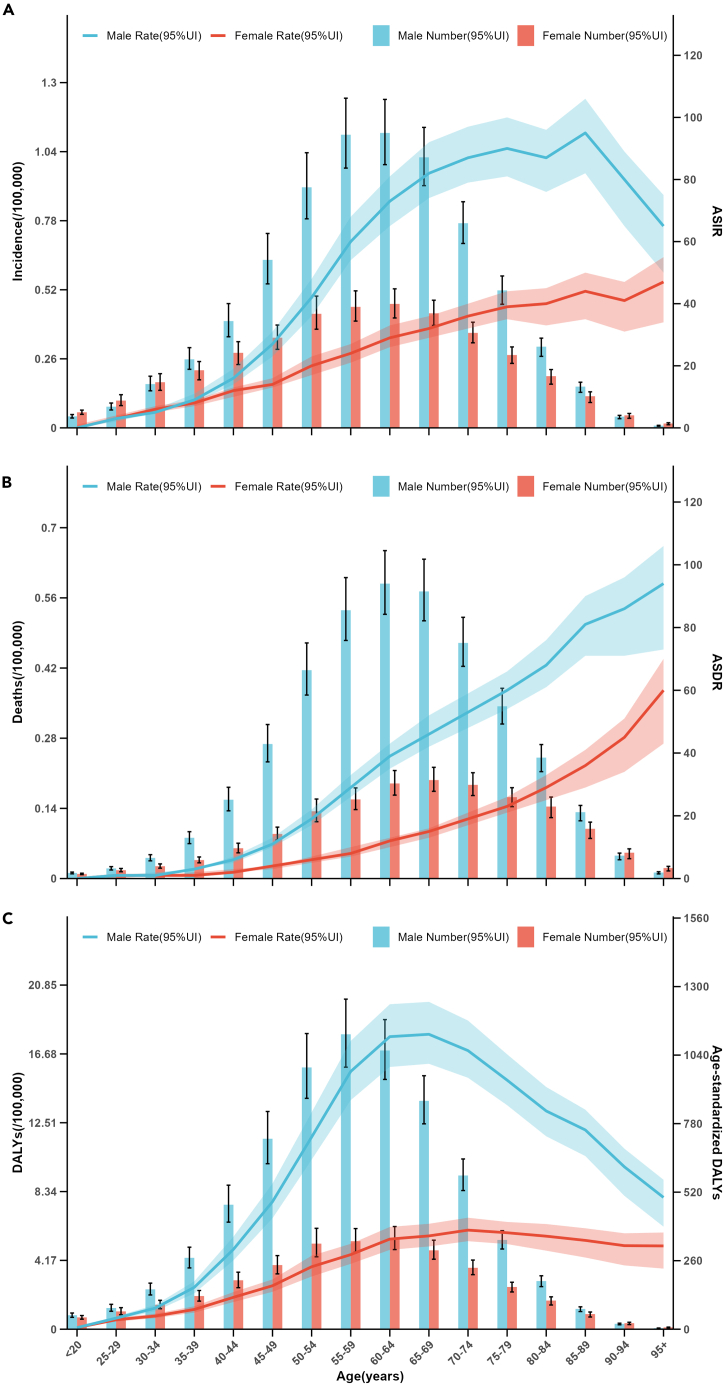
Trends in disease burden of head and neck cancer across different age groups (5-year intervals) by gender in 2019 (A) Trends in incidence and ASIR of head and neck cancer across different age groups (5-year intervals) by gender in 2019. (B) Trends in deaths and ASDR of head and neck cancer across different age groups (5-year intervals) by gender in 2019. (C) Trends in DALYs and age-standardized DALYs of head and neck cancer across different age groups (5-year intervals) by gender in 2019. ASIR, age-standardized incidence rate; ASDR, age-standardized death rate; DALYs, disability-adjusted life years. Data are represented as mean ± SEM.

The trends of incidence, deaths, DALYs, ASIR, ASDR, age-standardized DALYs for HNC across different age groups by gender were showed in Figure 5. In 2019, a notable transition occurred in HNC incidence, with males of 16,487 (95% UI: 13,956-19,450) surpassing females 17,190 (95% UI: 14,135–20,403) from age 35 (Figure 5A). Although the incidence cases of HNC in males is lower than in females under age 35, it is important to highlight that the number of death cases is higher in males of 241,585 (95% UI: 207,546–279,188) than female of 148,189 (95% UI: 124,242-175,146) (Figure 5B). In addition, males exhibit a significant decline in age-standardized DALYs after the 70–74 age group, whereas females’ trends tend to stabilize.

### Subtypes of HNC

**Figure 6:**
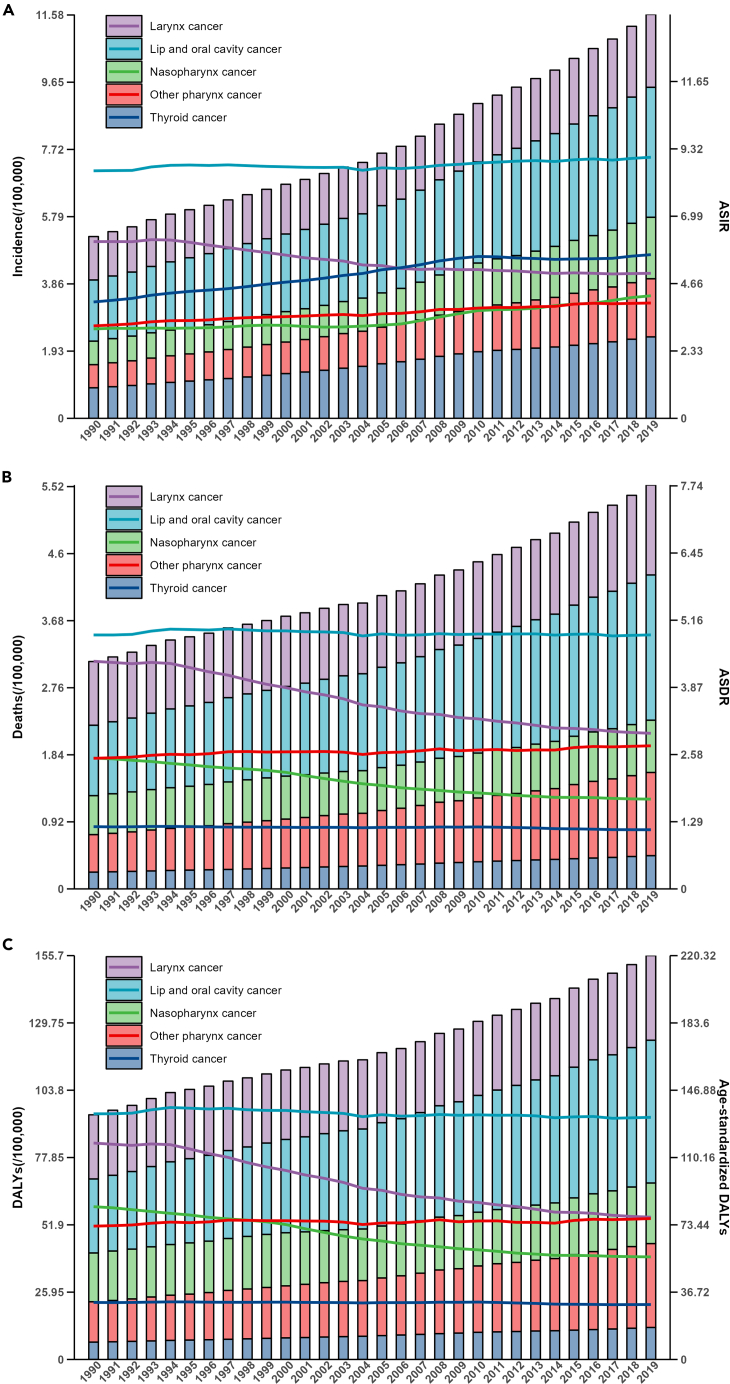
Trends in disease burden of head and neck cancer subtypes from 1990 to 2019 (A) Trends in incidence and ASIR of head and neck cancer across different age groups (5-year intervals) by gender in 2019. (B) Trends in deaths and ASDR of head and neck cancer across different age groups (5-year intervals) by gender in 2019. (C) Trends in DALYs and age-standardized DALYs of head and neck cancer across different age groups (5-year intervals) by gender in 2019. ASIR, age-standardized incidence rate; ASDR, age-standardized death rate; DALYs, disability-adjusted life years.

In 2019, LOC had the highest incidence (373,098, 95% UI: 340,884-403,866), deaths (199,398, 95% UI: 181,651–218,059), DALYs (5,506,652, 95% UI: 5,004,325–6,033,424), ASIR (4.52, 95% UI: 4.13–4.89), ASDR (2.44, 95% UI: 2.22–2.66), and age-standardized DALYs (66.05, 95% UI: 60.06–72.35) among 5 subtypes of HNC, respectively (Table S2). On the other hand, OPC had the lowest incidence and ASIR, while TC had the lowest deaths, DALY, ASDR, and age-standardized DALYs (Table S2). The trends of incidence, deaths, DALY, ASIR, ASDR, and age-standardized DALYs for 5 subtypes of HNC from 1990 to 2019 are presented in Figure 6. LC showed a decreasing trend in ASIR with EAPC of −0.86 (95% CI: [-0.95]-[-0.77]). In contrast, other HNC subtypes exhibited increasing trends. Among them, TC with the highest EAPC of 1.25 (95% CI: 1.12–1.37) in ASIR. Other pharyngeal cancer, as the exclusive HNC subtype demonstrated an increasing trend in ASDR with EAPC of 0.25 (95% CI: 0.21–0.29) and age-standardized DALYs with EAPC of 0.1 (95% CI: 0.05–0.14) (Table S2). Notably, while NPC exhibited an increasing trend in ASIR with EAPC value of 1.13 (95% CI: 0.97–1.29). However, it demonstrated decreasing trend in ASDR and age-standardized DALYs, with values of −1.48(95% CI: [-1.56]-[-1.39]) and −1.58(95% CI: [-1.67]-[-1.48]), respectively. The distribution of incidence, deaths, DALYs rates and the annual rate of change in the proportion (ARCP) of HNC subtypes between 1990 and 2019 in the proportions of five HNC subtypes, stratified by different SDI regions and genders was showed in Figure S5. In high-middle SDI regions, the male population exhibited the highest ARCP for NPC incidence, with a value of 3.42. Conversely, in low-middle and low SDI regions, the ARCP values for females were −1.48 and −1.42, respectively. In the case of LOC, both deaths and DALYs demonstrate an increase in the proportion rate (ARCP >0) across all regions and genders. Conversely, for LC and NPC, the ARCP for both deaths and DALYs consistently shows a decrease in the proportion rate (ARCP <0) across all regions and genders. The trend of ASIR with EAPC for female LC was −0.49 (95% CI: [-0.54]-[-0.43]), compared to −0.95 (95% CI: [-1.05]-[-0.86]) for males. Meanwhile, the trend of ASIR of EAPC for female LOC was 0.46 (95% CI: 0.4–0.52), whereas for males, it was −0.04 (95% CI: [-0.1]-0.01) (Table S2). The trend of ASDR with EAPC for female LC was −1.07 (95% CI: [-1.13]-[-1.01]), compared to −1.61 (95% CI: [-1.69]-[-1.54]) for males. Meanwhile, the trend of ASDR of EAPC for female LOC was 0.21 (95% CI: 0.15–0.28), whereas for males, it was −0.22 (95% CI: [-0.26]-[-0.19]) (Table S2). The trends of incidence, deaths, DALYs, ASIR, ASDR, age-standardized DALYs for LC, LOC, NPC, OPC, and TC across different age groups (5-years intervals) by gender from showed in Figures S6–S10. And significantly lower ASDR and age-standardized DALYs for TC were observed compared to other subtypes. Compared to other subtypes of HNC, females across all age groups exhibit a higher incidence, deaths, DALYs in TC. The mortality due to TC significantly increased in patients aged above 60 patients with the highest death occurs in the age group of 70–79 (Figure S10). Additionally, there is a significant increase in the ASDR for both males and females with TC after age 70.

## Discussion

This study is the first to utilize data from the GBD for 1990–2019 to comprehensively investigate the burden and trends of HNC based on region, gender, age, and subtypes, offering personalized insights for future public health decisions.

We found that the ASIR of HNC are increasing globally. On one hand, the rising incidence is associated with deteriorating environmental conditions and increased exposure to risk factors, such as alcohol consumption, smoking, and infections with Epstein-Barr virus (EBV) or human papillomavirus (HPV).^3^^,^^4^For example, the increase in risky behaviors, such as smoking and alcohol consumption, are potential factors in the rising incidence of HNC. Studies have shown that from 1990 to 2017, global adult per capita alcohol consumption increased from 5.9 L (95% CI: 5.8–6.1) to 6.5 L (95%CI: 6.6–6.9), and it is projected to reach 7.6 L (95% CI: 6.5–10.5) by 2030.^6^ The number of smokers increased from 900 million in 1990 to 1.14 billion in 2019.^7^ Second, the increasing number of HPV infections worldwide is a potential factor contributing to the rise in HNC incidence,^8^ similar to the trend observed in other non-HNCs such as cervical cancer^9^ and anal cancer.^10^ As the current global HPV vaccination capacity improves,^11^ it is essential to reduce the incidence of HNC by expanding HPV vaccination coverage.^12^ On the other hand, the implementation of endoscopic technologies and policies for early screening of HNC has significantly improved diagnostic efficiency for HNC. For example, image technology, especially endoscopic screening has enabled early detection, and curative treatment (80%) has been achieved through endoscopic resection.^13^ Narrow-band imaging diagnosis has shown accuracy rates of 86.7% and 88.9% for superficial cancers, significantly higher than those under white light.^14^ With the further development of physical and chemical screening technologies such as targeted topical fluorophores,^15^ whether ASIR will continue to rise requires further observation.

Although we observed an upward trend in ASIR of HNC, both ASDR and age-standardized DALYs have shown stable or declining patterns, consistent with previous study.^16^ We consider two potential reasons for this trend. First, the mortality of HNC is significantly correlated with tumor stage and recurrence.^4^ Early screening for HNC, as previously mentioned, and timely detection of recurrent lesions play a significant role in reducing mortality rates.^17^ Second, the widespread adoption of comprehensive treatment modalities, including surgery, radiotherapy, and chemotherapy, has led to lower mortality rates in HNC, especially for recurrent and metastatic cases. Between December 24, 2014, and May 13, 2016, in a clinical trial involving 247 patients with advanced head and neck tumors (Stage 3), pembrolizumab demonstrated a median overall survival of 8.4 months, surpassing conventional chemotherapy with 6.9 months.^18^ Similar improvements were observed with chemotherapy plus cetuximab.^19^ With the advancements in genetic diagnostics^20^ and targeted therapies, further reductions in ASDR or DALYs for HNC might be achieved.

The burden of HNC displays significant regional disparities. Our findings indicate that the annual age-standardized incidence rate of HNC experienced the most pronounced increase in East Asia, possibly linked to dietary and unhealthy lifestyle habits in the region. For example, alcohol exposure studies across 189 countries revealed a 104% rise in adult alcohol per-capita consumption in South-East Asia between 1990 and 2017.^6^ Conversely, among 204 countries, Singapore demonstrated the most substantial reduction in age-standardized death and disability rates for head and neck tumors, which may be attributed to the stringent anti-smoking policies enforced in the country for over two decades. Recent research further indicated that from 1990 to 2019, smoking prevalence among Singaporean women decreased at an annual change rate of −32.6% (95 CI%: [-52.1]-[-8.90]%), and among men at −31.3% (95 CI%: [-39.6]-[-22.5]%), representing the most rapid decline in smoking rates among high-income Asia-Pacific countries.^7^ Accordingly, we believe that implementing global policies, such as the World Health Organization Framework Convention on Tobacco Control, could prove beneficial in reducing the disease burden of HNC. Apart from regional lifestyle differences, the socioeconomic development level also influences the disease burden of HNC. Variations in HNC burden across different SDI regions reflect social disparities in prevention and healthcare resources for these cancers. We found that high SDI regions (e.g., high-income Asia Pacific and high-income North America) show higher incidence rates and lower mortality rates for HNC compared to low and middle-low SDI regions. Notably, low SDI regions (e.g., South Asia) consistently maintain the highest ASIR, ASDR, and age-standardized DALYs of HNC. In underdeveloped countries, limited medical facilities often result in delayed diagnosis of HNC with the majority of patients at later stages,^21^ leading to poorer quality of life. Therefore, healthcare resources should prioritize resource-constrained regions, including improving access to basic endoscopy and comprehensive treatment options.

Our study found that from 1990 to 2019, males consistently had significantly higher rates of HNC (ASIR, ASDR, and age-standardized DALYs) compared to females. However, noteworthy is the increasing trend in annual incidence rates for HNC in females (0.58; 95% CI: 0.54–0.62), surpassing males (0.2; 95% CI: 0.13–0.27). Simultaneously, females showed a smaller decrease in mortality rates (−0.5; 95% CI: [-0.56]-[-0.44]) compared to males (−0.64; 95% CI: [-0.69]-[-0.6]). Incorporating male to female ratios in our results visually illustrates yearly changes by gender. Previous research has also identified a faster increase in late-stage HNC incidence among females,.^22^ Although estrogen is beneficial for HNC,^23^ the mere level of estrogen alone cannot explain the higher increase in HNC incidence rates in females compared to males. We consider several factors contributing to the trends. First, studies have shown that the annual decline in alcohol consumption among females aged 15–39 is lower than that in males.^24^ Second, a lower prevalence of chewing tobacco use among females globally compared to males.^7^ This two factors indicating the need for attention to female-specific behavioral risk factors related to HNC. Third, the predominant representation of LC and LOC within HNC is also a contributing factor. we analyzed the EAPC of HNC burden across genders and revealed that the trend of LC and LOC corresponded to those of the overall HNC trends. Specifically, while the EAPC of ASIR in males with LOC has decreased, there has been an increase in females, a pattern consistent with existing research.^25^^,^^26^ Moreover, recurrence and second primary cancers (SPCs) continue to pose significant challenges to the long-term survival of HNC. Gender disparities in SPC may also be a contributing factor. Studies have revealed a higher risk of SPC occurrence among females in the LC group (HR = 1.74; 95% CI: 1.02–2.98).^27^ Based on our research findings, we need to pay special attention to the adverse trends in the disease burden of HNC among females, particularly in LC and LOC.

Based on global data, we found that in 2019, the cases of HNC in individuals under 20 showed a significant decrease in death and DALYs compared to 1990, despite an increase in incidence. Two aspects may explain this observation. First, elderly patients often have multiple underlying conditions, increasing the surgical risk and complications associated with comprehensive treatment for HNC. Second, aging itself may contribute to HNC-related mortality. Previous research has indicated that accelerated epigenetic aging is associated with more severe adverse events in HNC, including overall survival and progression-free survival.^28^ We also noticed that in low and middle-low SDI regions, the mortality and disability rates for HNC in individuals under 20 are still increasing. Therefore, special attention should be given to allocating medical resources for young HNC patients in these regions.

We observed significantly lower ASDR and age-standardized DALYs for TC compared to other subtypes. Although the ASDR for TC is almost negligible before age 50, it rises rapidly after 60, consistent with previous research findings.^29^ Current studies recommend reducing TC screening for adolescents,^30^ but we should pay attention to elderly TC patients, especially those with medullary and undifferentiated TC.^31^

### Conclusions

In conclusion, HNCs have shown an increasing ASIR globally over the past 30 years, while ASDR and age-standardized DALYs have decreased with improved prognosis. However, the decline trend has been slower in females than males, and young patients in underdeveloped regions have not experienced a decrease in death cases. Special attention is needed for elderly patients due to their mortality rates. We recommend implementing robust tobacco and alcohol control policies to reduce the incidence of HNC, and efficient allocation of healthcare resources to further reduce mortality and disability rates according to local needs.

### Limitations of the study

Our study presents a contribution as it represents the first investigation of the disease burden for all HNCs spanning the period from 1990 to 2019. Furthermore, unlike prior research focused on specific subtypes, our comprehensive approach aligns with the principles of otolaryngology and head and neck surgery. However, there are some limitations in our study that must be acknowledged. First, the GBD data source is constrained by the availability of primary data.^32^ While it relies on diverse data sources, including statistics agencies, health departments, and surveys, data quality issues such as inconsistent definitions, reporting errors, and methodological variations may impact research accuracy and comparability. Second, the accuracy of information in underdeveloped regions raises concerns, as the data may not fully reflect the true disease burden. Moreover, the use of EAPC to assess overall trends on a linear scale might potentially overlook detailed trends in age-standardized rate. Lastly, the findings of the GBD study may not adequately reflect significant health disparities among various regions and ethnicities worldwide, owing to limited data availability in certain regions or among specific ethnic groups.

## STAR★Methods

### Key resources table

**Table:** 

REAGENT or RESOURCE	SOURCE	IDENTIFIER
**Software and algorithms**		

R programming language 4.3.0	R Foundation, USA	https://www.r-project.org
R studio 2023.03.0	Posit, USA	https://posit.co/downloads/

### Resource availability

#### Lead contact

Further information and requests for resources should be directed to and will be fulfilled by the lead contact, Weijun Huang (hellohuangwj@126.com).

#### Materials availability

This study did not generate new unique reagents.

#### Data and code availability

• Data reported in this paper will be shared by the lead contact upon request.
• This paper does not report original code.
• Any additional information required to reanalyze the data reported in this paper is available from the lead contact upon request.

### Experimental model and study participant details

All data used in this study were obtained from the the Global Burden of Disease (GBD) database.

### Method details

#### Data source

Retrieve relevant keywords and download data from the GBD Results Tool on the GBD database website (http://ghdx.healthdata.org/gbd-2019), as detailed instructions on its specific usage can be found in other literature.^33^ We acquired specific data on the incidence, mortality, and DALYs for HNC from 1990 to 2019 globally, as well as in 204 countries or regions. This dataset encompasses various fields, including gender, age, and locations, which correspond to the evaluation indicators of disease burden.

#### Selection criteria

##### Age groups

Due to the absence of mortality or incidence rates for HNC in individuals aged 15 and below, we categorized age groups in five-year intervals starting from 20 years onwards. We divided the age groups into 16 categories, ranging from <20 years to >95years, to present disease burden indicators for each age group. Additionally, based on our preliminary data results and previous studies,^34^ we chose age cut-off points at 20, 50, and 70 years to subgroup the population into specific age groups for further analysis. This division allows for the presentation of variations in disease burden indicators among different age groups.

##### Location groups

SDI is a composite indicator of development status that is strongly correlated with health outcomes. It is used to assess the overall level of development in a particular region or country.^3^ In the GBD database, we obtained the original SDI data for 204 countries and grouped them into corresponding regions based on the five SDI levels (low, low-middle, middle, high-middle, and high). Additionally, considering socioeconomic similarities and geographical proximity, these countries and regions were further classified into 21 GBD regions.

### Quantification and statistical analysis

EAPC is utilized to quantify the trends in ASIR, ASDR, and age-standardized DALYs between 1990 and 2019. These trends are assessed using a linear regression equation, where the natural logarithm of age-standardized rate for each year represents the y-value, the year itself represents the x-value, and the annual percentage change is derived from the β-value using the equation: EAPC = 100 ∗ (exp (β) - 1). When the EAPC value has a 95% CI greater than 0, it signifies an increasing trend in the age-standardized indicators. Conversely, if the 95% CI is less than 0, it indicates a decreasing trend. ARCP can be calculated using the following formula: ARCP = ((P_2019 - P_1990) / P_1990∗(2019 - 1990). P_2019 represents the proportion of a specific subtype within all HNC subtypes in the year 2019. P_1990 represents the proportion of the same subtype within all HNC subtypes in the year 1990. By applying this formula, we can assess the annualized rate at which the composition of cancer subtypes changes over time within HNC. The data on incidence, death, DALYs, ASIR, ASDR, and age-standardized DALYs were combined with their corresponding 95% uncertainty interval (UI). Age-standardized rates are calculated per 10,000 populations. The numerical values of these disease burden indicators were presented in the form of bar charts, categorized by age location and SDI subgroups. The annual trends were visualized using line graphs. The regional differences were displayed using world heatmaps. To depict the relationship and annual trends between SDI and disease burden indicators, we employed fitted curves. To demonstrate the trends in subtypes of HNC across different disease burden evaluation indicators, the difference in the proportion between two specific years was divided by the starting year's proportion. The resulting ratio was presented to depict the trends in the subtypes. When describing trends, the terms "increasing" and "decreasing" were used if the slope of the trend had statistical significance. Otherwise, the term "stable" was used. All statistical analyses were performed using R project (version 4.3.0; R Foundation, Vienna, Austria). Two-sided p-value less than 0.05 was considered statistically significant.
