# Supplementary material for: Global burden of head and neck cancers from 1990 to 2019
Source: iScience. 2024 Feb 20;27(3):109282. doi: 10.1016/j.isci.2024.109282 (PMC10918270; doi:10.1016/j.isci.2024.109282)
Supplement: Document S1. Figures S1–S10 and Tables S1 and S2 [file mmc1.pdf]

iScience, Volume *27*

Supplemental information

Global burden of head

and neck cancers from 1990 to 2019

Tianjiao Zhou, Weijun Huang, Xiaoting Wang, Jingyu Zhang, Enhui Zhou, Yixing Tu, Jianyin Zou, Kaiming Su, Hongliang Yi, and Shankai Yin

Figure S1. Disease burden of head and neck cancer between 1990 and 2019 by different SDI level regions, Related to Figure 4

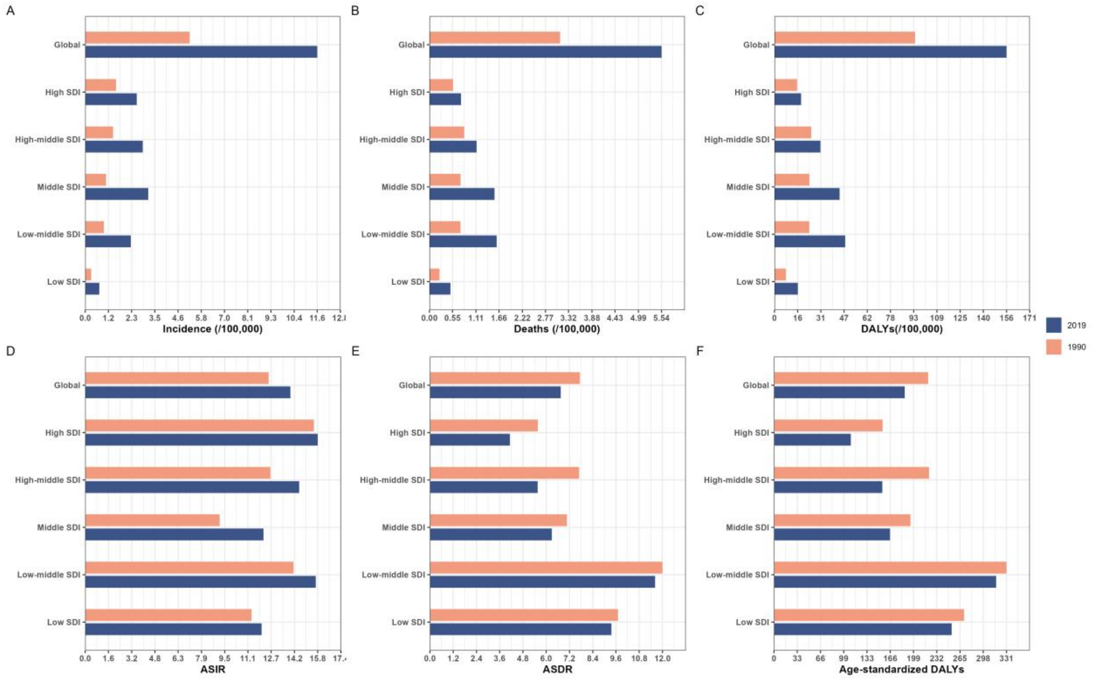

A. Trends in incidence of head and neck cancer between 1990 and 2019 by different SDI level regions.

B. Trends in deaths of head and neck cancer between 1990 and 2019 by different SDI level regions.

C. Trends in DALYs of head and neck cancer between 1990 and 2019 by different SDI level regions.

D. Trends in ASIR of head and neck cancer between 1990 and 2019 by different SDI level regions.

E. Trends in ASDR of head and neck cancer between 1990 and 2019 by different SDI level regions.

F. Trends in age-standardized DALYs of head and neck cancer between 1990 and 2019 by different SDI level regions.

ASIR, age-standardized incidence rate; ASDR, age-standardized death rate; DALYs, disability-adjusted life years; SDI, socio-demographic index.

Figure S2. Trends in disease burden of head and neck cancer from 1990 to 2019 by gender and male-to-female ratio, Related to Figure 4

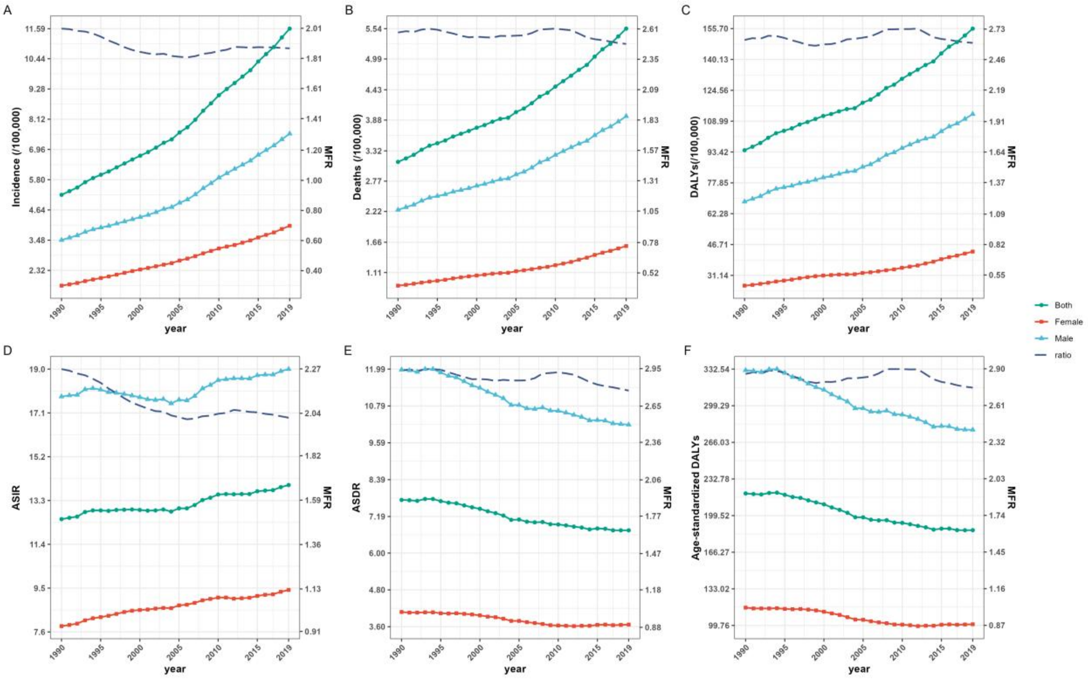

A. Trends in incidence of head and neck cancer from 1990 to 2019 by gender and MFR.

B. Trends in deaths of head and neck cancer from 1990 to 2019 by gender and MFR.

C. Trends in DALYs of head and neck cancer from 1990 to 2019 by gender and MFR.

D. Trends in ASIR of head and neck cancer from 1990 to 2019 by gender and MFR.

E. Trends in ASDR of head and neck cancer from 1990 to 2019 by gender and MFR.

F. Trends in age-standardized DALYs of head and neck cancer from 1990 to 2019 by gender and MFR.

ASIR, age-standardized incidence rate; ASDR, age-standardized death rate; DALYs, disability-adjusted life years; MFR mela-to-female ratio.

Figure S3. Trends in disease burden of head and neck cancer across different age groups (5 year intervals) in different SDI regions from 1990 to 2019, Related to Figure 5

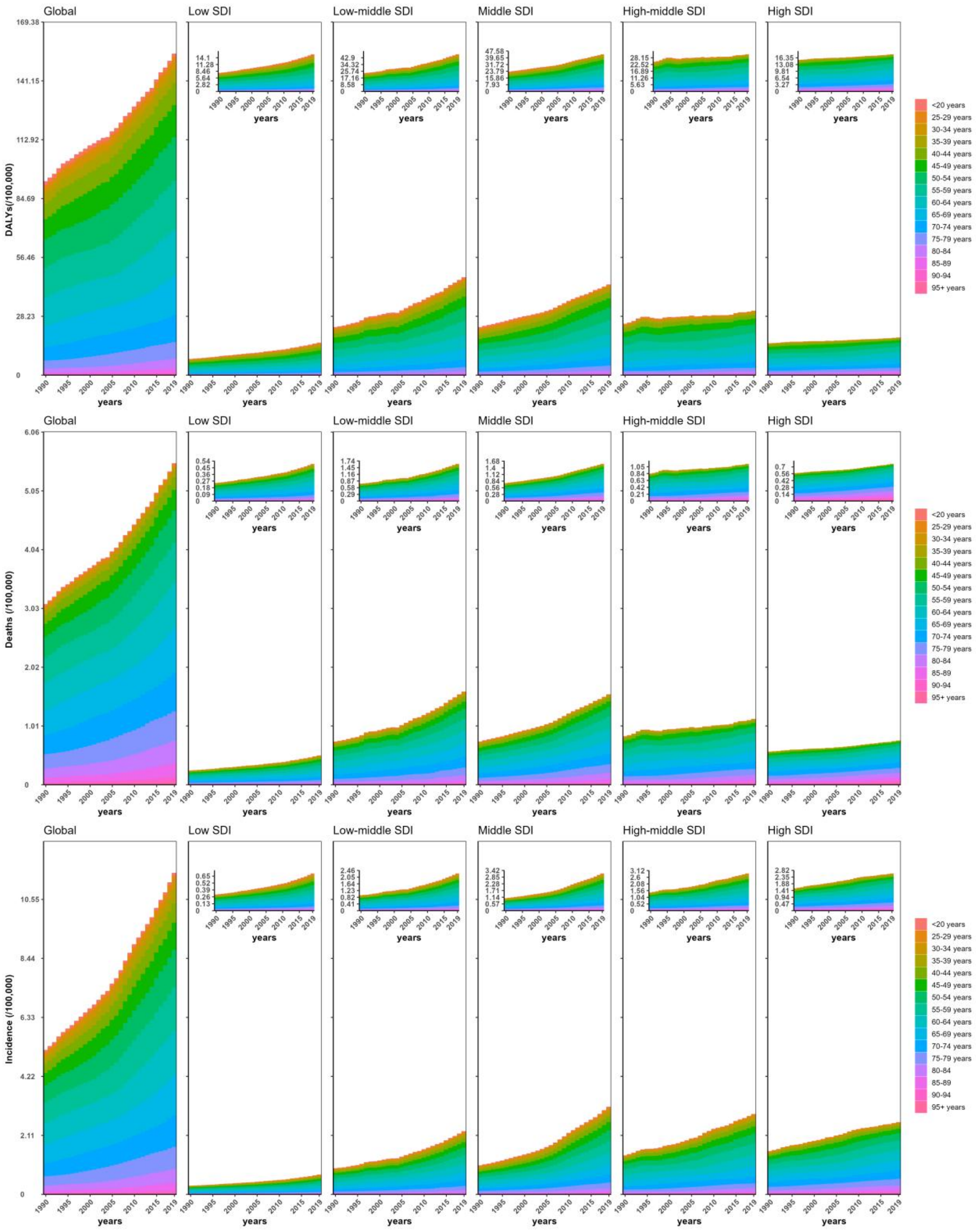

A. Trends in incidence of head and neck cancer across different age groups (5-year intervals) in different SDI regions from 1990 to 2019.

B. Trends in deaths of head and neck cancer across different age groups (5-year intervals) in different SDI regions from 1990 to 2019.

C. Trends in DALYs of head and neck cancer across different age groups (5-year intervals) in different SDI regions from 1990 to 2019.

DALYs, disability-adjusted life years; SDI, socio-demographic index.

Figure S4. Compare 1990 to 2019, the change precent of disease burden for head and neck cancer across different age groups (<20,20-49,50-69,>70 years) in different SDI regions, Related to Figure 5.

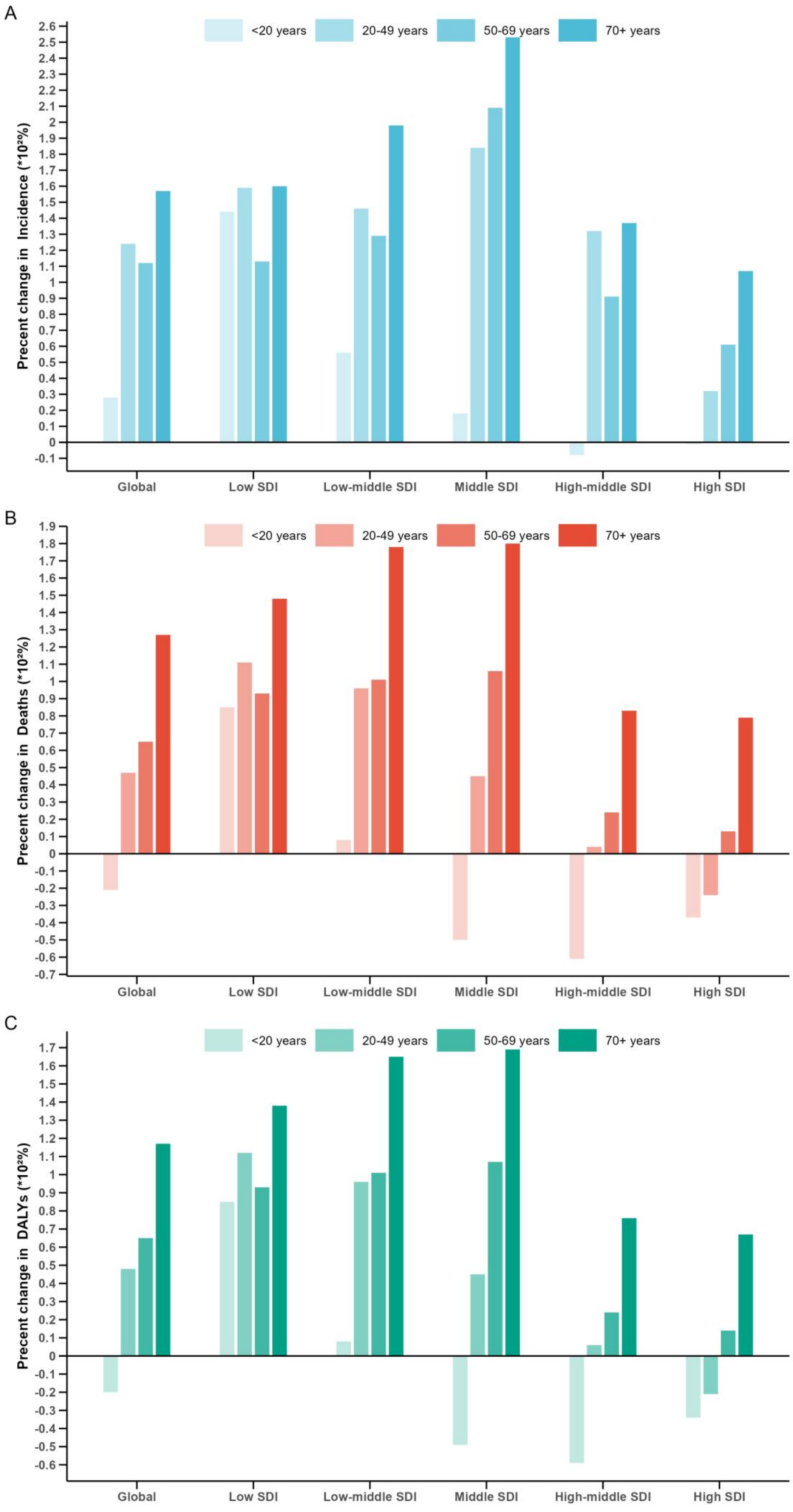

A
<20 years
20-49 years
50-69 years
70+ years
Precent change in Incidence (*10²%)
Global
Low SDI
Low-middle SDI
Middle SDI
High-middle SDI
High SDI
B
Precent change in Deaths (*10²%)
C
Precent change in DALYs (*10²%)

A. Compare 1990 to 2019, the change precent of incidence for head and neck cancer across different age groups (<20,20-49,50-69,>70 years) in different SDI regions.

B. Compare 1990 to 2019, the change precent of deaths for head and neck cancer across different age groups (<20,20-49,50-69,>70 years) in different SDI regions.

C. Compare 1990 to 2019, the change precent of DALYs for head and neck cancer across different age groups (<20,20-49,50-69,>70 years) in different SDI regions.

DALYs, disability-adjusted life years; SDI, socio-demographic index.

Figure S5. The proportion distribution of incidence, deaths, DALYs rates and the relative changes(ARCP)between 1990 and 2019 in the proportions of head and neck cancer subtypes, stratified by different SDI regions and genders, Related to Figure 6

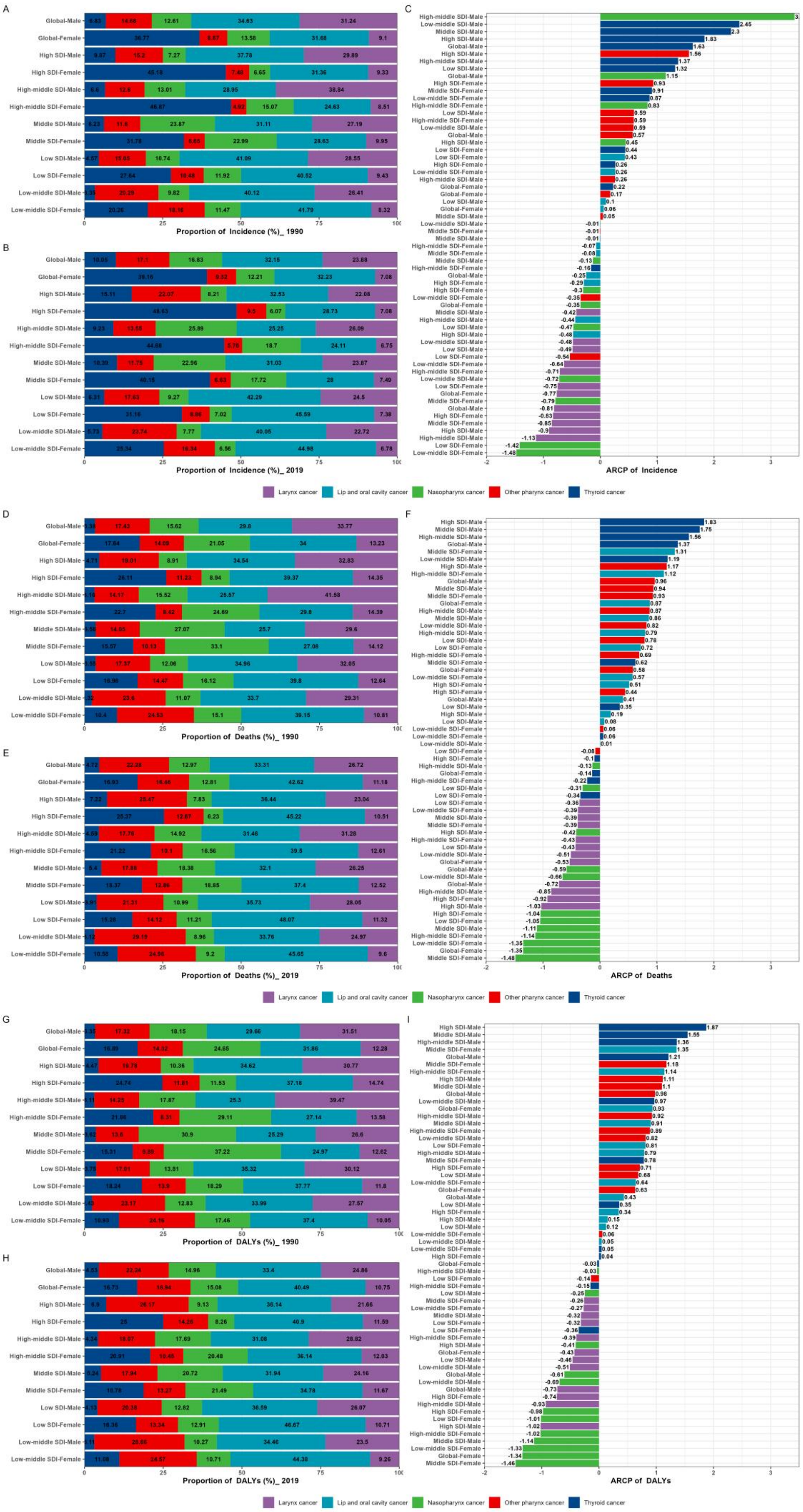
A
B
C
Proportion of Incidence (%)_ 1990
Proportion of Incidence (%)_ 2019
ARCP of Incidence
Larynx cancer
Lip and oral cavity cancer
Nasopharynx cancer
Other pharynx cancer
Thyroid cancer
D
E
F
Proportion of Deaths (%)_ 1990
Proportion of Deaths (%)_ 2019
ARCP of Deaths
Larynx cancer
Lip and oral cavity cancer
Nasopharynx cancer
Other pharynx cancer
Thyroid cancer
G
H
I
Proportion of DALYs (%)_ 1990
Proportion of DALYs (%)_ 2019
ARCP of DALYs
Larynx cancer
Lip and oral cavity cancer
Nasopharynx cancer
Other pharynx cancer
Thyroid cancer

A. The proportion distribution of incidences for different subtypes of head and neck cancer by SDI regions and genders in 1990.

B. The proportion distribution of incidences for different subtypes of head and neck cancer by SDI regions and genders in 2019.

C. The ARCP distribution of incidences for different subtypes of head and neck cancer by SDI regions and genders between 1990 and 2019.

D. The proportion distribution of deaths for different subtypes of head and neck cancer by SDI regions and genders in 1990.

E. The proportion distribution of deaths for different subtypes of head and neck cancer by SDI regions and genders in 2019.

F. The ARCP distribution of deaths for different subtypes of head and neck cancer by SDI regions and genders between 1990 and 2019.

G. The proportion distribution of DALYs for different subtypes of head and neck cancer by SDI regions and genders in 1990.

H. The proportion distribution of DALYs for different subtypes of head and neck cancer by SDI regions and genders in 2019.

I. The ARCP distribution of DALYs for different subtypes of head and neck cancer by SDI regions and genders between 1990 and 2019.

ARCP, annual rate of change in the proportion; DALYs, disability-adjusted life years; SDI, socio-demographic index.

Figure S6. Disease burden of larynx cancer across different age groups (5-year intervals) by gender in 2019, Related to Figure 5

Larynx cancer

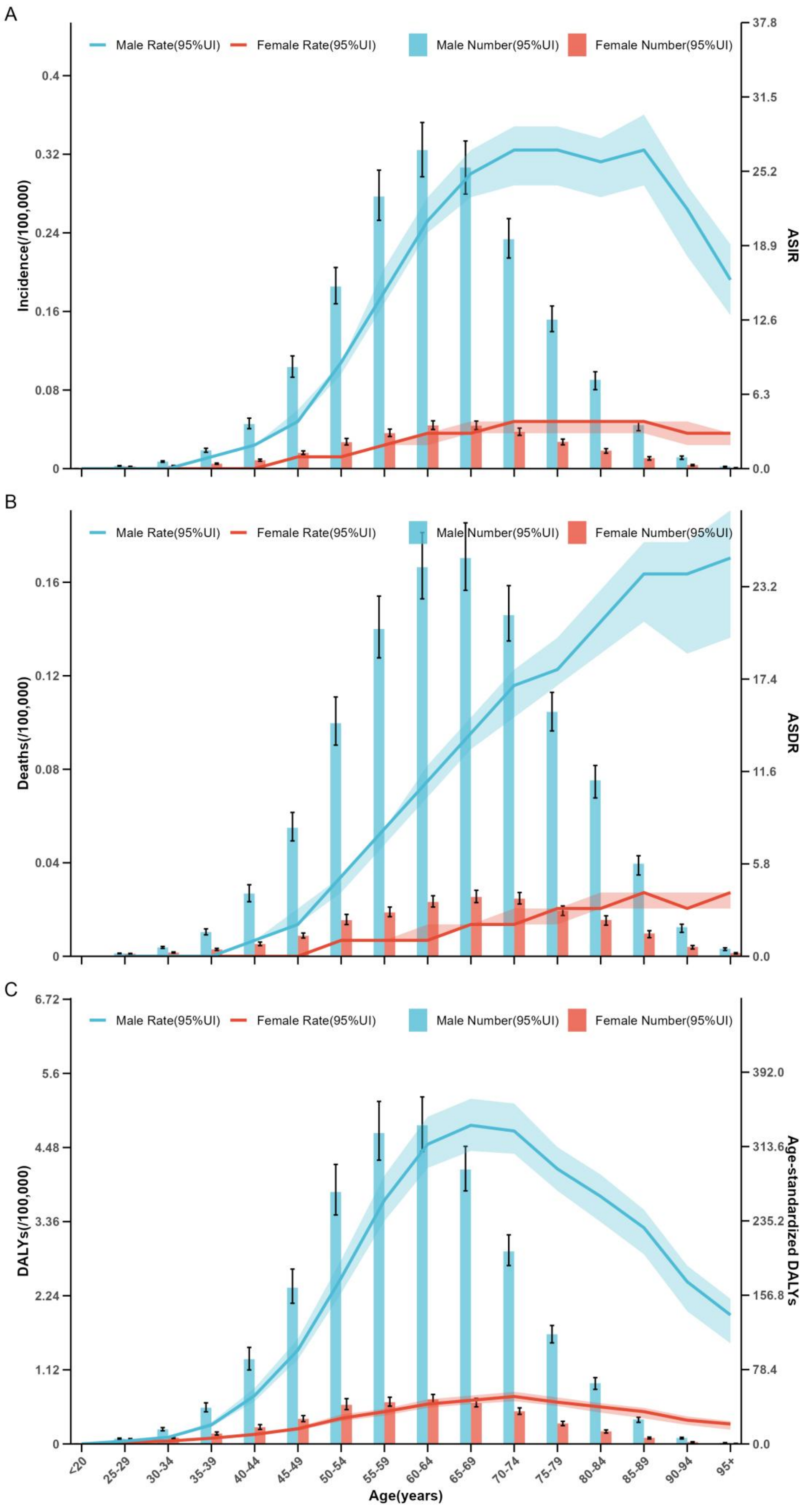

A. Incidence and ASIR of larynx cancer across different age groups (5-year intervals) by gender in 2019.

B. Deaths and ASDR of larynx cancer across different age groups (5-year intervals) by gender in 2019.

C. DALYs and age-standardized DALYs of larynx cancer across different age groups (5-year intervals) by gender in 2019.

ASIR, age-standardized incidence rate; ASDR, age-standardized death rate; DALYs, disability-adjusted life years.

Figure S7. Disease burden of lip and oral cavity cancer across different age groups (5-year intervals) by gender in 2019, Related to Figure 5

Lip and oral cavity cancer

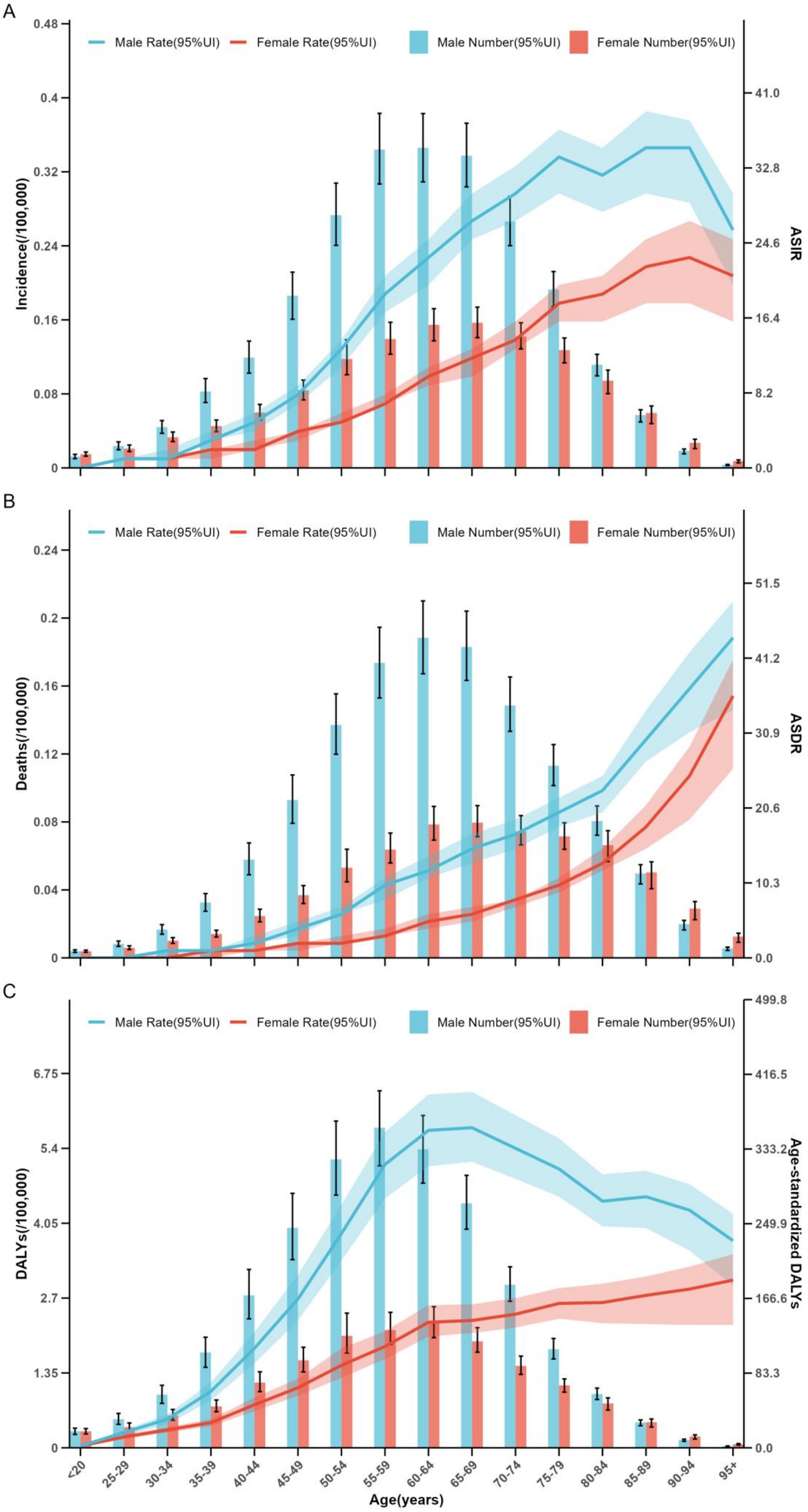

A. Incidence and ASIR of lip and oral cavity cancer across different age groups (5-year intervals) by gender in 2019.

B. Deaths and ASDR of lip and oral cavity cancer across different age groups (5-year intervals) by gender in 2019.

C. DALYs and age-standardized DALYs of lip and oral cavity cancer across different age groups (5-year intervals) by gender in 2019.

ASIR, age-standardized incidence rate; ASDR, age-standardized death rate; DALYs, disability-adjusted life years.

Figure S8. Disease burden of nasopharynx cancer across different age groups (5-year intervals) by gender in 2019, Related to Figure 5

Nasopharynx cancer

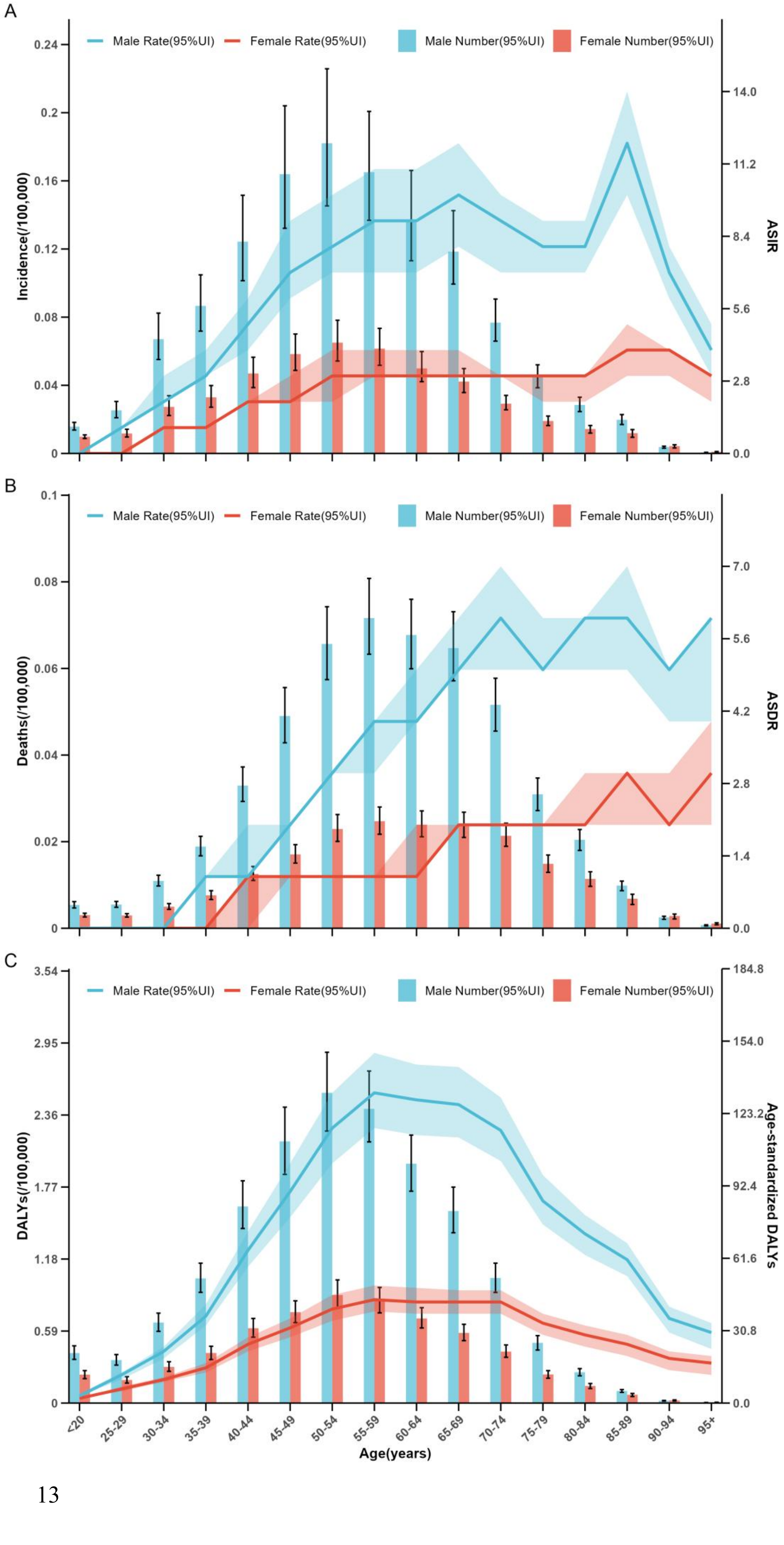

A. Incidence and ASIR of nasopharynx cancer across different age groups (5-year intervals) by gender in 2019.

B. Deaths and ASDR of nasopharynx cancer across different age groups (5-year intervals) by gender in 2019.

C. DALYs and age-standardized DALYs of nasopharynx cancer across different age groups (5-year intervals) by gender in 2019.

ASIR, age-standardized incidence rate; ASDR, age-standardized death rate; DALYs, disability-adjusted life years.

Figure S9. Disease burden of other pharynx cancer across different age groups (5-year intervals) by gender in 2019, Related to Figure 5

Other pharynx cancer

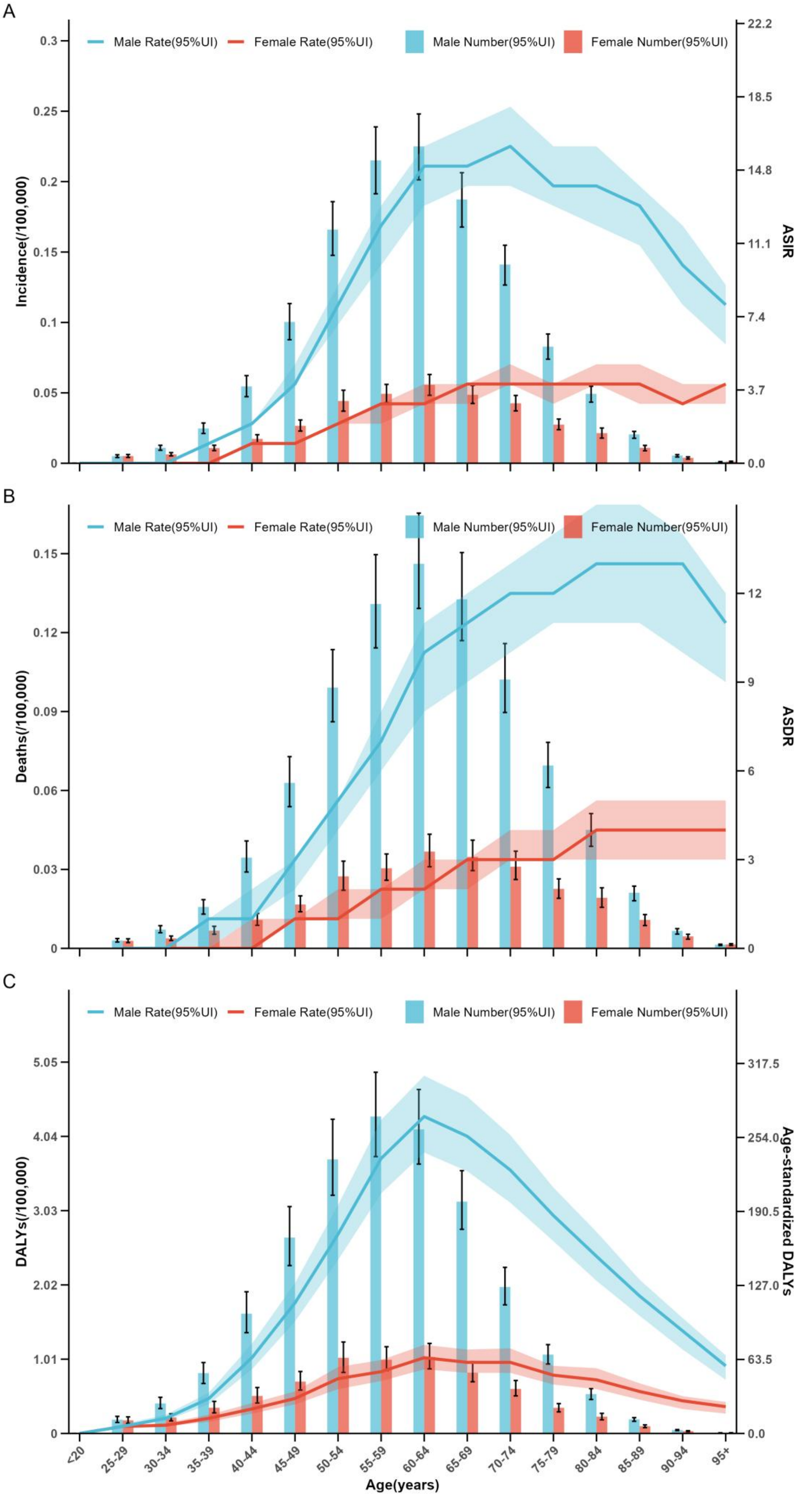

A. Incidence and ASIR of other pharynx cancer across different age groups (5-year intervals) by gender in 2019.

B. Deaths and ASDR of other pharynx cancer across different age groups (5-year intervals) by gender in 2019.

C. DALYs and age-standardized DALYs of other pharynx cancer across different age groups (5-year intervals) by gender in 2019.

ASIR, age-standardized incidence rate; ASDR, age-standardized death rate; DALYs, disability-adjusted life years.

Figure S10. Disease burden of thyroid cancer across different age groups (5-year intervals) by gender in 2019, Related to Figure 5

Thyroid cancer

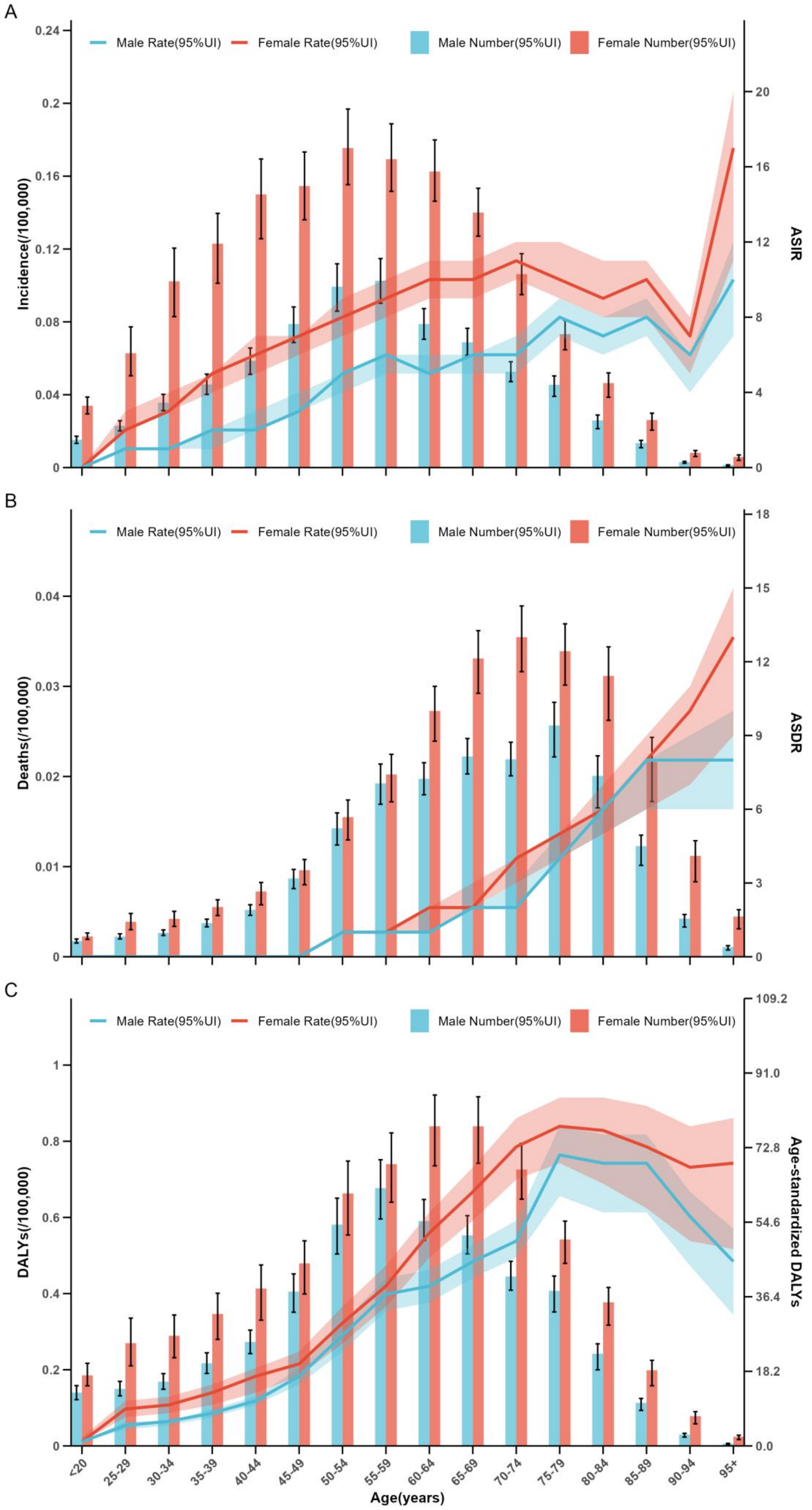

A. Incidence and ASIR of thyroid cancer across different age groups (5-year intervals) by gender in 2019.

B. Deaths and ASDR of thyroid cancer across different age groups (5-year intervals) by gender in 2019.

C. DALYs and age-standardized DALYs of thyroid cancer across different age groups (5-year intervals) by gender in 2019.

ASIR, age-standardized incidence rate; ASDR, age-standardized death rate; DALYs, disability-adjusted life years.

Table S1 Global Burden of Head and Neck Cancer and Trends from 1990 to 2019 by 204 countries, Related to Figure 1

Characteristics	1990		2019			1990		2019			1990		2019		
	Incidence cases (95%UI)	ASIR pre 100,000(95%UI)	Incidence cases (95%UI)	ASIR pre 100,000(95%UI)	EAPC (95%CI)	Death cases (95%UI)	ASDR pre 100,000(95%UI)	Death cases (95%UI)	ASDR pre 100,000(95%UI)	EAPC (95%CI)	DALYs cases (95%UI)	Age_standardised DALYs pre 100,000(95%UI)	DALYs cases (95%UI)	Age_standardised DALYs pre 100,000(95%UI)	EAPC (95%CI)
Afghanistan	614(368-913)	8.21(5.04-11.99)	1279(765-1894)	8.02(5.17-11.22)	-0.1(-0.14 to -0.05)	508(320-734)	7.05(4.59-10.01)	834(546-1160)	6.24(4.31-8.41)	-0.46(-0.55 to -0.37)	15163(9066-22393)	194.85(117.95-285.57)	28254(17502-40859)	167.23(110-232.22)	-0.59(-0.7 to -0.48)
Albania	224(200-244)	10.21(9.15-11.13)	375(274-502)	9.49(6.95-12.7)	-0.03(-0.31-0.24)	154(140-167)	7.57(6.89-8.24)	199(146-265)	4.74(3.5-6.29)	-1.39(-1.69 to -1.09)	4243(3865-4628)	186.02(169.4-202.68)	4680(3398-6337)	117.24(85.35-158.58)	-1.38(-1.67 to -1.08)
Algeria	1612(1249-2041)	11.22(8.84-14.1)	4926(3580-6513)	12.56(9.34-16.34)	0.39(0.24-0.55)	998(791-1258)	8.04(6.43-10.08)	1739(1354-2200)	5.15(4.04-6.49)	-1.49(-1.62 to -1.37)	33608(26657-41987)	225.9(178.88-283.86)	54268(41544-69066)	141.93(109.68-179.75)	-1.59(-1.7 to -1.47)
American Samoa	2(1-2)	6.97(5.81-8.28)	4(3-5)	7.99(6.26-9.95)	0.72(0.57-0.86)	1(1-1)	4.88(4.1-5.75)	2(2-3)	4.7(3.71-5.72)	0.07(-0.06-0.2)	31(26-37)	118.98(100.03-139.98)	60(47-74)	117.91(93.25-145.19)	0.17(0.05-0.28)
Andorra	10(7-14)	17.26(12.52-23.67)	22(16-30)	16.43(12.05-21.84)	-0.33(-0.46 to -0.21)	3(2-5)	6.1(4.5-8.28)	6(4-8)	4.23(3.17-5.48)	-1.42(-1.47 to -1.37)	98(71-135)	168.47(121.64-230.45)	163(120-216)	118.04(86.58-156.74)	-1.4(-1.46 to -1.35)
Angola	282(198-380)	6.41(4.5-8.55)	843(624-1122)	6.71(5.06-8.79)	0.12(0.04-0.21)	235(165-316)	5.77(4.02-7.62)	628(472-824)	5.56(4.23-7.21)	-0.17(-0.22 to -0.11)	7604(5303-10274)	156.84(109.56-210.44)	19776(14581-26092)	143.35(107.73-187.79)	-0.36(-0.43 to -0.29)
Antigua and Barbuda	5(4-5)	9.38(8.31-10.58)	10(8-12)	9.94(8.19-11.93)	0.27(-0.02-0.57)	3(3-4)	6.25(5.58-7)	6(5-7)	5.59(4.68-6.61)	-0.31(-0.61 to -0.01)	79(70-88)	157.09(139.31-177.19)	139(114-167)	131.48(108.2-157.29)	-0.56(-0.84 to -0.28)
Argentina	3487(3238-3719)	10.75(9.98-11.46)	4746(3681-6057)	9.13(7.07-11.66)	-0.92(-1.05 to -0.78)	2270(2137-2397)	7.05(6.63-7.44)	2529(2316-2760)	4.74(4.35-5.18)	-1.61(-1.72 to -1.5)	63027(59060-66795)	193.72(181.44-205.36)	62776(57391-68995)	121.39(110.86-133.48)	-1.92(-2.04 to -1.8)
Armenia	254(232-281)	8.56(7.88-9.43)	352(287-424)	8.54(7-10.26)	-0.16(-0.38-0.06)	185(170-202)	6.46(5.95-7.05)	204(168-244)	4.88(4.03-5.82)	-1.26(-1.43 to -1.09)	5525(5075-6097)	180.19(166.14-197.62)	5251(4299-6340)	126.89(104.49-152.35)	-1.53(-1.72 to -1.33)
Australia	2902(2723-3099)	15.24(14.28-16.26)	4719(3602-6112)	13.01(9.88-16.88)	-0.45(-0.58 to -0.33)	1448(1377-1516)	7.54(7.16-7.89)	1805(1605-1987)	4.43(3.96-4.85)	-1.89(-1.98 to -1.79)	37465(35710-39321)	197.84(188.43-207.86)	41440(37252-45703)	112.44(101.51-123.88)	-1.95(-2.05 to -1.86)
Austria	1551(1420-1676)	15(13.69-16.26)	2208(1738-2777)	14.73(11.54-18.6)	-0.26(-0.41 to -0.1)	664(621-703)	6.05(5.66-6.41)	782(706-862)	4.71(4.26-5.19)	-0.9(-0.97 to -0.83)	17816(16634-18986)	173.89(162.39-185.58)	19212(17353-21288)	127.53(115.01-141.46)	-1.17(-1.27 to -1.07)
Azerbaijan	366(320-420)	6.6(5.78-7.57)	733(570-952)	7.04(5.55-9.09)	0.15(-0.03-0.33)	263(231-301)	4.95(4.35-5.68)	429(334-564)	4.51(3.57-5.92)	-0.4(-0.54 to -0.27)	8384(7362-9543)	147.25(129.69-167.41)	13071(10136-16970)	120.26(94.39-156.32)	-0.92(-1.07 to -0.77)
Bahamas	24(22-27)	14.6(13.09-16.28)	54(43-67)	13.08(10.46-16.29)	-0.2(-0.32 to -0.08)	16(14-17)	10.09(9.09-11.18)	32(26-40)	8.12(6.57-10.01)	-0.6(-0.76 to -0.43)	455(407-509)	269.18(240.79-300.96)	903(715-1135)	212.71(169.46-266.2)	-0.66(-0.85 to -0.48)
Bahrain	18(15-22)	9.48(7.63-11.48)	92(69-120)	8.13(6.12-10.34)	-0.81(-1.09 to -0.54)	11(9-13)	7(5.57-8.44)	29(21-37)	3.68(2.69-4.68)	-2.73(-3.04 to -2.41)	311(253-376)	153.01(122.89-184.82)	864(643-1132)	77.04(57.36-98.97)	-2.95(-3.24 to -2.66)
Bangladesh	9610(7252-12328)	18.69(14.21-23.95)	20024(13872-28746)	14.68(10.22-20.86)	-0.87(-0.99 to -0.76)	7821(5962-9997)	16.1(12.35-20.48)	14395(10108-20431)	11.02(7.8-15.55)	-1.33(-1.46 to -1.2)	244809(183845-315227)	447.37(338.55-576.37)	409946(284008-591803)	293.04(203.95-420.41)	-1.44(-1.54 to -1.35)
Barbados	27(24-29)	9.8(8.94-10.71)	50(41-61)	10.81(8.77-13.16)	0.17(0.08-0.27)	19(17-20)	6.53(5.98-7.1)	30(25-36)	6.13(5.02-7.36)	-0.45(-0.58 to -0.32)	428(392-466)	163.2(149.33-178.1)	697(563-848)	148.18(119.8-180.54)	-0.52(-0.64 to -0.41)
Belarus	1909(1755-2080)	14.86(13.68-16.2)	2367(1774-3161)	15.86(11.86-21.13)	-0.31(-0.53 to -0.09)	1296(1201-1402)	9.97(9.25-10.75)	1245(943-1645)	7.99(6.06-10.52)	-1.38(-1.65 to -1.1)	38873(35830-42260)	303.34(280.05-329.72)	35691(26729-47420)	237.55(177.72-315.43)	-1.51(-1.8 to -1.21)
Belgium	2471(2265-2679)	17.83(16.35-19.4)	3152(2389-4046)	16.73(12.6-21.54)	-0.39(-0.49 to -0.28)	1038(971-1104)	7.15(6.69-7.6)	1033(933-1137)	4.89(4.43-5.38)	-1.46(-1.55 to -1.37)	26949(25215-28720)	198.03(185.05-211.16)	25235(22738-28009)	134.22(120.85-149.11)	-1.59(-1.69 to -1.49)
Belize	5(4-5)	4.74(4.24-5.24)	20(17-23)	6.62(5.62-7.7)	1(0.64-1.35)	3(3-4)	3.63(3.28-4.01)	12(11-14)	4.44(3.79-5.14)	0.52(0.13-0.91)	86(78-95)	88.56(79.91-98.15)	355(301-414)	114.72(97.4-133.61)	0.7(0.32-1.08)
Benin	83(67-101)	3.95(3.19-4.76)	230(169-307)	4.34(3.28-5.69)	0.44(0.38-0.49)	68(55-82)	3.42(2.8-4.08)	174(130-228)	3.61(2.77-4.63)	0.34(0.28-0.4)	1931(1550-2356)	88.04(71.08-107.18)	5137(3712-6956)	90.28(66.7-120.02)	0.19(0.14-0.25)
Bermuda	10(9-12)	16.57(14.8-18.56)	17(14-20)	14.11(11.46-17.33)	-0.37(-0.57 to -0.16)	6(6-7)	10.06(9.03-11.19)	8(6-9)	5.85(4.85-7.1)	-1.69(-1.93 to -1.44)	157(140-176)	246.36(219.7-275.44)	168(137-207)	140.17(113.87-173.3)	-1.76(-2.03 to -1.48)
Bhutan	43(28-60)	15.32(10.23-20.99)	102(68-142)	17.05(11.62-23.34)	0.31(0.23-0.4)	34(23-48)	13.13(8.77-17.92)	70(48-96)	12.57(8.66-16.95)	-0.22(-0.27 to -0.17)	1124(724-1561)	366.35(241.2-507.25)	1961(1282-2805)	320.34(212.24-451.15)	-0.56(-0.63 to -0.48)
olivia (Plurinational State o	218(163-277)	6.35(4.77-8.07)	717(520-951)	7.81(5.71-10.29)	0.62(0.53-0.71)	166(126-209)	5.29(4.06-6.64)	426(315-550)	5.06(3.77-6.49)	-0.2(-0.28 to -0.12)	4679(3535-5909)	130.79(98.96-165.15)	10614(7850-13970)	114.94(85.14-150.24)	-0.53(-0.61 to -0.45)
Bosnia and Herzegovina	450(398-491)	10.32(9.18-11.21)	599(454-772)	10.53(7.98-13.62)	-0.1(-0.19 to -0.01)	313(285-338)	7.55(6.87-8.11)	352(272-445)	5.98(4.63-7.58)	-1.17(-1.33 to -1.02)	8974(8158-9753)	197.18(179.66-212.96)	8611(6585-11100)	150.7(115.11-193.87)	-1.35(-1.52 to -1.18)
Botswana	52(39-69)	8.41(6.33-10.91)	157(107-219)	10.18(7.16-13.83)	0.07(-0.39-0.53)	40(30-53)	6.82(5.14-8.78)	105(72-144)	7.29(5.23-9.81)	-0.33(-0.81-0.16)	1231(895-1638)	187.61(137.82-247.14)	3293(2197-4640)	199.15(136.28-275.31)	-0.44(-0.99-0.12)
Brazil	10899(10501-11332)	11.4(10.97-11.85)	26997(25363-28748)	11.14(10.45-11.85)	-0.09(-0.25-0.06)	7667(7374-7968)	8.52(8.14-8.87)	16601(15521-17627)	6.94(6.48-7.37)	-0.7(-0.82 to -0.59)	226834(218674-235624)	228.19(219.69-236.93)	453525(427673-482369)	184.9(174.3-196.56)	-0.76(-0.91 to -0.61)
Brunei Darussalam	26(21-31)	21.96(17.9-26.35)	72(59-88)	20.53(17.05-24.45)	0(-0.15-0.16)	14(12-17)	14.94(12.31-17.65)	32(27-37)	11.1(9.46-12.95)	-0.73(-0.89 to -0.56)	464(385-554)	365.87(301.85-433.25)	982(826-1165)	267.93(227.59-314.59)	-0.8(-1 to -0.6)
Bulgaria	1298(1182-1415)	10.8(9.84-11.78)	1883(1444-2418)	15.7(11.98-20.27)	1.76(1.53-1.98)	722(666-780)	5.94(5.49-6.41)	953(742-1207)	7.45(5.77-9.47)	1.37(1.1-1.64)	21488(19727-23364)	179.67(165.02-195.22)	26148(20044-33428)	223.71(171.22-287.56)	1.29(1.03-1.55)
Burkina Faso	187(139-242)	4.08(3.07-5.2)	480(359-622)	4.84(3.67-6.2)	0.63(0.51-0.75)	152(115-194)	3.53(2.7-4.46)	364(276-469)	4.03(3.09-5.12)	0.53(0.41-0.65)	4405(3267-5686)	90.51(67.86-116.1)	10864(8111-14273)	102.01(76.82-131.56)	0.42(0.28-0.56)
Burundi	283(198-393)	10.6(7.56-14.6)	491(337-691)	8.64(6.03-12.09)	-0.96(-1.06 to -0.86)	230(165-313)	9.16(6.67-12.41)	357(246-503)	7(4.89-9.74)	-1.23(-1.34 to -1.11)	7585(5329-10531)	272.85(193.45-377.5)	12139(8251-17284)	201.1(138.45-284.24)	-1.39(-1.52 to -1.26)
Cabo Verde	8(6-9)	3.33(2.77-3.85)	40(32-50)	8.94(7.23-10.97)	2.9(2.33-3.48)	6(5-7)	2.75(2.26-3.19)	26(22-32)	6.03(4.93-7.36)	2.04(1.47-2.61)	153(127-178)	67.37(55.69-78.53)	689(552-860)	149.19(120.51-185.06)	2.23(1.65-2.82)
Cambodia	533(382-689)	10.59(7.79-13.51)	1608(1128-2122)	12.63(8.98-16.45)	0.54(0.42-0.66)	397(296-506)	8.59(6.49-10.91)	980(720-1256)	8.41(6.23-10.67)	-0.14(-0.24 to -0.04)	12552(9069-16217)	234.24(173-300.18)	27779(20194-36264)	212.12(154.8-275.18)	-0.43(-0.54 to -0.32)
Cameroon	253(196-321)	5.13(4.01-6.5)	810(570-1128)	6.04(4.4-8.22)	0.65(0.55-0.75)	208(163-264)	4.52(3.56-5.69)	618(445-851)	5.03(3.74-6.72)	0.49(0.36-0.61)	6544(5095-8316)	122.27(95.47-155.45)	19624(13549-27558)	132.68(94.8-184.57)	0.36(0.22-0.49)
Canada	4835(4495-5192)	15.33(14.23-16.48)	7636(5816-9861)	12.64(9.61-16.36)	-0.83(-0.92 to -0.74)	1809(1716-1890)	5.67(5.38-5.93)	2402(2152-2640)	3.52(3.17-3.85)	-1.88(-1.98 to -1.79)	46539(44340-48717)	148.54(141.49-155.67)	54473(48744-60239)	87.72(78.75-96.87)	-2.06(-2.16 to -1.96)
Central African Republic	104(74-142)	7.84(5.73-10.56)	163(109-235)	6.5(4.51-9.2)	-0.64(-0.68 to -0.61)	88(63-120)	7.08(5.25-9.51)	136(92-195)	5.83(4.08-8.24)	-0.69(-0.72 to -0.65)	2833(1993-3924)	198.54(142.29-272.27)	4481(2958-6577)	161.7(109.89-232.24)	-0.75(-0.79 to -0.7)
Chad	97(74-121)	3.31(2.54-4.1)	264(196-341)	4.36(3.28-5.58)	1.1(0.99-1.22)	81(63-100)	2.9(2.25-3.57)	213(160-273)	3.82(2.91-4.84)	1.14(0.99-1.29)	2292(1760-2847)	75.27(57.79-93.35)	6319(4660-8244)	96.52(71.93-124.92)	1.01(0.85-1.18)
Chile	679(624-739)	6.57(6.05-7.12)	1643(1257-2111)	7.05(5.39-9.07)	0.46(0.35-0.56)	395(366-425)	4.07(3.77-4.37)	667(597-736)	2.78(2.49-3.07)	-1.23(-1.33 to -1.12)	10163(9424-10969)	97.7(90.65-105.43)	15076(13584-16699)	63.62(57.39-70.4)	-1.38(-1.5 to -1.26)
China	71875(61428-82494)	7.73(6.66-8.85)	250176(205353-301716)	12.63(10.45-15.17)	2.02(1.79-2.26)	51778(44485-59443)	6.01(5.2-6.87)	84385(69996-100582)	4.27(3.56-5.06)	-1.09(-1.18 to -0.99)	667999(1423021-1919717	170.38(145.89-195.44)	318632(1925545-2768286	114.37(95.42-135.81)	-1.37(-1.46 to -1.28)
Colombia	1614(1503-1732)	8.59(8-9.17)	3624(2730-4730)	6.9(5.2-9.02)	-1.04(-1.23 to -0.85)	1018(954-1079)	6.03(5.6-6.41)	1725(1328-2196)	3.24(2.48-4.13)	-2.49(-2.65 to -2.32)	27483(25847-29134)	143.01(134.38-151.33)	38807(29433-50251)	73.78(56.03-95.55)	-2.64(-2.82 to -2.45)
Comoros	17(8-24)	6.87(3.45-9.68)	40(28-57)	7.52(5.37-10.36)	0.16(-0.02-0.33)	14(7-19)	5.91(3.2-8.17)	29(21-39)	5.71(4.22-7.71)	-0.29(-0.45 to -0.14)	425(179-620)	168.15(76.46-242.03)	883(597-1251)	160.11(111.14-224.17)	-0.4(-0.62 to -0.19)
Congo	100(75-127)	8.6(6.61-10.73)	204(147-276)	7.03(5.28-9.31)	-0.81(-1 to -0.62)	82(62-103)	7.49(5.84-9.22)	150(111-202)	5.72(4.38-7.52)	-1.08(-1.26 to -0.9)	2539(1882-3258)	204.97(154.13-259.64)	4618(3297-6333)	146.56(108.09-197.16)	-1.34(-1.53 to -1.14)
Cook Islands	1(1-1)	7.48(6.12-9.1)	2(1-2)	7.01(5.42-8.86)	-0.46(-0.63 to -0.29)	0(0-1)	3.86(3.2-4.57)	1(1-1)	2.9(2.38-3.52)	-1.08(-1.19 to -0.97)	13(11-16)	96.61(79.28-116.86)	17(14-21)	73.11(57.48-91.01)	-1.02(-1.17 to -0.87)
Costa Rica	175(158-192)	9.06(8.21-9.93)	456(343-594)	8.84(6.65-11.51)	-0.32(-0.54 to -0.09)	89(82-97)	5.12(4.67-5.58)	198(151-254)	3.87(2.95-4.96)	-1.21(-1.4 to -1.03)	2446(2244-2653)	128.81(117.88-139.91)	4921(3709-6386)	95.22(71.95-123.37)	-1.31(-1.52 to -1.11)
C⁄₄te d'Ivoire	220(169-277)	4.78(3.72-5.91)	589(424-775)	5(3.74-6.38)	0.04(-0.08-0.16)	169(131-213)	4.1(3.24-5.05)	447(328-579)	4.22(3.22-5.32)	0.01(-0.12-0.13)	5463(4167-6923)	107.37(83.07-134.6)	13814(9817-18379)	106.52(78.08-138.37)	-0.18(-0.34 to -0.03)
Croatia	1513(1356-1676)	23.38(21-25.87)	1311(997-1694)	17.36(13.14-22.63)	-0.95(-1.05 to -0.84)	789(717-868)	12.18(11.1-13.36)	581(445-744)	7.14(5.45-9.22)	-1.88(-1.97 to -1.78)	23052(20864-25425)	353.76(320.54-389.32)	14637(11109-19053)	196.38(148.55-256.05)	-2.14(-2.24 to -2.03)
Cuba	1509(1408-1611)	14.63(13.66-15.63)	3584(2861-4465)	19.52(15.54-24.33)	1.19(1.06-1.32)	961(904-1018)	9.42(8.85-9.98)	1959(1584-2404)	10.27(8.29-12.6)	0.46(0.34-0.58)	23521(22159-24935)	228.77(215.44-242.54)	47095(37421-58751)	255.06(202.59-317.69)	0.57(0.46-0.69)
Cyprus	65(52-76)	8.11(6.49-9.41)	176(145-212)	9.56(7.9-11.53)	0.56(0.37-0.75)	29(24-34)	3.84(3.14-4.39)	53(44-61)	2.81(2.39-3.26)	-1.23(-1.32 to -1.13)	738(604-849)	91.55(75.08-105.49)	1249(1057-1461)	67.44(57.23-78.87)	-1.19(-1.27 to -1.1)
Czechia	1991(1860-2129)	15.33(14.31-16.4)	2704(2140-3377)	15.11(11.85-18.98)	0.05(-0.11-0.2)	1043(983-1097)	7.87(7.44-8.29)	1103(884-1366)	5.72(4.57-7.12)	-0.88(-0.96 to -0.79)	29719(27975-31408)	230.42(217.09-243.71)	28311(22450-35410)	159.67(126.08-200.41)	-1.1(-1.19 to -1)
cratic People's Republic of	1675(1156-2321)	9.09(6.45-12.35)	2662(1981-3565)	8.18(6.11-10.92)	-0.34(-0.48 to -0.21)	906(648-1226)	5.3(3.89-7)	1491(1153-1909)	4.6(3.59-5.87)	-0.47(-0.51 to -0.43)	30263(20827-42407)	159.16(112.69-218.76)	44088(32491-58570)	133.26(98.94-176.76)	-0.62(-0.65 to -0.6)
nocratic Republic of the Co	975(727-1270)	5.71(4.26-7.41)	2066(1454-2821)	5.25(3.68-7.15)	-0.36(-0.48 to -0.25)	794(593-1033)	5.05(3.79-6.54)	1586(1115-2160)	4.41(3.08-6.01)	-0.52(-0.62 to -0.42)	24383(18200-31760)	130.74(97.91-169.52)	48903(34641-66683)	114.05(80.11-155.23)	-0.53(-0.63 to -0.44)
Denmark	750(693-816)	10.41(9.58-11.35)	1554(1186-2027)	15.8(11.98-20.71)	1.16(0.69-1.63)	326(305-350)	4.28(4-4.59)	512(452-574)	4.71(4.17-5.26)	-0.05(-0.49-0.4)	8164(7617-8788)	115.65(107.67-124.78)	12061(10670-13664)	122.85(108.38-139.16)	-0.19(-0.65-0.27)
Djibouti	14(10-21)	7.83(5.51-11.19)	67(43-103)	8.94(6.14-13.14)	0.42(0.37-0.47)	11(8-16)	6.69(4.77-9.46)	45(30-68)	6.87(4.88-9.79)	0.02(-0.07-0.1)	391(262-576)	191.15(131.48-277.44)	1527(965-2336)	191.08(128.33-284.29)	-0.09(-0.19-0.01)
Dominica	8(7-10)	12.28(10.58-14.19)	11(9-14)	12.39(9.72-15.52)	0.16(0.07-0.26)	6(6-7)	8.97(7.79-10.26)	8(6-10)	8.86(7.04-11.03)	0.09(-0.06-0.25)	147(126-169)	221.9(189.62-258.15)	191(149-242)	216.87(169.63-273.62)	0.02(-0.12-0.15)
Dominican Republic	281(237-324)	7.28(6.14-8.41)	1071(763-1459)	11.31(8.13-15.28)	1.88(1.7-2.05)	212(180-243)	6(5.09-6.92)	707(521-938)	7.79(5.8-10.29)	1.39(1.2-1.59)	5639(4817-6454)	138.13(117.41-158.9)	17628(12548-24079)	183.41(131.21-249.33)	1.37(1.17-1.57)
Ecuador	242(215-310)	4.14(3.71-5.27)	1188(902-1568)	7.61(5.77-9.98)	2.42(2.13-2.71)	163(147-198)	3.11(2.8-3.79)	536(405-691)	3.73(2.81-4.76)	1.02(0.8-1.23)	4496(4077-5465)	75.43(68.36-91.59)	12772(9741-16568)	82.4(62.88-107.02)	0.57(0.37-0.78)
Egypt	1262(1078-1481)	3.5(3.06-4.16)	3967(2817-5426)	5.2(3.72-7.2)	1.38(1.25-1.52)	684(604-823)	2.28(2.01-2.85)	1582(1131-2260)	2.47(1.78-3.61)	0.46(0.27-0.64)	22393(19741-25665)	62.51(55.27-73.23)	49099(35153-67564)	65.83(47.19-92.33)	0.37(0.21-0.54)
El Salvador	188(142-209)	5.98(4.53-6.69)	329(245-436)	5.56(4.12-7.39)	-0.33(-0.62 to -0.04)	121(98-134)	4.1(3.29-4.54)	178(135-231)	2.96(2.24-3.85)	-1.23(-1.5 to -0.96)	3312(2700-3643)	103.9(83.87-114.61)	4244(3156-5578)	72.18(53.55-95.09)	-1.36(-1.66 to -1.06)
Equatorial Guinea	14(9-19)	6.28(4.36-8.83)	36(22-56)	6.7(4.34-10.14)	0.36(0.14-0.57)	12(8-17)	5.68(3.94-7.95)	24(15-37)	5.12(3.39-7.56)	-0.32(-0.54 to -0.1)	364(249-522)	157.17(108.73-223.55)	726(441-1150)	125.7(79.38-194.17)	-0.77(-1.04 to -0.5)
Eritrea	101(72-136)	7.73(5.63-10.24)	319(222-444)	9.27(6.7-12.49)	0.44(0.29-0.59)	82(59-110)	6.82(4.96-9.02)	226(160-308)	7.36(5.36-9.79)	0.07(-0.09-0.23)	2957(2103-3999)	208.05(149.7-277.96)	7958(5493-11003)	214.55(151.82-291.86)	-0.1(-0.25-0.06)
Estonia	291(264-320)	14.65(13.31-16.13)	349(264-454)	15.96(11.99-21)	0.09(-0.09-0.28)	158(145-172)	7.79(7.17-8.48)	144(109-184)	5.95(4.52-7.7)	-1.38(-1.6 to -1.16)	4614(4210-5056)	232.11(211.88-254.06)	3568(2691-4649)	164.55(124.02-215.21)	-1.76(-2 to -1.52)
Eswatini	29(22-37)	8.88(6.71-11.16)	64(44-88)	9.91(7-13.57)	0.48(0.12-0.84)	22(17-28)	7.31(5.56-9.14)	46(33-63)	7.75(5.57-10.46)	0.35(-0.08-0.78)	691(525-883)	199.53(150.69-253.97)	1453(992-2040)	214.82(149.72-297.76)	0.43(-0.05-0.91)
Ethiopia	2896(1730-4509)	11.43(7.28-17.29)	4775(3388-6370)	8.92(6.38-11.77)	-1.11(-1.31 to -0.91)	2027(1295-3082)	9.01(6-13.42)	2667(1886-3506)	6.06(4.28-7.95)	-1.57(-1.72 to -1.42)	70950(42878-110437)	270.16(171.15-412.75)	86750(61501-114429)	163.72(115.26-215.41)	-2(-2.15 to -1.84)
Fiji	39(27-50)	9.17(6.55-11.36)	73(49-96)	9.21(6.33-11.93)	0.03(-0.16-0.22)	20(15-24)	5.63(4.2-6.92)	36(26-46)	5.22(3.81-6.56)	-0.19(-0.38 to -0.01)	624(461-778)	142.19(105.3-175.77)	1035(741-1342)	127.83(92.58-163.55)	-0.27(-0.45 to -0.1)
Finland	637(585-693)	9.5(8.7-10.34)	1141(872-1470)	11.55(8.79-14.95)	0.9(0.74-1.07)	228(212-244)	3.26(3.03-3.49)	340(299-376)	2.85(2.54-3.14)	-0.31(-0.4 to -0.22)	5654(5261-6062)	84.67(78.78-90.85)	7254(6406-8129)	71.9(63.89-80.62)	-0.37(-0.47 to -0.28)
France	22596(21058-24184)	31.06(28.91-33.32)	21882(16518-28715)	20.19(15.11-26.69)	-1.62(-1.71 to -1.53)	9900(9394-10398)	13.12(12.47-13.77)	7285(6470-8099)	5.94(5.29-6.61)	-2.92(-3.03 to -2.8)	276242(262336-290708)	388.63(369.16-409.25)	179057(158566-201228)	166.48(147.52-188.3)	-3.16(-3.27 to -3.04)
Gabon	54(41-71)	9.2(7.03-11.93)	96(67-131)	8.45(6.01-11.29)	-0.36(-0.41 to -0.3)	43(33-57)	7.68(5.9-9.93)	69(49-93)	6.5(4.69-8.65)	-0.63(-0.67 to -0.58)	1265(944-1676)	207.77(155.74-273.64)	2005(1388-2762)	167.31(117.82-227.6)	-0.79(-0.85 to -0.74)
Gambia	12(9-16)	3.06(2.28-3.96)	42(31-56)	3.91(2.9-5.09)	0.67(0.49-0.85)	9(7-12)	2.52(1.91-3.22)	29(22-38)	3.01(2.27-3.87)	0.47(0.31-0.63)	282(200-377)	66.11(48.05-87.39)	889(639-1185)	78.87(57.42-103.85)	0.44(0.25-0.63)
Georgia	672(577-782)	10.55(9.08-12.25)	665(540-805)	12.11(9.81-14.67)	0.87(0.46-1.29)	456(396-529)	7.22(6.26-8.33)	429(352-516)	7.46(6.12-8.97)	0.62(0.2-1.04)	13454(11606-15667)	210.39(181.53-244.15)	11456(9312-13884)	211.62(172.65-256.34)	0.45(0.04-0.87)
Germany	17504(16319-18703)	15.72(14.59-16.85)	24071(18454-31544)	15.97(12.15-20.99)	-0.13(-0.22 to -0.04)	7478(7090-7817)	6.35(6.02-6.63)	8805(7944-9663)	5.08(4.6-5.58)	-0.87(-0.92 to -0.83)	213024(201517-223786)	191.48(180.84-201.4)	211874(190779-233825)	137.9(124.21-152.39)	-1.32(-1.37 to -1.26)
Ghana	209(161-264)	3.09(2.41-3.89)	592(455-756)	3.51(2.75-4.43)	0.47(0.43-0.51)	171(132-217)	2.73(2.13-3.43)	458(356-583)	2.95(2.32-3.69)	0.41(0.36-0.47)	5212(3987-6685)	70.5(54.5-89.74)	13073(9939-16882)	72.11(55.78-92.36)	0.15(0.1-0.19)
Greece	1678(1553-1811)	11.44(10.58-12.35)	2416(1866-3084)	12.48(9.54-16.09)	0.25(0.11-0.39)	707(665-750)	4.7(4.42-4.98)	913(828-990)	3.91(3.58-4.23)	-0.72(-0.78 to -0.66)	16263(15336-17231)	110.85(104.71-117.4)	18893(17236-20657)	97.98(89.4-107.23)	-0.46(-0.5 to -0.42)
Greenland	12(11-15)	30.81(26.49-35.83)	15(12-18)	20.21(16.02-24.72)	-1.69(-1.82 to -1.55)	9(8-11)	23.41(20.31-26.97)	10(7-12)	13.37(10.55-16.32)	-2.17(-2.28 to -2.05)	298(249-356)	675.19(574.77-791.03)	271(209-339)	355.08(276.38-440.17)	-2.38(-2.48 to -2.29)
Grenada	9(8-10)	12.56(11.09-14.17)	13(11-15)	11.28(9.72-13)	-0.23(-0.39 to -0.08)	6(6-7)	8.73(7.74-9.78)	8(7-9)	7(6.14-7.99)	-0.58(-0.72 to -0.44)	149(132-167)	224.86(198.72-253.54)	208(179-241)	175.73(151.83-202.6)	-0.6(-0.74 to -0.45)
Guam	7(6-9)	8.5(7.21-10.27)	20(15-25)	10.59(8.11-13.05)	0.91(0.73-1.08)	4(4-5)	5.79(4.89-6.9)	11(9-13)	5.74(4.49-7.05)	0.14(-0.1-0.37)	133(112-158)	146.96(124.9-174.5)	316(245-390)	164.77(128.55-203.13)	0.62(0.41-0.83)
Guatemala	189(166-217)	4.99(4.38-5.68)	559(436-706)	4.72(3.7-5.94)	-0.92(-1.16 to -0.67)	143(125-162)	4.23(3.73-4.75)	331(262-412)	3.08(2.47-3.81)	-1.83(-2.07 to -1.6)	4105(3598-4666)	99.2(87.04-112.45)	8598(6717-10863)	71.42(56-90.1)	-1.78(-1.98 to -1.58)
Guinea	231(190-278)	6.62(5.47-7.95)	503(377-658)	8.28(6.26-10.72)	0.98(0.92-1.05)	180(150-216)	5.47(4.54-6.51)	369(279-478)	6.59(5.03-8.45)	0.89(0.81-0.96)	5276(4338-6362)	145.23(119.35-174.71)	11146(8255-14709)	173.8(129.62-228.58)	0.88(0.8-0.95)
Guinea-Bissau	27(18-36)	5.98(4.04-7.93)	48(34-63)	5.88(4.22-7.63)	0.16(0.06-0.26)	22(14-29)	5.16(3.55-6.79)	36(26-48)	4.93(3.56-6.36)	0.07(-0.02-0.17)	667(442-904)	141.83(94.49-191.1)	1156(816-1540)	131.3(93.08-173.13)	-0.04(-0.13-0.06)
Guyana	28(24-33)	6.79(5.78-7.89)	48(36-62)	7(5.31-9.02)	0.13(0.03-0.24)	20(17-23)	5.18(4.45-5.98)	29(22-38)	4.62(3.55-5.86)	-0.26(-0.34 to -0.17)	575(486-673)	134.79(114.24-157.65)	868(646-1135)	123.89(93-160.57)	-0.12(-0.21 to -0.03)
Haiti	352(212-499)	10.09(6.14-14.14)	658(399-977)	8.8(5.4-12.96)	-0.33(-0.47 to -0.18)	292(178-411)	8.94(5.52-12.44)	506(309-750)	7.38(4.56-10.79)	-0.52(-0.65 to -0.39)	8760(5295-12448)	235.35(143.75-331.57)	14827(8946-22122)	186.28(112.91-277.04)	-0.64(-0.8 to -0.48)

Honduras	157(92-195)	6.76(3.94-8.34)	805(357-1154)	12.32(5.58-17.42)	2.12(1.96-2.28)	98(61-118)	4.71(2.91-5.7)	396(211-537)	6.81(3.64-9.14)	1.39(1.23-1.56)	2868(1856-3497)	120.92(76.46-147.28)	10334(5470-14404)	160.62(85.22-221.37)	1.06(0.94-1.19)
Hungary	3142(2935-3354)	22.77(21.27-24.3)	4002(3197-4942)	24.69(19.56-30.67)	-0.18(-0.66-0.3)	1897(1793-2005)	13.59(12.84-14.34)	2025(1636-2489)	11.94(9.58-14.74)	-0.84(-1.31 to -0.37)	57812(54455-61232)	426.31(401.17-452.05)	56496(45202-70048)	357.84(284.28-446.92)	-1.16(-1.68 to -0.64)
Iceland	46(41-52)	17.31(15.4-19.43)	77(65-90)	16.06(13.53-18.84)	-0.44(-0.58 to -0.29)	13(12-14)	4.58(4.16-5.02)	18(15-20)	3.24(2.83-3.69)	-1.32(-1.4 to -1.24)	328(298-360)	123.45(112.1-135.61)	428(373-489)	86.96(75.82-99.3)	-1.36(-1.45 to -1.27)
India	91406(79512-106546)	18.26(15.85-21.29)	236351(195079-281739)	19.66(16.23-23.34)	0.14(0.04-0.24)	72948(63355-85466)	15.7(13.55-18.39)	170252(141815-203484)	14.84(12.38-17.68)	-0.32(-0.42 to -0.23)	325677(2026470-2710039	425.54(370.25-496.55)	004100(4152244-6002192	402(334.13-481.84)	-0.29(-0.38 to -0.19)
Indonesia	8487(6731-10278)	7.64(6.13-9.14)	21243(15462-28402)	9.26(6.84-12.29)	0.66(0.61-0.7)	5799(4721-6935)	5.8(4.74-6.9)	12749(9560-17114)	6.19(4.69-8.22)	0.28(0.23-0.33)	183691(148126-220397)	155.28(126.11-185.79)	357720(267081-482848)	150.34(113.02-201.51)	-0.09(-0.14 to -0.03)
Iran (Islamic Republic of)	1974(1640-2244)	6.8(5.65-7.68)	6937(5363-7795)	8.67(6.87-9.71)	0.72(0.53-0.91)	1176(983-1307)	4.72(3.93-5.29)	2553(2267-2822)	3.65(3.24-4.03)	-0.89(-1.05 to -0.72)	34857(29150-38762)	116.06(97-128.89)	67759(59734-74855)	87.01(76.88-96)	-1(-1.16 to -0.84)
Iraq	665(512-847)	7.53(5.83-9.58)	2715(1961-3613)	9.78(7.23-12.67)	0.99(0.79-1.2)	416(326-525)	5.21(4.08-6.61)	1062(805-1337)	4.69(3.62-5.74)	-0.49(-0.57 to -0.41)	12683(9829-16046)	142.86(111.25-181.1)	31852(23496-41369)	119.39(89.57-151.73)	-0.78(-0.84 to -0.72)
Ireland	509(464-556)	12.92(11.79-14.14)	919(690-1208)	13.23(9.88-17.41)	0.21(0.1-0.31)	213(197-230)	5.32(4.93-5.72)	264(233-297)	3.57(3.16-4.01)	-1.45(-1.54 to -1.36)	5144(4765-5537)	132.41(122.6-142.63)	6224(5469-7067)	88.68(77.93-100.67)	-1.48(-1.56 to -1.4)
Israel	327(297-360)	6.92(6.28-7.61)	1008(769-1299)	9.42(7.17-12.18)	0.71(0.41-1.01)	153(140-167)	3.25(2.98-3.54)	329(291-364)	2.82(2.5-3.12)	-0.91(-1.12 to -0.71)	3624(3340-3941)	77(70.92-83.77)	7217(6434-8031)	66.71(59.45-74.25)	-0.94(-1.17 to -0.71)
Italy	16721(15695-17788)	20.64(19.31-21.98)	18256(14531-22503)	16.41(13.02-20.29)	-0.95(-1.07 to -0.83)	6480(6266-6662)	7.52(7.27-7.73)	5708(5161-6095)	4.15(3.82-4.41)	-2.17(-2.26 to -2.08)	166753(161075-172252)	203.8(196.78-210.7)	124082(114471-132657)	107.31(99.76-114.95)	-2.37(-2.46 to -2.28)
Jamaica	104(94-114)	5.84(5.29-6.44)	225(171-290)	7.53(5.71-9.7)	0.87(0.53-1.21)	69(63-76)	3.89(3.55-4.28)	122(95-154)	4.07(3.14-5.15)	0.12(-0.2-0.43)	1720(1567-1888)	98.45(89.53-108.59)	3166(2418-4058)	106.39(81.2-136.35)	0.16(-0.19-0.52)
Japan	15520(14705-16297)	9.29(8.8-9.76)	31425(25702-37613)	11.32(9.37-13.65)	0.77(0.54-0.99)	5040(4792-5186)	3.04(2.87-3.13)	11659(9831-12751)	3.11(2.75-3.35)	0.01(-0.19-0.21)	124186(119510-128495)	73.69(70.89-76.27)	207613(185612-224257)	73.04(67.31-78.4)	-0.15(-0.39-0.09)
Jordan	141(111-176)	8.36(6.71-10.29)	629(496-801)	7.86(6.26-10)	-0.62(-0.83 to -0.4)	71(57-86)	5.23(4.24-6.37)	193(155-243)	3.13(2.53-3.93)	-2.31(-2.58 to -2.04)	2259(1822-2753)	134.72(108.85-164.18)	5795(4642-7287)	75.07(60.22-94.59)	-2.66(-2.98 to -2.34)
Kazakhstan	1800(1609-2096)	13.17(11.81-15.3)	1963(1653-2333)	10.57(8.93-12.49)	-1.17(-1.37 to -0.98)	1146(1032-1309)	8.69(7.86-9.93)	1002(850-1179)	5.68(4.83-6.66)	-1.92(-2.11 to -1.73)	34991(31404-40230)	251.51(226.49-288.99)	28999(24453-34369)	153.4(130.06-181.33)	-2.23(-2.44 to -2.03)
Kenya	712(511-908)	7.46(5.35-9.46)	2612(1919-3386)	9.91(7.36-12.68)	1.15(0.98-1.33)	547(393-696)	6.17(4.43-7.83)	1982(1466-2576)	8.21(6.16-10.58)	1.33(1.11-1.55)	17980(12919-22894)	175.71(125.73-224.27)	65709(48235-85676)	231.12(170.84-300.36)	1.27(1.03-1.51)
Kiribati	8(7-10)	19.79(15.94-23.88)	17(13-22)	21.92(16.89-28.08)	0.54(0.42-0.66)	6(4-7)	14.54(11.84-17.55)	11(8-14)	15.45(12.04-19.6)	0.41(0.3-0.53)	188(148-232)	414.74(328.59-510.25)	357(266-468)	423.73(322.01-548.59)	0.26(0.16-0.37)
Kuwait	79(69-91)	8.86(7.73-10.04)	255(204-315)	7.47(6.04-9.2)	-0.45(-0.77 to -0.14)	25(22-28)	4(3.47-4.5)	59(47-71)	2.5(2.01-3.04)	-1.34(-1.6 to -1.07)	825(726-924)	100.15(87.65-112.3)	1679(1346-2068)	55.02(44.31-67.42)	-1.81(-2.09 to -1.53)
Kyrgyzstan	269(239-303)	8.41(7.49-9.45)	316(264-376)	6.23(5.25-7.37)	-0.91(-1.15 to -0.66)	184(164-206)	5.89(5.27-6.6)	181(154-214)	3.86(3.28-4.54)	-1.4(-1.6 to -1.21)	5588(4982-6283)	172.9(154.25-194.18)	5420(4573-6425)	103.46(87.77-122.29)	-1.76(-1.97 to -1.55)
People's Democratic Repu	261(175-359)	11.34(7.77-15.35)	458(319-616)	9.48(6.68-12.5)	-0.84(-0.98 to -0.7)	205(142-276)	9.48(6.67-12.64)	293(210-385)	6.78(4.94-8.75)	-1.38(-1.49 to -1.28)	6470(4342-8922)	265.65(181.77-362.99)	8703(6132-11649)	172.89(123.2-228.87)	-1.71(-1.82 to -1.6)
Latvia	420(386-458)	12.15(11.17-13.28)	446(348-574)	13.42(10.37-17.36)	0.24(-0.04-0.53)	281(260-306)	7.98(7.37-8.65)	254(199-322)	7.1(5.53-9.08)	-0.57(-0.87 to -0.26)	8324(7649-9080)	241.08(221.28-263.19)	6504(5000-8381)	200.91(153.98-259.75)	-0.91(-1.23 to -0.58)
Lebanon	298(228-377)	12.16(9.42-15.24)	973(733-1286)	18.35(13.88-24.32)	1.81(1.57-2.05)	171(134-214)	7.49(5.93-9.37)	295(237-382)	5.68(4.55-7.38)	-0.68(-0.86 to -0.5)	4903(3793-6119)	197.5(153.73-246.34)	7732(6064-10030)	148.24(116.2-192.38)	-0.74(-0.97 to -0.51)
Lesotho	77(58-101)	7.37(5.66-9.64)	151(105-208)	10.96(7.71-14.95)	1.72(1.52-1.92)	62(48-82)	6.23(4.81-8.1)	119(84-161)	9.06(6.48-12.16)	1.65(1.4-1.91)	1826(1387-2395)	169.12(129.12-221.7)	3669(2540-5068)	253.95(176.85-347.74)	1.82(1.52-2.11)
Liberia	48(37-60)	4.18(3.26-5.21)	95(67-129)	4.19(3.05-5.62)	0.2(0.01-0.38)	40(31-50)	3.67(2.9-4.55)	71(51-95)	3.48(2.56-4.63)	-0.01(-0.18-0.17)	1115(860-1413)	94.26(73.01-119.03)	2130(1475-2937)	86.48(61.4-117.63)	-0.16(-0.35-0.03)
Libya	249(182-322)	11.27(8.37-14.47)	913(653-1239)	14.39(10.6-19.16)	1.12(0.92-1.31)	150(113-190)	7.62(5.76-9.7)	369(282-479)	6.81(5.24-8.79)	-0.38(-0.51 to -0.26)	4732(3548-6054)	212.2(159.87-271.07)	11758(8811-15489)	189.44(143.89-247.54)	-0.38(-0.5 to -0.27)
Lithuania	610(562-662)	13.88(12.76-15.04)	654(512-824)	13.75(10.72-17.44)	-0.2(-0.47-0.08)	404(376-433)	9.03(8.41-9.67)	403(319-504)	7.92(6.24-9.95)	-0.57(-0.81 to -0.33)	12116(11195-13044)	275.17(254.07-296.45)	10793(8456-13662)	230.25(179.81-292.71)	-0.81(-1.08 to -0.54)
Luxembourg	107(97-118)	21.01(19.01-23.15)	149(119-187)	16.31(13.03-20.45)	-1.05(-1.25 to -0.85)	44(41-48)	8.42(7.77-9.11)	44(38-51)	4.47(3.85-5.24)	-2.33(-2.39 to -2.26)	1211(1112-1314)	238.8(219.44-258.86)	1135(968-1342)	123.07(105.18-145.45)	-2.47(-2.55 to -2.39)
Madagascar	435(345-540)	7.07(5.64-8.7)	944(669-1285)	6.71(4.85-9.05)	-0.23(-0.28 to -0.18)	331(265-405)	5.85(4.68-7.2)	652(467-886)	5.26(3.83-7.07)	-0.44(-0.5 to -0.37)	11460(9140-14080)	176.51(140.86-216.68)	22754(16182-31001)	151.6(108.53-205.58)	-0.57(-0.61 to -0.52)
Malawi	221(176-275)	4.66(3.8-5.61)	460(324-636)	4.96(3.69-6.53)	0.16(0.07-0.25)	145(118-174)	3.47(2.89-4.12)	266(201-346)	3.42(2.66-4.32)	-0.16(-0.26 to -0.06)	4907(3918-6009)	99.47(80.96-119.67)	8920(6406-12087)	95.03(70.74-124.52)	-0.31(-0.44 to -0.19)
Malaysia	1943(1643-2232)	18.12(15.49-20.74)	5518(4166-7131)	18.94(14.4-24.36)	-0.34(-0.63 to -0.04)	1356(1164-1542)	13.59(11.67-15.59)	2846(2183-3644)	10.48(8.09-13.37)	-1.47(-1.76 to -1.19)	44801(38039-50874)	393.02(335.12-447.78)	85943(65253-110655)	289.85(220.94-372.19)	-1.61(-1.96 to -1.27)
Maldives	10(7-13)	11.38(8.22-14.22)	37(30-46)	11.2(9.04-13.76)	-0.43(-0.62 to -0.24)	6(5-8)	8.3(6.03-10.32)	15(12-18)	5.53(4.44-6.74)	-1.9(-2.06 to -1.73)	184(128-237)	187.27(133.35-234.94)	367(297-450)	112.41(90.75-137.51)	-2.3(-2.52 to -2.09)
Mali	163(135-196)	3.55(2.96-4.25)	375(277-507)	3.73(2.84-4.92)	0.15(0.07-0.23)	127(106-153)	2.97(2.49-3.57)	258(196-343)	2.88(2.24-3.75)	-0.08(-0.17-0.01)	3974(3278-4786)	82.5(68.36-99.11)	8168(6027-11034)	78.16(58.89-104.67)	-0.19(-0.3 to -0.08)
Malta	60(53-68)	14.09(12.55-15.87)	106(87-129)	14.39(11.73-17.45)	0.06(-0.02-0.14)	26(24-29)	6.17(5.6-6.76)	33(29-38)	3.9(3.38-4.49)	-1.62(-1.67 to -1.58)	692(627-764)	162.89(147.69-179.49)	809(695-936)	108.56(93.12-125.51)	-1.41(-1.46 to -1.36)
Marshall Islands	2(1-2)	8.57(6.68-11.16)	4(3-5)	9.47(6.82-13.02)	0.38(0.29-0.46)	1(1-1)	6.68(5.18-8.65)	2(2-3)	6.72(4.84-9.14)	0.08(-0.02-0.17)	35(27-46)	178.8(138.02-234.17)	77(53-109)	180.36(128.16-250.97)	0.04(-0.05-0.12)
Mauritania	45(34-57)	4.25(3.27-5.38)	84(58-119)	3.89(2.73-5.37)	-0.12(-0.27-0.03)	36(28-45)	3.6(2.8-4.53)	61(43-85)	3.05(2.2-4.15)	-0.34(-0.48 to -0.19)	1032(795-1313)	94.41(72.74-120.13)	1645(1092-2409)	72.39(49.09-103.52)	-0.68(-0.83 to -0.53)
Mauritius	74(69-81)	9.37(8.68-10.12)	181(143-225)	10.37(8.21-12.85)	0.31(0.16-0.45)	42(39-45)	5.71(5.31-6.14)	90(72-112)	5.19(4.18-6.38)	-0.31(-0.45 to -0.18)	1206(1121-1299)	149.05(138.55-160.49)	2419(1916-3022)	136.51(108.47-169.83)	-0.27(-0.41 to -0.12)
Mexico	2595(2506-2685)	5.8(5.56-6.01)	7484(6317-8802)	6.23(5.27-7.31)	-0.02(-0.14-0.1)	1749(1674-1811)	4.33(4.1-4.49)	3625(3076-4216)	3.18(2.7-3.7)	-1.32(-1.43 to -1.2)	45180(43809-46663)	98.03(94.58-101.34)	88821(75457-103777)	73.97(62.9-86.21)	-1.23(-1.35 to -1.1)
cronesia (Federated States	5(3-6)	9.03(6.57-12.08)	8(5-11)	10.19(6.96-14.12)	0.37(0.31-0.44)	3(2-4)	6.92(5.09-9.17)	5(3-7)	6.64(4.67-9.03)	-0.18(-0.25 to -0.1)	104(74-143)	191.26(136.57-260.06)	147(95-213)	177.96(119.28-251.47)	-0.27(-0.35 to -0.19)
Monaco	13(10-16)	22.47(17.28-28.27)	16(12-20)	21.62(16.52-27.5)	-0.03(-0.11-0.05)	5(4-6)	7.4(5.77-9.13)	5(4-6)	5.46(4.29-6.68)	-1(-1.08 to -0.92)	113(87-140)	204.42(157.45-255.61)	111(87-138)	150.14(115.91-189.09)	-1.02(-1.11 to -0.92)
Mongolia	104(84-126)	9.57(7.71-11.59)	183(130-242)	7.62(5.4-9.91)	-1.42(-1.66 to -1.17)	77(62-93)	7.56(6.11-9.07)	119(86-155)	5.55(3.98-7.11)	-1.75(-2 to -1.5)	2108(1687-2555)	185.79(148.73-225.57)	3428(2469-4549)	132.77(96.41-174.21)	-1.84(-2.1 to -1.59)
Montenegro	104(90-121)	16(13.87-18.54)	172(139-212)	18.37(14.79-22.61)	0.64(0.55-0.74)	56(49-64)	8.71(7.62-10)	81(66-99)	8.38(6.79-10.27)	-0.07(-0.22-0.09)	1665(1442-1927)	252.82(219.51-291.46)	2195(1760-2717)	233.45(186.81-289.02)	-0.26(-0.44 to -0.08)
Morocco	1646(1219-2021)	10.51(7.87-12.79)	4566(3278-6131)	13.21(9.66-17.44)	0.67(0.53-0.81)	1156(858-1401)	8.02(5.9-9.77)	2441(1790-3122)	7.6(5.65-9.61)	-0.28(-0.42 to -0.13)	36834(27583-45001)	230.02(171.54-279.72)	72863(52611-96516)	208.31(152.57-271.65)	-0.42(-0.52 to -0.33)
Mozambique	296(232-375)	4.44(3.54-5.52)	870(610-1206)	6.64(4.79-8.98)	1.81(1.65-1.96)	238(189-297)	3.87(3.12-4.76)	590(430-790)	5.15(3.84-6.84)	1.39(1.24-1.53)	7307(5694-9226)	103.54(81.79-129.59)	18752(13322-25567)	139.95(101.53-188.59)	1.5(1.33-1.66)
Myanmar	2652(1809-3630)	10.31(7.22-13.89)	4582(3434-6026)	9.37(7.14-12.23)	-0.51(-0.58 to -0.44)	1975(1411-2649)	8.26(6.02-10.89)	2814(2198-3626)	6.2(4.91-7.9)	-1.13(-1.19 to -1.07)	62555(43232-84916)	231.4(163.7-312.38)	79317(60345-103686)	157.85(121.55-204.66)	-1.5(-1.58 to -1.43)
Namibia	81(59-103)	10.58(7.84-13.31)	223(164-300)	14.36(10.81-18.96)	1.05(0.87-1.22)	64(47-81)	8.71(6.52-10.93)	151(114-199)	10.33(7.96-13.35)	0.57(0.35-0.79)	1918(1377-2445)	243.67(176.86-310.47)	4642(3372-6288)	288.7(214.16-385.82)	0.54(0.27-0.81)
Nauru	1(0-1)	11.59(8.46-15.36)	1(0-1)	12.62(8.82-16.9)	0.14(0.04-0.24)	0(0-0)	7.71(5.7-10.09)	0(0-0)	7.22(5.23-9.44)	-0.24(-0.46 to -0.01)	11(7-15)	211.04(148.95-285.73)	12(8-16)	193.96(134.41-261.5)	-0.34(-0.58 to -0.1)
Nepal	1524(1096-2049)	14.38(10.33-19.29)	3712(2754-4864)	15.87(11.9-20.55)	0.35(0.09-0.61)	1235(891-1658)	12.45(8.94-16.65)	2726(2048-3536)	12.33(9.3-15.94)	0(-0.24-0.24)	39786(28771-53373)	349.83(253.12-468.9)	77072(56928-101588)	319.03(237.07-417.1)	-0.29(-0.55 to -0.02)
Netherlands	2229(2063-2402)	11.87(10.98-12.83)	4147(3157-5328)	13.94(10.62-17.97)	0.53(0.39-0.67)	815(764-861)	4.19(3.93-4.42)	1231(1096-1360)	3.69(3.31-4.07)	-0.58(-0.73 to -0.43)	20388(19137-21642)	109.52(102.72-116.32)	28069(25016-31284)	92.96(82.86-103.77)	-0.76(-0.95 to -0.58)
New Zealand	450(407-496)	11.68(10.6-12.84)	745(594-922)	10.69(8.48-13.27)	-0.31(-0.37 to -0.25)	162(149-176)	4.2(3.87-4.55)	240(213-264)	3.16(2.81-3.45)	-0.97(-1.02 to -0.93)	4111(3802-4464)	108.77(100.63-117.95)	5638(5045-6192)	81.99(73.33-89.68)	-1(-1.05 to -0.96)
Nicaragua	71(61-83)	4.3(3.7-5.07)	287(224-359)	6.21(4.87-7.67)	1.22(1.1-1.35)	51(44-59)	3.45(2.96-4.01)	151(120-184)	3.69(2.97-4.46)	-0.02(-0.2-0.16)	1383(1217-1585)	80.57(70.24-93.32)	3725(2917-4644)	80.68(63.6-99.71)	-0.22(-0.35 to -0.09)
Niger	89(66-115)	2.94(2.19-3.78)	256(175-351)	3.12(2.18-4.21)	0.38(0.29-0.48)	75(55-96)	2.65(1.99-3.39)	205(142-279)	2.74(1.94-3.64)	0.31(0.22-0.4)	2313(1691-2984)	69.09(50.71-89.03)	6044(4101-8327)	66.66(45.81-90.66)	0.01(-0.09-0.11)
Nigeria	1681(1218-2222)	3.58(2.61-4.7)	3553(2615-4746)	3.68(2.78-4.82)	0.28(0.17-0.39)	1475(1064-1949)	3.27(2.39-4.27)	2848(2107-3755)	3.21(2.42-4.15)	0.09(-0.01-0.2)	44592(31822-59753)	89.07(63.87-118.73)	87732(63624-118778)	82.17(60.65-108.61)	-0.16(-0.25 to -0.07)
Niue	0(0-0)	8.95(7.06-11.35)	0(0-0)	10.52(7.78-13.75)	0.49(0.42-0.57)	0(0-0)	5.41(4.36-6.72)	0(0-0)	4.99(3.88-6.25)	-0.37(-0.42 to -0.33)	3(2-4)	144.9(113.45-184.54)	3(2-3)	128.79(96.56-166.12)	-0.53(-0.58 to -0.49)
North Macedonia	200(170-225)	10.19(8.73-11.46)	389(295-505)	12.29(9.37-15.84)	0.38(0.12-0.63)	134(119-150)	7.06(6.31-7.9)	213(165-271)	6.8(5.32-8.57)	-0.38(-0.62 to -0.14)	3888(3434-4348)	193.23(171.69-216.13)	5677(4330-7310)	177.96(136.22-228.42)	-0.59(-0.83 to -0.34)
Northern Mariana Islands	4(3-5)	14.81(12.12-18.42)	13(10-16)	23.34(18.81-28.15)	2.21(1.79-2.64)	2(1-2)	8.99(7.49-10.84)	6(5-8)	12.21(10.15-14.29)	1.9(1.47-2.33)	62(47-80)	234.71(191.28-289.62)	185(148-227)	314.38(255.38-380.06)	1.83(1.41-2.25)
Norway	669(625-715)	11.21(10.46-11.98)	940(774-1130)	11.11(9.14-13.37)	0.01(-0.14-0.16)	239(228-248)	3.66(3.51-3.79)	250(227-270)	2.61(2.39-2.81)	-1.23(-1.31 to -1.16)	5551(5340-5765)	94.87(91.4-98.42)	5521(5092-5992)	64.36(59.54-69.8)	-1.41(-1.5 to -1.32)
Oman	52(37-70)	6.16(4.44-8.16)	212(162-277)	8.1(6.51-9.89)	1.11(0.76-1.46)	27(19-35)	3.83(2.78-5.05)	52(41-66)	3.16(2.63-3.8)	-0.38(-0.57 to -0.18)	865(613-1155)	100.94(72.14-133.78)	1731(1345-2292)	74.17(60.07-92.97)	-0.86(-1.14 to -0.59)
Pakistan	17733(14971-20902)	28.82(24.23-34.01)	47804(37608-61097)	36.42(29.05-46.25)	0.66(0.48-0.85)	13814(11683-16347)	23.51(19.82-27.85)	31919(25202-40727)	26.68(21.3-33.71)	0.27(0.05-0.49)	424466(358430-502479)	662.49(561.4-784.75)	1063643(835570-1362825	764.67(604.65-978.18)	0.32(0.08-0.56)
Palau	4(3-5)	35.92(27.89-46.2)	8(6-11)	37.4(28.8-47.65)	0.1(0.04-0.16)	2(1-2)	18.01(14.35-22.72)	3(3-4)	16.65(13.2-20.81)	-0.24(-0.31 to -0.18)	52(40-67)	468.36(364.85-599.91)	100(77-130)	434.77(339.04-560.6)	-0.22(-0.28 to -0.16)
Palestine	57(40-78)	5.91(4.18-8)	192(145-237)	6.66(5.09-8.1)	0.19(-0.13-0.51)	30(22-40)	3.6(2.61-4.73)	64(51-77)	2.91(2.33-3.48)	-0.9(-1.05 to -0.76)	824(587-1113)	86.71(62.06-116.2)	1863(1487-2247)	68.73(54.95-82.62)	-0.95(-1.1 to -0.81)
Panama	132(119-146)	8.35(7.52-9.23)	305(227-397)	7.31(5.46-9.53)	-0.46(-0.61 to -0.31)	84(76-92)	5.66(5.09-6.26)	151(114-195)	3.63(2.75-4.68)	-1.41(-1.58 to -1.24)	2155(1961-2368)	135.94(123.46-149.54)	3677(2751-4825)	88.29(66.1-115.7)	-1.36(-1.54 to -1.19)
Papua New Guinea	117(82-165)	5.6(3.95-7.85)	351(241-512)	6.36(4.49-9.14)	0.48(0.45-0.51)	84(59-118)	4.49(3.2-6.22)	231(162-335)	4.83(3.44-6.83)	0.34(0.3-0.38)	2732(1917-3856)	120.43(84.5-169.74)	7562(5182-11157)	127.17(89.12-183.73)	0.28(0.23-0.33)
Paraguay	140(119-166)	5.98(5.06-7.05)	513(368-684)	8.8(6.32-11.67)	1.05(0.89-1.2)	96(82-113)	4.33(3.68-5.1)	287(210-376)	5.15(3.76-6.72)	0.41(0.26-0.56)	2664(2253-3154)	111.33(93.98-131.75)	7806(5608-10365)	133.17(95.86-176.35)	0.36(0.21-0.52)
Peru	666(555-786)	5.15(4.3-6.09)	1853(1332-2512)	5.64(4.06-7.65)	0.41(0.25-0.57)	447(378-524)	3.78(3.2-4.44)	816(601-1083)	2.54(1.88-3.38)	-1.36(-1.56 to -1.16)	12346(10381-14471)	93.59(78.56-109.92)	19675(14320-26564)	60.42(44-81.49)	-1.51(-1.73 to -1.3)
Philippines	4420(3679-5092)	12.59(10.61-14.5)	9617(7605-12141)	11.08(8.83-13.86)	-0.76(-0.98 to -0.54)	2490(2136-2849)	8.24(7.14-9.42)	5030(4072-6140)	6.48(5.31-7.84)	-1.07(-1.25 to -0.88)	78989(67622-89296)	217.83(186.33-248.9)	149932(120724-183727)	169.97(137.61-207.29)	-1.13(-1.31 to -0.95)
Poland	6376(6050-6626)	14.83(14.07-15.42)	9577(7776-11898)	15.29(12.36-19.11)	-0.01(-0.08-0.06)	4164(4005-4274)	9.69(9.3-9.94)	5193(4239-6360)	7.81(6.36-9.59)	-0.86(-0.93 to -0.79)	119259(114612-122539)	278.29(267.19-286.09)	134246(108783-165442)	216.01(174.21-266.99)	-1(-1.08 to -0.93)
Portugal	2102(1942-2262)	15.92(14.72-17.13)	3602(2713-4675)	18.99(14.14-24.88)	0.53(0.38-0.67)	1103(1034-1169)	8.26(7.74-8.73)	1340(1202-1476)	6.44(5.76-7.1)	-0.91(-1.01 to -0.82)	29535(27700-31313)	228.08(213.73-242.24)	34396(30608-38291)	188.17(167.28-210.63)	-0.73(-0.89 to -0.57)
Puerto Rico	511(470-554)	14.2(13.05-15.4)	508(385-662)	8.42(6.32-11.1)	-1.82(-2.06 to -1.58)	309(285-332)	8.62(7.96-9.27)	253(193-324)	3.64(2.78-4.71)	-3.18(-3.44 to -2.92)	7608(7036-8194)	212.21(196.12-228.81)	5590(4212-7312)	92.34(69.01-121.53)	-3.07(-3.33 to -2.81)
Qatar	13(9-17)	9.96(7.1-12.7)	132(91-187)	11.32(7.98-15.22)	0.63(0.32-0.94)	7(5-9)	7.38(5.22-9.41)	34(23-47)	5.79(4.12-7.76)	-0.87(-1.16 to -0.57)	225(161-289)	163.02(114.95-209.52)	1135(778-1593)	105.91(74-143.39)	-1.62(-1.89 to -1.35)
Republic of Korea	2814(2469-3792)	8.54(7.56-11.28)	11020(8214-13801)	13.35(9.72-16.77)	1.78(0.88-2.68)	1362(1244-1629)	4.7(4.25-5.71)	2592(2176-2980)	2.95(2.48-3.38)	-2.13(-2.46 to -1.8)	39489(35596-47030)	117.79(106.65-140.77)	61349(51223-71271)	70.59(58.72-81.77)	-2.07(-2.38 to -1.76)
Republic of Moldova	555(508-607)	11.93(10.93-13.03)	718(603-851)	12.8(10.73-15.15)	0.31(0.06-0.55)	400(369-435)	8.68(8.02-9.42)	469(397-553)	8.14(6.89-9.6)	-0.18(-0.44-0.08)	12769(11733-13940)	273.62(251.55-298.78)	13957(11734-16510)	250.29(210.24-295.77)	-0.3(-0.57 to -0.03)
Romania	3175(2965-3383)	11.26(10.53-12)	6379(5072-7871)	20.37(16.09-25.24)	2.06(1.86-2.25)	2055(1940-2170)	7.27(6.88-7.67)	3439(2763-4183)	10.45(8.31-12.76)	1.25(1.06-1.44)	63165(59253-66925)	224.52(211.05-237.73)	98098(77801-120451)	319.71(252.07-395.02)	1.18(0.97-1.4)
Russian Federation	21779(20941-22945)	11.93(11.46-12.6)	31472(26277-37449)	14.45(12.06-17.24)	0.41(0.16-0.66)	14111(13612-14708)	7.72(7.44-8.06)	14709(12322-17426)	6.4(5.36-7.58)	-1.24(-1.56 to -0.93)	420082(403956-440612)	229.37(220.36-240.99)	413860(344821-491785)	187.56(156.23-223)	-1.32(-1.65 to -0.98)
Rwanda	363(266-475)	10.71(8-13.75)	628(455-852)	8.66(6.51-11.37)	-1.39(-1.66 to -1.12)	292(220-373)	9.22(7.04-11.67)	420(312-559)	6.52(4.98-8.45)	-1.94(-2.22 to -1.65)	9808(7211-12676)	275.14(204.84-354.97)	13747(9884-18747)	181.81(133.96-242.37)	-2.26(-2.58 to -1.94)
Saint Kitts and Nevis	5(4-5)	12.94(11.64-14.4)	9(7-11)	12.41(9.7-15.2)	-0.39(-0.62 to -0.17)	3(3-3)	8.45(7.66-9.34)	5(4-5)	6.89(5.78-8.1)	-0.84(-1.08 to -0.61)	74(66-82)	216.03(194.17-240.54)	126(98-154)	171.89(135.76-208.84)	-1.09(-1.35 to -0.83)
Saint Lucia	12(11-13)	13.16(11.98-14.4)	27(22-32)	12.4(10.29-14.87)	-0.36(-0.6 to -0.12)	8(7-9)	9.38(8.61-10.19)	16(13-19)	7.5(6.28-8.88)	-0.97(-1.24 to -0.7)	207(189-226)	233.52(213.34-255.04)	415(342-499)	188.7(156-226.48)	-0.85(-1.1 to -0.6)
nt Vincent and the Grenadi	10(9-11)	13.67(12.34-15.09)	21(18-24)	15.15(12.98-17.61)	0.33(0.11-0.55)	7(6-8)	9.63(8.74-10.58)	13(12-15)	9.94(8.64-11.47)	0.1(-0.15-0.36)	173(157-191)	240.19(216.84-265.95)	345(295-404)	250.62(214.82-292.43)	0.12(-0.1-0.34)
Samoa	9(7-11)	8.75(6.79-11.19)	14(10-19)	8.69(6.36-11.68)	-0.18(-0.24 to -0.12)	5(4-7)	5.82(4.65-7.4)	7(6-9)	4.81(3.77-6.3)	-0.68(-0.75 to -0.61)	164(126-210)	165.49(128.51-211.53)	226(167-305)	137.55(102.69-183.33)	-0.67(-0.73 to -0.61)
San Marino	6(5-7)	18.58(15.19-22.24)	10(8-14)	19.5(14.16-26.51)	0.21(0.11-0.31)	2(2-2)	6.14(5.01-7.39)	3(2-4)	4.94(3.18-7.28)	-0.71(-0.85 to -0.56)	50(40-60)	161.55(130.34-197.34)	71(45-105)	130.25(81.6-195.86)	-0.74(-0.89 to -0.6)
Sao Tome and Principe	2(1-2)	2.43(1.88-2.99)	4(3-6)	3.56(2.71-4.64)	1.3(1.22-1.37)	1(1-2)	2.11(1.68-2.58)	3(2-4)	2.77(2.15-3.59)	0.94(0.86-1.02)	38(28-47)	52.26(39.63-64.23)	82(61-108)	67.08(50.79-87.68)	0.84(0.73-0.94)
Saudi Arabia	416(303-555)	5.5(4.08-7.3)	3776(2655-5196)	11.71(8.79-15.28)	2.88(2.77-3)	261(188-350)	4.16(3.04-5.59)	715(543-933)	3.49(2.76-4.4)	-0.76(-0.9 to -0.62)	8609(6098-11629)	111.2(79.49-150.32)	26934(19852-36244)	96.05(74.19-123.79)	-0.63(-0.72 to -0.54)
Senegal	137(102-171)	3.98(3.01-4.9)	349(254-462)	4.41(3.28-5.75)	0.6(0.4-0.8)	110(83-136)	3.44(2.63-4.2)	269(199-351)	3.66(2.75-4.71)	0.5(0.31-0.69)	3219(2397-4016)	88.54(66.13-110.16)	7592(5422-10218)	90.4(65.76-119.72)	0.34(0.13-0.55)
Serbia	1669(1383-1922)	13.9(11.61-15.88)	2468(1889-3165)	17.21(13.1-22.2)	0.67(0.54-0.8)	1089(903-1249)	9.19(7.67-10.48)	1227(947-1554)	8.19(6.32-10.36)	-0.54(-0.69 to -0.4)	31751(26349-36629)	261.6(218.29-300.28)	31603(24116-40530)	224.16(170.35-288.4)	-0.73(-0.89 to -0.57)
Seychelles	12(10-14)	21.41(18.18-24.8)	33(27-40)	28.72(23.95-34.44)	0.72(0.55-0.89)	9(8-10)	15.97(13.56-18.49)	20(17-24)	18.39(15.44-21.96)	0.27(0.12-0.42)	236(200-275)	422.39(356.12-493.48)	562(462-681)	471.38(390.02-568.34)	0.09(-0.06-0.24)

Sierra Leone	79(58-101)	3.99(2.96-5.11)	170(123-229)	4.39(3.24-5.85)	0.6(0.46-0.73)	67(50-85)	3.51(2.64-4.45)	130(96-174)	3.67(2.75-4.84)	0.45(0.31-0.58)	1839(1344-2377)	89.83(65.78-115.62)	3821(2746-5228)	92.54(67.39-125.28)	0.39(0.26-0.51)
Singapore	693(609-795)	25.94(23.02-29.47)	1539(1175-2007)	19.07(14.65-24.74)	-1.18(-1.33 to -1.03)	266(248-285)	11.08(10.28-11.9)	328(286-376)	4.18(3.64-4.79)	-3.63(-3.78 to -3.47)	8782(8154-9441)	323.94(301.3-347.88)	9232(8026-10706)	114.24(99.65-132.03)	-3.89(-4.06 to -3.72)
Slovakia	1305(1183-1422)	22.56(20.44-24.61)	1638(1232-2146)	19.02(14.28-24.93)	-0.59(-0.68 to -0.5)	776(711-840)	13.4(12.3-14.51)	849(635-1106)	9.6(7.2-12.5)	-1.13(-1.23 to -1.04)	23716(21646-25764)	415.44(378.61-452.04)	23976(17749-31469)	282.38(208.41-371.77)	-1.38(-1.48 to -1.28)
Slovenia	454(337-598)	18.74(13.95-24.66)	598(449-797)	16.24(12.13-21.78)	-0.6(-0.69 to -0.52)	227(171-294)	9.33(7.04-12.09)	238(181-315)	6(4.53-7.96)	-1.75(-1.87 to -1.63)	6645(4936-8736)	274.87(203.63-362.24)	6054(4557-8096)	167.4(125.5-222.89)	-1.99(-2.13 to -1.86)
Solomon Islands	16(9-24)	9.46(5.8-14.07)	45(25-67)	11.42(7.01-16.63)	0.65(0.59-0.72)	10(6-16)	7.12(4.55-10.51)	25(15-37)	7.45(4.83-10.68)	0.24(0.19-0.29)	361(207-555)	207.32(124-314.24)	877(489-1331)	214.72(128.1-317.01)	0.22(0.17-0.27)
Somalia	248(168-359)	7.73(5.33-10.94)	604(380-901)	7.2(4.62-10.62)	-0.13(-0.17 to -0.09)	201(136-288)	6.78(4.7-9.52)	470(305-698)	6.21(4.06-9.1)	-0.22(-0.24 to -0.19)	7122(4789-10328)	202.03(137.7-289.16)	16671(10661-24918)	181.73(117.26-270.31)	-0.31(-0.33 to -0.29)
South Africa	1924(1701-2262)	8.47(7.42-10.03)	3363(3005-3861)	7.15(6.41-8.13)	-0.88(-1.23 to -0.53)	1349(1182-1598)	6.3(5.47-7.51)	2300(2069-2590)	5.14(4.63-5.76)	-0.98(-1.41 to -0.54)	41636(37057-48607)	176.57(155.94-208.28)	64801(57932-73725)	133.33(119.4-151.43)	-1.25(-1.7 to -0.8)
South Sudan	192(126-285)	7.19(4.77-10.58)	290(184-446)	6.39(4.18-9.59)	-0.41(-0.49 to -0.34)	158(104-233)	6.29(4.2-9.19)	222(140-341)	5.38(3.49-8)	-0.57(-0.63 to -0.51)	5005(3230-7499)	177.93(115.39-265.08)	7228(4430-11395)	147.38(92.49-227.27)	-0.7(-0.78 to -0.61)
Spain	12031(11265-12868)	23.96(22.39-25.67)	15851(12041-20514)	19.64(14.81-25.52)	-0.96(-1.05 to -0.87)	4658(4432-4880)	8.98(8.55-9.4)	4625(4146-5084)	5.12(4.61-5.63)	-2.22(-2.34 to -2.1)	128474(122113-134850)	260.62(247.37-273.6)	109946(98300-122172)	137.4(122.76-153.12)	-2.6(-2.73 to -2.47)
Sri Lanka	1512(1301-1732)	13.35(11.61-15.21)	4076(2927-5545)	15.88(11.46-21.44)	0.51(0.33-0.68)	921(807-1049)	9.08(7.95-10.32)	1957(1411-2623)	7.87(5.75-10.45)	-0.48(-0.69 to -0.27)	24936(21686-28462)	213.06(186.21-242.83)	48650(34635-66182)	186.27(133.41-251.61)	-0.44(-0.64 to -0.24)
Sudan	531(330-747)	5.19(3.29-7.2)	1388(891-1981)	6.17(4.11-8.57)	0.54(0.46-0.63)	393(251-538)	4.19(2.69-5.67)	716(495-973)	3.86(2.72-5.14)	-0.28(-0.31 to -0.25)	11769(7467-16430)	111.3(69.94-154.24)	21340(14177-29934)	97.26(66.22-133.93)	-0.47(-0.5 to -0.45)
Suriname	16(14-18)	5.78(5-6.52)	41(33-51)	6.71(5.38-8.27)	0.47(0.23-0.71)	11(10-13)	4.31(3.81-4.8)	26(21-32)	4.39(3.56-5.36)	0.07(-0.16-0.3)	313(268-353)	110.74(95.64-124.29)	722(576-895)	114.92(92-141.33)	0.09(-0.13-0.3)
Sweden	1184(1100-1273)	8.81(8.19-9.5)	1588(1309-1891)	8.88(7.33-10.61)	0.17(0.1-0.24)	463(430-495)	3.12(2.91-3.32)	543(489-594)	2.58(2.34-2.81)	-0.47(-0.58 to -0.37)	9958(9327-10620)	75.51(70.84-80.48)	10911(9970-11894)	60.81(55.56-66.17)	-0.56(-0.65 to -0.46)
Switzerland	1102(1010-1212)	11.8(10.8-13.01)	2112(1591-2771)	14.23(10.68-18.72)	0.37(-0.02-0.76)	397(371-427)	4(3.75-4.31)	647(567-722)	3.83(3.38-4.27)	-0.3(-0.56 to -0.04)	10084(9434-10889)	109(101.79-117.82)	14705(12900-16530)	98.07(86.15-110.36)	-0.62(-0.91 to -0.33)
Syrian Arab Republic	174(131-220)	2.96(2.22-3.75)	472(338-640)	3.64(2.64-4.87)	0.57(0.41-0.73)	115(88-144)	2.2(1.68-2.74)	224(165-300)	1.96(1.47-2.56)	-0.69(-0.88 to -0.5)	3434(2632-4332)	55.62(42.41-70.27)	6258(4555-8440)	46.9(34.5-62.71)	-0.87(-1.06 to -0.67)
Taiwan (Province of China	3555(3321-3812)	19.58(18.36-20.93)	13328(10065-17770)	36.69(27.67-48.92)	2.26(1.97-2.56)	1708(1634-1786)	10.07(9.62-10.53)	4577(3493-6076)	11.94(9.14-15.86)	0.59(0.39-0.79)	57529(54907-60242)	315.67(301.51-330.56)	139374(105544-186423)	378.21(286.17-506.19)	0.63(0.41-0.85)
Tajikistan	144(120-174)	4.87(3.98-6)	226(176-292)	4.27(3.35-5.4)	-0.48(-0.67 to -0.29)	117(95-143)	4.06(3.27-5.05)	166(129-213)	3.49(2.72-4.4)	-0.51(-0.66 to -0.35)	3492(2978-4108)	114.22(96.09-136.2)	5122(3969-6672)	85.82(66.87-109.68)	-1.15(-1.33 to -0.97)
Thailand	5152(4428-5982)	13.24(11.42-15.39)	12762(9146-17906)	12.77(9.19-17.95)	-0.66(-0.85 to -0.47)	3126(2692-3597)	8.95(7.7-10.28)	6421(4738-8490)	6.37(4.72-8.4)	-1.63(-1.79 to -1.47)	93354(80492-107380)	227.6(196.29-262.21)	161129(116100-217948)	159.07(115.11-213.83)	-1.75(-1.93 to -1.56)
Timor-Leste	24(17-32)	7.17(5.24-9.35)	73(49-99)	8.62(5.93-11.55)	0.68(0.4-0.96)	18(13-23)	5.93(4.39-7.69)	49(34-66)	6.13(4.35-8.13)	0.11(-0.12-0.34)	589(419-784)	157.05(113.93-204.94)	1323(863-1808)	151.17(101.63-205.23)	-0.18(-0.47-0.11)
Togo	60(46-75)	4.28(3.38-5.31)	196(142-263)	4.82(3.6-6.32)	0.52(0.46-0.57)	45(36-56)	3.56(2.84-4.37)	143(105-191)	3.89(2.95-5.03)	0.43(0.35-0.51)	1395(1096-1749)	92.91(73.17-116)	4428(3175-5984)	100.4(73.31-133.99)	0.37(0.3-0.44)
Tokelau	0(0-0)	7.13(5.48-9.26)	0(0-0)	8.1(5.85-10.84)	0.44(0.42-0.47)	0(0-0)	4.88(3.87-6.15)	0(0-0)	4.32(3.31-5.56)	-0.39(-0.41 to -0.36)	2(1-2)	128.61(98.3-166.83)	2(1-2)	111.37(82.05-148.48)	-0.48(-0.5 to -0.47)
Tonga	3(2-4)	5.51(4.22-6.98)	5(4-7)	6.61(4.98-8.74)	0.59(0.44-0.75)	2(2-3)	3.85(2.99-4.84)	3(2-4)	4.14(3.18-5.35)	0.27(0.12-0.42)	59(45-75)	97.44(74.52-123.63)	85(64-114)	104.2(78.04-138.65)	0.26(0.13-0.39)
Trinidad and Tobago	67(62-72)	7.61(7.04-8.23)	116(85-154)	6.41(4.71-8.51)	-1.03(-1.23 to -0.82)	44(41-48)	5.35(4.95-5.75)	68(51-88)	3.7(2.78-4.8)	-1.74(-1.97 to -1.51)	1217(1128-1313)	137.32(127.16-148.19)	1777(1304-2364)	97.49(71.61-129.67)	-1.65(-1.87 to -1.43)
Tunisia	615(495-755)	11.02(8.92-13.44)	2065(1445-2887)	15.83(11.13-22.04)	1.15(1.09-1.21)	380(308-462)	7.45(6.05-9.1)	802(574-1104)	6.36(4.58-8.69)	-0.73(-0.81 to -0.65)	11305(9203-13725)	200.53(163.21-243.49)	22703(16086-31433)	172.22(122.55-237.55)	-0.73(-0.81 to -0.64)
Turkey	3250(2431-4035)	8.24(6.2-10.11)	8164(6310-10482)	9(6.98-11.52)	0.44(0.29-0.59)	2163(1642-2645)	5.9(4.51-7.14)	3113(2445-3950)	3.57(2.81-4.53)	-1.76(-1.9 to -1.62)	64884(48655-80152)	159.53(119.86-195.96)	81310(63110-103289)	89.51(69.59-113.36)	-2.15(-2.29 to -2.02)
Turkmenistan	185(171-201)	8.65(8.01-9.38)	357(280-454)	8.11(6.39-10.27)	-0.28(-0.5 to -0.07)	131(122-142)	6.47(6.01-6.98)	204(161-258)	4.94(3.92-6.23)	-1.2(-1.38 to -1.02)	4200(3884-4545)	187.39(173.9-202.46)	6577(5165-8369)	144.02(113.63-182.69)	-1.13(-1.32 to -0.94)
Tuvalu	1(0-1)	8.69(6.5-11.57)	1(1-1)	8.94(6.44-12.32)	0.04(0.01-0.07)	0(0-1)	6.4(4.82-8.5)	1(0-1)	5.69(4.14-7.84)	-0.4(-0.45 to -0.35)	13(10-18)	175.26(129.93-236.61)	16(12-23)	151.67(107.72-215.24)	-0.46(-0.53 to -0.39)
Uganda	664(516-834)	8.91(7.05-11.01)	1976(1440-2593)	11.05(8.31-14.08)	0.51(0.36-0.66)	556(438-690)	7.91(6.33-9.7)	1375(1031-1755)	8.68(6.63-10.87)	0.08(-0.07-0.23)	18276(14090-23055)	230.21(179.59-286.96)	47382(34736-62354)	250.74(187.09-321.87)	-0.04(-0.23-0.15)
Ukraine	9198(8051-10672)	13.13(11.5-15.18)	12660(10162-15638)	18.61(14.92-23.04)	0.82(0.56-1.07)	5732(5028-6537)	8.04(7.08-9.13)	6577(5242-8115)	9.21(7.33-11.38)	-0.21(-0.52-0.1)	175187(152949-200638)	251.13(219.99-286.92)	197599(156628-245036)	291.25(230.67-361.55)	-0.2(-0.55-0.15)
United Arab Emirates	75(48-108)	10.77(5.93-15.21)	926(525-1491)	11.64(7.21-17.49)	0.06(-0.21-0.32)	40(24-57)	7.78(4.15-11.02)	325(195-521)	6.32(4.07-9.33)	-0.83(-1.13 to -0.52)	1528(982-2226)	198.82(113.99-282.86)	12869(7592-21039)	163.72(103.67-248.39)	-0.82(-1.03 to -0.6)
United Kingdom	10497(10086-10805)	12.85(12.37-13.22)	17481(14338-21163)	16.21(13.25-19.7)	0.78(0.72-0.84)	3816(3661-3916)	4.38(4.21-4.49)	4917(4607-5137)	4.06(3.84-4.23)	-0.38(-0.45 to -0.3)	91542(88511-94261)	113.74(110.18-117.05)	113524(108082-118850)	105.74(101.03-110.71)	-0.38(-0.44 to -0.31)
United Republic of Tanzani	1063(829-1395)	8.49(6.71-11.05)	2584(1871-3575)	8.8(6.56-11.87)	0.14(0.08-0.2)	823(646-1076)	7.08(5.64-9.18)	1750(1288-2387)	6.65(5.02-8.9)	-0.2(-0.24 to -0.16)	26541(20519-34930)	202.14(157.19-265.32)	56850(40589-79869)	186.27(135.76-256.31)	-0.27(-0.31 to -0.23)
United States of America	54052(52386-55550)	18.19(17.63-18.68)	90325(76559-106367)	17.79(15.08-20.97)	-0.19(-0.25 to -0.12)	14387(13823-14788)	4.65(4.48-4.77)	19458(18329-20298)	3.51(3.32-3.66)	-1.07(-1.19 to -0.96)	372835(359975-385591)	127.36(123.08-131.69)	475053(450032-499012)	92.43(87.59-97.17)	-1.22(-1.33 to -1.11)
United States Virgin Islands	8(7-10)	9.08(7.46-10.88)	21(17-25)	11.68(9.37-14.13)	1.41(1.12-1.7)	5(4-6)	6.43(5.32-7.64)	13(11-16)	7.22(5.93-8.56)	0.9(0.6-1.21)	152(125-183)	162.37(133.53-194.01)	320(257-386)	181.99(145.33-220.76)	0.88(0.57-1.18)
Uruguay	577(531-628)	15.32(14.1-16.69)	637(488-817)	13.26(10.1-17.12)	-0.54(-0.61 to -0.47)	378(350-407)	9.87(9.18-10.63)	350(314-388)	6.76(6.08-7.5)	-1.35(-1.44 to -1.27)	9942(9200-10739)	268.14(247.75-289.84)	8378(7484-9308)	177.2(158.02-197.13)	-1.5(-1.59 to -1.42)
Uzbekistan	751(653-924)	6.02(5.21-7.53)	2091(1724-2509)	8.58(7.16-10.12)	1.06(0.76-1.37)	555(483-683)	4.64(4.02-5.79)	1290(1068-1543)	5.92(4.99-6.95)	0.7(0.33-1.08)	17892(15731-21345)	137.73(120.68-165.88)	42840(35410-51459)	160.84(133.83-191.37)	0.29(-0.05-0.64)
Vanuatu	5(3-7)	6.71(4.61-9.59)	15(10-21)	7.82(5.33-11.1)	0.3(0.16-0.45)	4(2-5)	5.37(3.73-7.6)	10(7-14)	5.8(4.02-8.11)	0.11(0-0.22)	110(73-162)	141.66(95.97-204.97)	303(204-438)	154.17(105.08-221.6)	0.09(-0.05-0.22)
ezuela (Bolivarian Republi	739(692-790)	7.34(6.87-7.85)	2664(1965-3510)	8.98(6.66-11.76)	0.55(0.36-0.75)	522(490-554)	5.55(5.18-5.9)	1485(1111-1930)	5.14(3.88-6.66)	-0.48(-0.66 to -0.3)	14045(13245-14907)	134.86(126.95-143.2)	37878(27764-50019)	126.07(92.89-165.87)	-0.43(-0.6 to -0.26)
Viet Nam	6228(4814-7894)	14.48(11.25-18.4)	24695(17676-32294)	23.91(17.46-30.91)	2.04(1.89-2.19)	4416(3442-5650)	10.77(8.42-13.79)	11691(8872-14978)	12.07(9.3-15.29)	0.56(0.49-0.63)	133884(102933-170915)	308.17(237.47-395.59)	355251(262912-463244)	338.3(253.7-437.15)	0.52(0.44-0.61)
Yemen	315(201-459)	5.7(3.72-8.1)	1030(695-1457)	6.6(4.59-9.19)	0.65(0.57-0.73)	234(153-331)	4.65(3.09-6.48)	608(425-839)	4.58(3.27-6.26)	-0.04(-0.09-0.01)	7279(4601-10489)	125.03(81.31-177.76)	18445(12550-26009)	117.16(81.4-162.84)	-0.24(-0.3 to -0.19)
Zambia	328(255-417)	9.49(7.49-11.9)	917(656-1259)	10.52(7.75-14.06)	0.12(0.01-0.23)	254(200-318)	8.07(6.44-10.02)	587(431-785)	7.79(5.85-10.24)	-0.35(-0.51 to -0.19)	8585(6690-10909)	233.29(183.29-292.88)	20417(14612-27785)	225.37(164.1-302.72)	-0.38(-0.56 to -0.21)
Zimbabwe	354(291-418)	7.72(6.46-9.03)	769(554-1007)	9.29(6.97-11.91)	0.78(0.6-0.97)	245(207-285)	5.87(5.01-6.79)	508(386-649)	6.85(5.33-8.58)	0.8(0.62-0.97)	7493(6224-8767)	157.69(132.19-184.04)	16689(12349-21822)	193.88(146.39-248.68)	1.06(0.84-1.29)

Table S2 Global Burden and Trends of Five Subtypes of Head and Neck Cancer from 1990 to 2019 by gender, Related to Figure 5

HNC subtypes	Gender	1990		2019			1990		2019			1990		2019		
		Incidence cases	ASIR	Incidence cases	ASIR	EAPC	Death cases	ASDR	Death cases	ASDR	EAPC	DALYs cases	Age_standardised DALYs	DALYs cases	Age_standardised DALYs	EAPC
		(95%UI)	pre 100,000(95%UI)	(95%UI)	pre 100,000(95%UI)	(95%CI)	(95%UI)	pre 100,000(95%UI)	(95%UI)	pre 100,000(95%UI)	(95%CI)	(95%UI)	pre 100,000(95%UI)	(95%UI)	pre 100,000(95%UI)	(95%CI)
	Both	124643(119744-129440)	3.06(2.93-3.17)	209149(193876-224620)	2.51(2.32-2.69)	-0.86(-0.95to-0.77)	87459(83182-91551)	2.19(2.08-2.29)	123356(114941-132798)	1.49(1.39-1.61)	-1.5(-1.57to-1.42)	473141(2350349-259269	59.03(56.13-61.86)	262221(3034634-351135	38.83(36.13-41.78)	-1.65(-1.74to-1.57)
Larynx cancer	Female	15792(14728-16733)	0.73(0.68-0.78)	28529(26099-31304)	0.65(0.6-0.72)	-0.49(-0.54to-0.43)	11529(10663-12280)	0.54(0.5-0.58)	17800(16184-19688)	0.41(0.37-0.45)	-1.07(-1.13to-1.01)	318687(293330-340426)	14.54(13.42-15.53)	463837(421155-511948)	10.72(9.74-11.84)	-1.16(-1.23to-1.09)
	Male	108851(104479-113455)	5.73(5.5-5.97)	180620(166205-195786)	4.58(4.22-4.96)	-0.95(-1.05to-0.86)	75930(72032-79651)	4.15(3.94-4.35)	105555(97755-114522)	2.74(2.54-2.98)	-1.61(-1.69to-1.54)	154454(2039200-226591	107.97(102.33-113.45)	798385(2587372-303461	69.34(64.14-75.22)	-1.74(-1.83to-1.65)
	Both	175626(167516-184913)	4.28(4.07-4.51)	373098(340884-403866)	4.52(4.13-4.89)	0.14(0.1-0.18)	96628(90592-103050)	2.44(2.28-2.6)	199398(181651-218059)	2.44(2.22-2.66)	-0.05(-0.08to-0.02)	854782(2680931-305260	67.01(62.93-71.57)	506652(5004325-603342	66.05(60.06-72.35)	-0.12(-0.15to-0.08)
Lip and oral cavity cancer	Female	54969(51324-58495)	2.54(2.37-2.7)	129906(117073-142959)	3.01(2.71-3.31)	0.46(0.4-0.52)	29633(27302-31844)	1.41(1.3-1.52)	67837(60782-75653)	1.56(1.4-1.74)	0.21(0.15-0.28)	826951(759909-893271)	37.41(34.45-40.33)	747287(1564229-195156	40.78(36.48-45.55)	0.14(0.06-0.21)
	Male	120657(112725-129492)	6.24(5.84-6.7)	243192(218655-268264)	6.16(5.55-6.79)	-0.04(-0.1-0.01)	66995(61238-73050)	3.63(3.32-3.95)	131561(117700-145460)	3.42(3.06-3.77)	-0.22(-0.26to-0.19)	027832(1850001-221648	98.85(90.26-107.85)	759365(3330029-418123	92.84(82.41-103.07)	-0.24(-0.28to-0.2)
	Both	67518(61729-72995)	1.55(1.42-1.67)	176502(156046-199917)	2.12(1.87-2.4)	1.13(0.97-1.29)	53459(48875-57906)	1.26(1.15-1.36)	71610(65442-77625)	0.86(0.79-0.93)	-1.48(-1.56to-1.39)	880702(1715976-205061	41.67(38.08-45.35)	335096(2139753-253665	27.98(25.65-30.37)	-1.58(-1.67to-1.48)
Nasopharynx cancer	Female	23571(20320-26149)	1.05(0.91-1.16)	49220(42598-56983)	1.16(1-1.34)	0.27(0.15-0.39)	18347(15569-20725)	0.83(0.71-0.94)	20388(18156-22777)	0.47(0.42-0.53)	-2.24(-2.39to-2.09)	639939(535699-724874)	27.8(23.33-31.45)	650860(580292-726363)	15.39(13.73-17.16)	-2.4(-2.57to-2.22)
	Male	43947(39621-48687)	2.09(1.88-2.3)	127282(108042-148521)	3.14(2.67-3.65)	1.51(1.32-1.69)	35112(31341-38916)	1.72(1.55-1.9)	51223(45965-56951)	1.28(1.15-1.43)	-1.15(-1.21to-1.09)	240764(1105920-138283	56.09(50.02-62.33)	684236(1514430-186390	41.09(36.98-45.44)	-1.21(-1.27to-1.15)
	Both	66531(62626-71527)	1.6(1.5-1.72)	166901(152960-180359)	1.99(1.83-2.15)	0.76(0.72-0.79)	51460(47974-56457)	1.25(1.17-1.37)	114207(103154-126039)	1.37(1.24-1.51)	0.25(0.21-0.29)	556062(1446795-170587	36.37(33.84-39.82)	234593(2904981-357183	38.44(34.51-42.44)	0.1(0.05-0.14)
Other pharynx cancer	Female	15389(13710-17444)	0.71(0.63-0.8)	37554(33119-42260)	0.87(0.76-0.98)	0.68(0.62-0.74)	12280(10602-14135)	0.57(0.49-0.65)	26190(22518-30492)	0.6(0.52-0.7)	0.11(0.03-0.19)	371722(319623-434977)	16.7(14.36-19.49)	731108(627260-856916)	17.01(14.58-19.95)	-0.06(-0.16-0.03)
	Male	51142(47826-55575)	2.59(2.42-2.81)	129347(116434-141885)	3.23(2.9-3.54)	0.77(0.73-0.81)	39180(35993-43330)	2.04(1.86-2.25)	88017(77952-98654)	2.23(1.98-2.5)	0.27(0.23-0.3)	184340(1092121-130906	57.39(52.92-63.38)	503486(2215501-281858	61.22(54.2-68.85)	0.15(0.1-0.19)
	Both	87583(82236-92717)	2.01(1.9-2.12)	233847(211637-252807)	2.83(2.56-3.06)	1.25(1.12-1.37)	22966(21554-25228)	0.6(0.56-0.66)	45576(41290-48775)	0.57(0.51-0.61)	-0.15(-0.19to-0.11)	667462(613563-732753)	15.55(14.4-17.02)	231841(1113585-132706	14.98(13.55-16.14)	-0.14(-0.18to-0.1)
Thyroid cancer	Female	63796(58659-68290)	2.82(2.61-3.02)	157833(140395-173068)	3.74(3.32-4.1)	0.98(0.85-1.12)	15371(14020-17250)	0.73(0.66-0.82)	26941(23718-29329)	0.62(0.55-0.68)	-0.59(-0.63to-0.56)	438427(386614-489363)	19.48(17.33-21.7)	721999(627829-792461)	16.94(14.72-18.62)	-0.57(-0.62to-0.52)
	Male	23787(22215-25699)	1.16(1.09-1.24)	76014(68232-82923)	1.9(1.71-2.07)	1.89(1.77-2.02)	7596(6939-8498)	0.43(0.4-0.48)	18635(16822-20242)	0.51(0.46-0.55)	0.69(0.61-0.76)	229035(208177-259563)	11.21(10.21-12.64)	509842(461647-553336)	12.93(11.7-14.02)	0.61(0.53-0.68)
